# New Pyrazole/Pyrimidine-Based Scaffolds as Inhibitors of Heat Shock Protein 90 Endowed with Apoptotic Anti-Breast Cancer Activity

**DOI:** 10.3390/ph17101284

**Published:** 2024-09-27

**Authors:** Lamya H. Al-Wahaibi, Mohammed A. I. Elbastawesy, Nader E. Abodya, Bahaa G. M. Youssif, Stefan Bräse, Sara N. Shabaan, Galal H. Sayed, Kurls E. Anwer

**Affiliations:** 1 Department of Chemistry, College of Sciences, Princess Nourah bint Abdulrahman University, Riyadh 11671, Saudi Arabia; lhalwahaibi@pnu.edu.sa; 2 Department of Pharmaceutical Organic Chemistry, Faculty of Pharmacy, Al-Azhar University, Assiut 71524, Egypt; mohamedali.pharm.ast@azhar.edu.eg; 3 Department of Pharmaceutical Chemistry, Faculty of Pharmacy, University of Tabuk, Tabuk 71491, Saudi Arabia; nabodya@ut.edu.sa; 4 Department of Pharmaceutical Organic Chemistry, Faculty of Pharmacy, Assiut University, Assiut 71526, Egypt; 5 Institute of Biological and Chemical Systems, IBCS-FMS, Karlsruhe Institute of Technology, 76131 Karlsruhe, Germany; 6 Department of Chemistry, Faculty of Science (Girls), Al-Azhar University, Nasr City, Cairo 11754, Egypt; saranabil.2259@azhar.edu.eg; 7 Heterocyclic Synthesis Lab., Chemistry Department, Faculty of Science, Ain Shams University, Abbassia, Cairo 11566, Egypt; galal.hosny.sayed@gmail.com (G.H.S.); kurlsekram@sci.asu.edu.eg (K.E.A.)

**Keywords:** pyrimidine, pyrazole, microwave, breast cancer, Hsp90, apoptosis

## Abstract

**Background/Objectives**: Supported by a comparative study between conventional, grinding, and microwave techniques, a mild and versatile method based on the [1 + 3] cycloaddition of 2-((3-nitrophenyl)diazenyl)malononitrile to tether pyrazole and pyrimidine derivatives in good yields was used. **Methods**: The newly synthesized compounds were analyzed with IR, ^13^C NMR, ^1^H NMR, mass, and elemental analysis methods. The products show interesting precursors for their antiproliferative anti-breast cancer activity. **Results**: Pyrimidine-containing scaffold compounds **9** and **10** were the most active, achieving IC_50_ = 26.07 and 4.72 µM against the breast cancer MCF-7 cell line, and 10.64 and 7.64 µM against breast cancer MDA-MB231-tested cell lines, respectively. Also, compounds **9** and **10** showed a remarkable inhibitory activity against the Hsp90 protein with IC_50_ values of 2.44 and 7.30 µM, respectively, in comparison to the reference novobiocin (IC_50_ = 1.14 µM). Moreover, there were possible apoptosis and cell cycle arrest in the G1 phase for both tested compounds (supported by CD1, caspase-3,8, BAX, and Bcl-2 studies). Also, the binding interactions of compound **9** were confirmed through molecular docking, and simulation studies displayed a complete overlay into the Hsp90 protein pocket. **Conclusions**: Compounds **9** and **10** may have apoptotic antiproliferative action as Hsp90 inhibitors.

## 1. Introduction

Breast cancer is the most frequently diagnosed cancer in women and the second leading cause of cancer-related deaths among them [1,2]. Despite ongoing efforts to discover novel treatments, breast cancer mortality rates have recently started to rise. Approximately 2.3 million women were clinically diagnosed with breast cancer in 2020, resulting in a global mortality rate of 30% [3,4]. By the end of 2023, there were 7.8 million women alive who had been diagnosed with breast cancer, indicating a growing global crisis [5,6]. The World Health Organization recently announced that roughly 0.5–1% of breast cancers also develop in men worldwide, affecting women of any age after puberty [7].

Hsp90 (Heat shock protein 90) is crucial in developing breast cancer. It establishes multiple distinct complexes, each with specific groups of co-chaperones that aid in the folding and refolding proteins under stress, transport, and destruction [8]. The therapeutic potential of Hsp90 is enhanced by the fact that cancer cells express it at a level around 2–10 times higher than that of healthy cells [9]. The overexpression of Hsp90 has been suggested as a crucial mechanism by which breast cancer cells develop resistance to various stressors [10]. It has been reported that blocking Hsp90 leads to the breakdown of client proteins, which hinders the growth and reproduction of cancer cells, particularly those linked to breast cancer [11]. The high levels of Hsp90 are associated with increased levels of these client proteins, making Hsp90 a viable target for combating breast cancer or drug development [12,13,14].

Nitrogen-containing heterocyclic compounds are crucial in developing new medications due to their ability to interact effectively with different biological receptors, showing strong binding affinity [15]. Pyrazole and pyrimidine derivatives are the most prominent among different heterocyclic compounds. Most of their derivatives are sourced from plants, but some can be made in a laboratory [16,17,18]. Various research groups have extensively studied their derivatives’ chemistry and biological applications throughout the 20th century [19,20,21].

**Figure 1:**
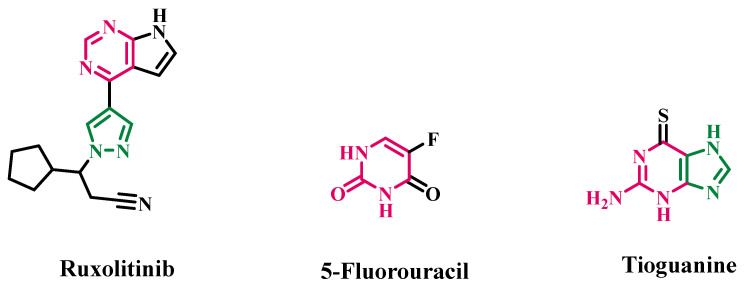
Reported pyrazole and pyrimidine anti-breast cancer drugs.

In recent years, several pyrazole and/or pyrimidine drugs have been identified as possible inhibitors of breast cancer [22,23]. Moreover, utilizing these scaffolds independently, combined, or in conjunction with other chemical components is crucial in developing anticancer drugs [23,24]. Their derivatives have demonstrated impressive outcomes through many mechanisms, including growth inhibition by halting the cell cycle, inducing apoptosis, preventing angiogenesis, interfering with cell migration, and adjusting nuclear receptor sensitivity [25,26,27]. These compounds have shown potential in fighting cancer based on their effects on different types of cancer cells. Their proficiency in developing breast cancer medications is demonstrated by the clinical utilization of drugs such as ruxolitinib [28], 5-florouracil [29], and tioguanine [30], as shown in Figure 1.

**Figure 2:**
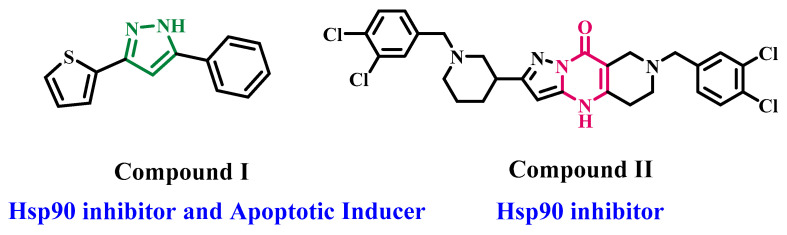
Pyrazole and pyrimidine-based HSP90 inhibitors.

Mohamady et al. [31] described the discovery of 15 pyrazole-based derivatives as HSP90 inhibitors with antiproliferative potential. The newly synthesized compounds were investigated in vitro for antiproliferative activity against two cancer cell lines: breast cancer (MCF-7) and liver cancer (HepG2). Compound **I** (Figure 2) exhibited the most potent antiproliferative action against HepG2 cells, with an IC_50_ value of 0.083 μM. Compound 1 was chosen for additional in vitro tests, where its treatment resulted in cell cycle arrest, specifically in the G2 phase. Compound **I** also resulted in a 7.7-fold elevation in caspase-3 levels. In addition, compound **I** demonstrated the inhibition of Hsp90 (IC_50_ = 2.67 ± 0.18 µM) in a laboratory test and resulted in a 70.8% decrease in Hsp90 levels in a cell-based experiment using HepG2 cells. Molecular dynamic simulation studies revealed that the key interaction responsible for **I**-Hsp90 binding is the hydrogen bond generated between compound **I**’s pyrazole ring and the Asp661 carboxylic group.

Zajec et al. [32] recently conducted a study that discovered a novel type of allosteric Hsp90 C-terminal domain (CTD) inhibitor. These inhibitors are based on a pyrazolo-pyrimidine derivative and have demonstrated antiproliferative action in the SK-N-MC Ewing sarcoma cell line. The improved compounds exhibited heightened anticancer efficacy in the SK-N-MC cell line. The Ewing sarcoma cells were exposed to analog **II** (Figure 2), which is the most potent. As a result, essential Hsp90 client proteins involved in cancer processes, such as EWS:FLI1, CDK4, RAF-1, and IGF1R, were depleted. It is important to note that this depletion occurred without triggering a heat shock response.

**Figure 3:**
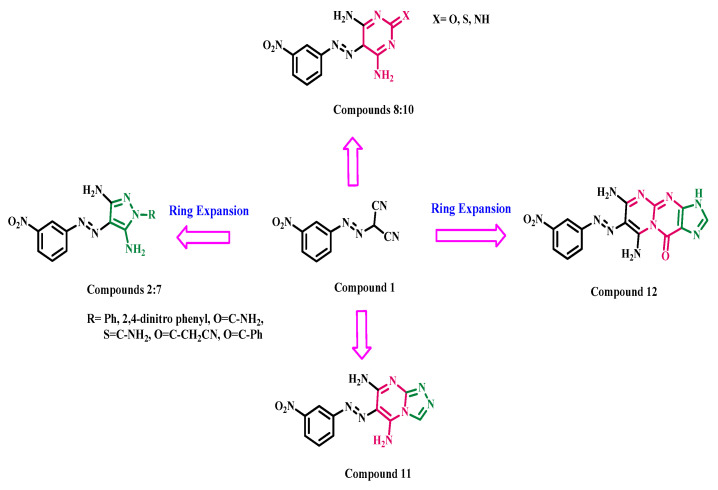
Structures of new target compounds **2**–**12**.

As part of our ongoing inquiry, we developed new compounds (**2**–**12**) based on pyrazole and/or pyrimidine structures, as depicted in Figure 3. These compounds were designed to develop antiproliferative agents that specifically target Hsp90. This approach is based on the previously reported inhibitory activities of pyridine and pyrimidine derivatives against Hsp90 and our prior research [33,34,35,36,37,38] in synthesizing effective targeted anticancer compounds with unique heterocyclic structures. The newly synthesized compounds were evaluated for their capacity to suppress the growth of breast cancer cell lines MCF-7 and MDA-MB231. The IC_50_ of each compound was calculated by comparing it to doxorubicin as the reference drug. Detailed biological and molecular docking studies were conducted on the most potent compounds within Hsp90’s active area. The expression levels of Bax and Bcl-2 and apoptosis via caspase-3 and 8 were investigated.

## 2. Results and Discussion

### 2.1. Chemistry

**Scheme 1:**
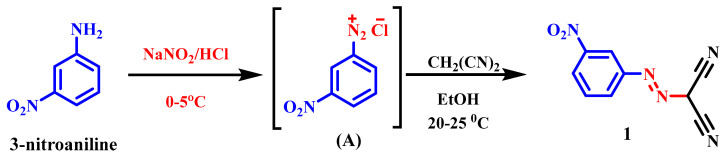
Synthesis of compound **1**.

Diazonium salts are considered one of the most important electrophilic reagents that have been coupled with many active methylene compounds such as acetylacetone, malononitrile, and diethyl malonate in the existence of a few drops of base. The starting material 2-((3-nitrophenyl)diazenyl)malononitrile **1** was synthesized through the treatment of 3-nitroaniline with HCl and NaNO_2_ at 0–5 °C, resulting in the formation of intermediate diazonium salt (**A**). This intermediate then underwent coupling with the active methylene derivative malononitrile in ethanol at a temperature of 20–25 °C, yielding desired product **1** (Scheme 1).

**Scheme 2:**
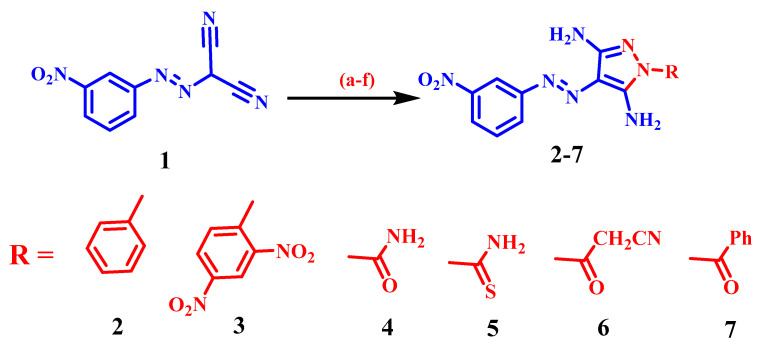
Synthesis of target compounds **2**–**7**.

Target compounds **2**–**7** were obtained via reactions of the dinitrile derivative **1** with phenylhydrazine, 2,4-dinitrophenylhydrazine, semicarbazide, thiosemicarbazide, cyanoacetohydrazide, and/or benzo hydrazide, respectively. The condensed products (**2**–**7**) may be formed by two consecutive additions: first, the nucleophilic addition of the NH_2_ of the hydrazine derivative to one of the nitrile groups of dinitrile **1**, followed by cycloaddition of the NH-R group to the second nitrile group in compound **1** (Scheme 2).

**Reagent and reaction conditions:** (a) PhNHNH_2_, EtOH, reflux 2 h; (b) 2,4-dinitro-phenyl hydrazine, EtOH, reflux 5 h; (c) NH_2_CONHNH_2_.HCl, EtOH, reflux 8 h; (d) NH_2_CSNHNH_2,_ EtOH, reflux 10 h; (e) CNCH_2_CONHNH_2_.HCl, EtOH, reflux 12 h; (f) PhCONHNH_2_, EtOH, reflux 16 h.

**Scheme 3:**
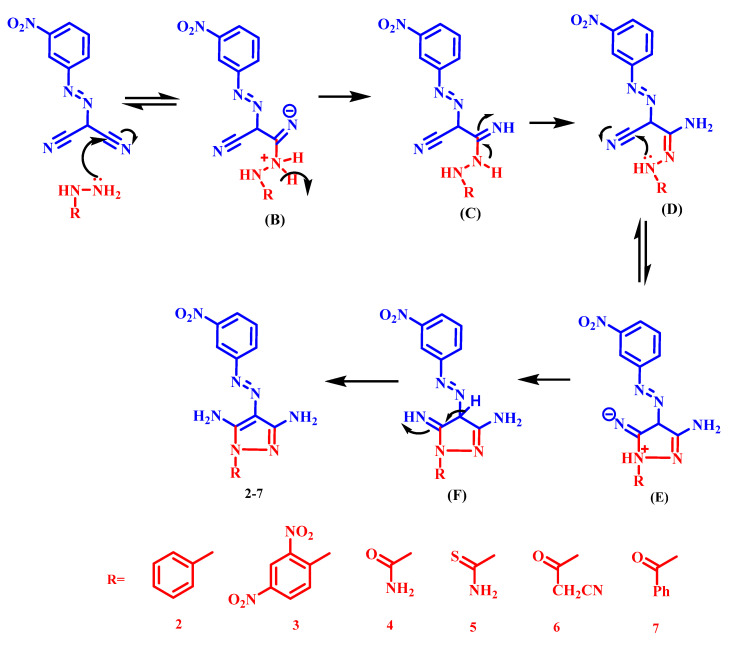
Suggested mechanism for synthesis of pyrazoles **2**–**7**.

The plausible mechanism for forming compounds **2**–**7** was assumed to proceed through intermediates **B** to **F**, as shown in Scheme 3.

The structures of compounds **2**–**7** were determined using IR, ^1^H NMR, ^13^C NMR, and mass spectroscopic methods. The IR spectra of **2** exhibited extra peaks of two NH_2_ groups at 3421, 3321, 3256, and 3160 cm^−1^ with the disappearance of the C≡N group of compound **1**. In addition, the IR spectrum showed C=N and N-O bands at 1522 and 1389 cm^−1^, respectively, confirming the addition reaction and affording target compound **2**. The ^1^H NMR spectrum of **2** displayed distinct singlet signals at 5.97 and 6.34 ppm due to 2NH_2_ protons. The mass spectrum of **2** showed a molecular ion peak at *m*/*z* = 323[M^+^] (13.92%). IR spectra of **3** exhibited extra 2NH_2_ at 3467, 3359, 3197, and 3102 cm^−1^ with the disappearance of the C≡N group of compound **1**. In addition, the IR spectrum showed C=N and N-O bands at 1543 and 1356 cm^−1^, respectively, confirming the addition reaction and affording target compound **3**. The ^1^H NMR spectrum of **3** displayed distinct signals at 11.95 and 13.91 ppm due to four protons of two NH_2_ groups. The mass spectrum of **3** showed a molecular ion peak at *m*/*z* = 413[M^+^] (14.45%).

On the other hand, the IR spectra of **4** exhibited extra C=O at 1650 cm^−1^. The ^1^H NMR spectrum of **4** displayed distinct signals at 5.87 and 7.05 ppm due to 3NH_2_ protons. Its ^13^C-NMR spectrum displayed distinct signals at 152.9 ppm for the C=O group. The mass spectrum of **4** showed a molecular ion peak at *m*/*z* = 290[M^+^] (4.43%), while the IR spectra of **5** exhibited an extra broad band of 3NH_2_ at 3426–2969 cm^−1^. In addition, the IR spectrum showed N=N, N-O, and C=S bands at 1521, 1347, and 1279 cm^−1^, respectively. The ^1^H-NMR spectrum of **5** displayed distinct signals at 6.90 and 7.20 ppm due to 3NH_2_ protons. Its ^13^C-NMR spectrum displayed distinct signals at 186.1 ppm for the C=S group. The mass spectrum of **5** showed a molecular ion peak at *m*/*z* = 306[M^+^] (14.51%).

However, the IR spectra of **6** exhibited extra bands of CN and C=O at 2216 and 1650 cm^−1^, respectively. The ^1^H NMR spectrum of **6** displayed distinct signals at 3.61 pm due to CH_2_CN protons. Its ^13^C NMR spectrum displayed distinct signals at 161.4 ppm for the C=O group. The mass spectrum of **6** showed a molecular ion peak at *m*/*z* = 314[M^+^] (11.67%), while the IR spectra of **7** exhibited an extra band of C=O at 1670 cm^−1^. Its ^13^C NMR spectrum displayed distinct signals at 177.8 ppm for the C=O group. The mass spectrum of **7** showed a molecular ion peak at *m*/*z* = 351[M^+^] (61.83%).

**Scheme 4:**
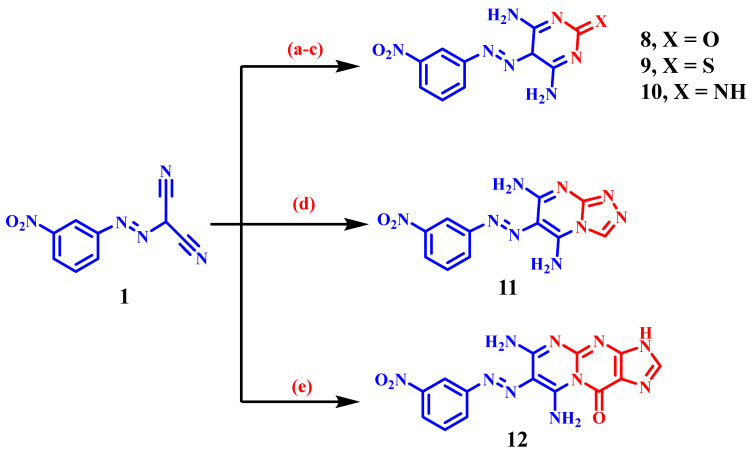
Synthesis of target compounds **8**–**12**.

Compounds **8**–**12**, exhibiting a pyrimidine group, were synthesized by reacting dinitrile derivative **1** with urea, thiourea, guanidine hydrochloride, 3-amino-1,2,4-triazole, and/or guanine, as shown in Scheme 4.

The IR spectra of **8** exhibited an extra broad band of 2NH_2_ at 3400–3072 cm^−1^ with the disappearance of the C≡N group of compound **1**. In addition, the IR spectrum showed the C= O band at 1664 cm^−1^, confirming the addition reaction and affording target compound **8**. The ^1^H NMR spectrum of **8** displayed a distinct signal at 7.18 ppm due to 4NH_2_ protons. Its ^13^C NMR spectrum displayed distinct signals at 163.9 ppm for the C=O group. The mass spectrum of **8** showed a molecular ion peak at *m*/*z* = 275[M^+^] (14.57%). Meanwhile, the IR spectra of **9** exhibited extra 2NH_2_ at 3379, 3276, 3186, and 3129 cm^−1^ with C=N and C=S bands at 1601 and 1262 cm^−1^, respectively, confirming the addition reaction and affording target compound **9**. Its ^13^C NMR spectrum displayed distinct signals at 184.3 ppm for the C=S group. The mass spectrum of **9** showed a molecular ion peak at *m*/*z* = 291[M^+^] (10.07%). Also, the IR spectra of **10** exhibited extra NH at 3093 cm^−1^. The ^1^H NMR spectrum of **10** displayed distinct signals at 7.16 and 13.46 ppm due to 4NH_2_ and NH protons, respectively. The mass spectrum of **10** showed a molecular ion peak at *m*/*z* = 274[M^+^] (30.09%). However, the IR spectra of **11** exhibited extra NH_2_ at 3472, 3429, 3318, and 3201 cm^−1^. The ^1^H NMR spectrum of **11** displayed distinct signals at 6.04 ppm due to 4 NH_2_ protons. The mass spectrum of **10** showed a molecular ion peak at *m*/*z* = 299[M^+^] (13.60%). Finally, the IR spectra of **12** exhibited an extra broad band of NH_2_ and C=O at 3319–2691 and 1697 cm^−1^, respectively. The ^1^H NMR spectrum of **12** displayed distinct signals at 11.98 ppm due to NH protons. The mass spectrum of **12** showed a molecular ion peak at *m*/*z* = 366[M^+^] (22.31%).

**Reagent and reaction conditions:** (a) NH_2_CONH_2_, EtOH, reflux 4 h; (b) NH_2_CSNH_2_ hydrazine, EtOH, reflux 5 h; (c) NH_2_C(NH)NH_2_.HCl, EtOH, reflux 5 h; (d) 3-amino-1,2,4-triazole_,_ EtOH, reflux 10 h; (e) guanine, CH_2_CONHNH_2_, EtOH, reflux 12 h.

**Figure 4:**
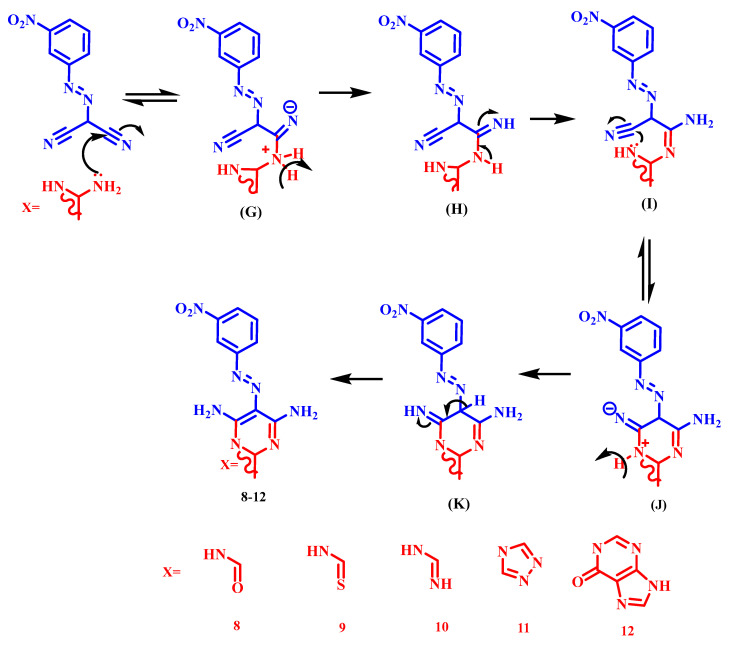
Suggested mechanism for synthesis of pyrimidines **8**–**12**.

The pyrimidine derivatives (**8**–**12**) are formed through a series of reactions starting with the nucleophilic addition of NH_2_ from the previously mentioned reagents to one of the nitrile groups in compound **1**. This forms intermediate (**G**), which undergoes an H^+^ shift to produce intermediate (**H**). Subsequently, a cycloaddition reaction occurs between the second NH-R group of 1,3-diamino derivatives or the NH group in cyclic amino compounds with the second CN group in compound **1**, resulting in the formation of compounds **8**–**10**, **11**, and **12**, respectively, through intermediates (**I**–**K**). The proposed pathway for converting compound **1** into the matching 3,5-diamino pyrimidines **8**–**12** is illustrated in Figure 4.

The IR spectra of compounds **8**–**12** did not show a CN band or the presence of amino groups. Furthermore, the IR spectra of compounds **8** and **12** exhibited distinctive peaks corresponding to the C=O group at 1664 and 1697 cm^−1^, respectively. Compound **9** exhibited a peak of the C=S group at 1262 cm^−1^. The ^1^H NMR, ^13^C NMR, and mass spectra confirm the hypothesized structures.

The reactions to obtain our desired derivatives were carried out utilizing three distinct methods: grinding, microwave irradiation, and traditional procedures [39,40,41,42,43]. TLC was utilized as a guiding tool to achieve the reaction’s goals. Although the same amounts of reactants were employed in all three procedures, discrepancies were found in the time needed for a complete reaction and the yields produced in each approach.

The variations in reaction times and yields of the produced substances using grinding, microwave irradiation, and old conventional methods are documented in Table 1. The yield economy “YE” was utilized to determine which strategy is more advantageous for achieving the reaction. The yield economy can be determined using the following equation:

$$YE=\frac{yield\%}{Reaction\;time\;“min”}$$

The Reaction Mass Efficiency “RME” was also estimated for each approach:

$$RME=\frac{Wt\;of\;isolated\;product}{Wt\;of\;reactants}$$

Another metric for comparing the three methods is optimum efficiency “OE”, which can be calculated as follows:

$$OE=\frac{RME}{AE}\times 100$$

where “AE” represents atomic economy, while “RME” represents the maximum reaction efficiency. The yield economy “YE” is a standard parameter used to quantify the transformation efficiencies of the three procedures for synthesizing the target pyrazole derivatives. “AE” is equivalent to the three variable approaches in synthesizing the desired products.

Table 1 depicts the physical tools used to distinguish between three different (conventional, grinding, and microwave) ways to synthesize derivatives **2**–**12**. The microwave and grinding reactions produced a higher percentage yield than the traditional procedures. Notably, the percentage yield obtained by microwave synthesis approached stoichiometric completion. In addition, the microwave and grinding processes exhibited superior optimal efficiency and reaction mass efficiency compared to conventional reactions.

### 2.2. Biological Assays

#### 2.2.1. In Vitro Antiproliferative Activity

The in vitro antiproliferative effect of 11 target compounds (**2**–**12**) on two breast cancer cell lines (MCF-7 and MDA-MB231) was assessed using the MTT assay [44,45,46]. The IC_50_ values for each compound were computed, as indicated in Table 2. The well-known chemotherapeutic medicines doxorubicin [47] and cisplatin [48] were utilized as reference medications.

In general, an examination of the data in Table 2 reveals that the newly synthesized compounds **2**–**12** exhibit more effectiveness against the MDA-MB231 cancer cell line than the MCF-7 breast cancer cell line. The IC_50_ values for **2**–**12** against MDA-MB231 vary from 4.70 to 72.50 µM, whereas the IC_50_ values against the MCF-7 cell line range from 6.20 to 247.30 µM.

In terms of activity against the MCF-7 breast cancer cell line, compounds **2**, **8**, **9**, and **10** were the most potent derivatives, with IC_50_ values of 6.20, 33.60, 26.10, and 7.70 µM, respectively, making compounds **2**, **9**, and **10** more potent than the reference doxorubicin (IC_50_ value = 33.20 µM), but in all cases, the compounds were less potent than the reference cisplatin (IC_50_ = 3.70 µM). Compound **2** (R = phenyl, pyrazole scaffold) exhibited the highest potency among all the synthesized compounds against the breast cancer MCF-7 cell line. The IC_50_ value of compound **2** was 6.20 µM, which was 5-fold more potent than the reference doxorubicin (IC_50_ value = 33.20 µM) but 1.7 times less potent than cisplatin (IC_50_ value = 3.70 µM).

The replacement of the phenyl group at N^1^ in the pyrazole moiety with different groups such as the 2,4-dinitrophenyl group as in compound **3** (R = 2,4-dinitrophenyl, pyrazole scaffold), the carboxamide group as in compound **4** (R = CONH_2_, pyrazole scaffold), the carbothioamide group as in compound **5** (R = CSNH_2_, pyrazole scaffold), the oxo-propane nitrile moiety as in compound **6** (R = COCH_2_CN, pyrazole scaffold), or with the phenyl methanone moiety as in compound **7** (R = COPh, pyrazole scaffold) led to a significant decrease in the antiproliferative activity. The IC_50_ values for compounds **3**, **4**, **5**, **6**, and **7** were 247.30, 236.40, 85.90, 234.50, and 221.40 µM, respectively. These data suggest that the substitution pattern at the nitrogen atom in position 1 of the pyrazole structure significantly impacts the antiproliferative activity of compounds **2**–**7**. The presence of a phenyl group is well tolerated and enhances the activity, followed by the carbothioamide group. Other groups are not favored for activity.

Compound **10** (X = NH, pyrimidine scaffold), which has a pyrimidine scaffold and an NH group, showed the second highest activity level against the MCF-7 breast cancer cell line. Compound **10** had an IC_50_ value of 7.70 µM, which was similar to compound **2** with an IC_50_ value of 6.20 µM. Replacement of the NH group in compound **10** with other groups, such as the oxygen atom as in compound **8** (X = O, pyrimidine scaffold) or with the sulfur atom as in compound **9** (X = S, pyrimidine scaffold), resulted in a marketed decrease in the antiproliferative activity with IC_50_ values of 33.60 and 26.10 µM, respectively. Finally, ring expansion to give the 1,2,4-triazole derivative, compound **11,** or purine derivative, compound **12,** resulted in weak activity with IC_50_ values of 237.20 and 244.40 µM, respectively. Compounds **11** and **12** can be categorized among the least potent derivatives, suggesting that the size of the ring plays a critical role in determining the effectiveness of these novel compounds.

Concerning the efficacy of compounds **2**–**12** against the breast cancer MDA-MB231 cell line, it was found that these compounds exhibited greater potency against this specific cancer cell line compared to the MCF-7 breast cancer cell line. The IC_50_ values ranged from 4.70 to 72.50 µM. Furthermore, it was observed that pyrimidine-based derivatives (**8**–**12**) demonstrated higher potency than pyrazole-based derivatives (**2**–**7**), as shown in Table 2.

Compound **9**, which has a pyrimidine scaffold and sulfur (X = S), showed the highest potency against the MDA-MB231 breast cancer cell line. Its IC_50_ value was 4.70 µM, which is comparable to the reference drug doxorubicin (IC_50_ = 3.20 µM). Substituting the sulfur atom in compound **9** with an NH group, as seen in compound **10** (X = NH and pyrimidine scaffold), reduced the antiproliferative activity against the MDA-MB231 breast cancer cell line. Compound **10** exhibited an IC_50_ value of 10.70 µM, being 2 times less potent than compound **9**.

Ultimately, all variables impacting the relationship between the structure and activity of compounds **2**–**12** against the MCF-7 cell line also hold when considering the MDA-MB231 cell line. It has been observed that the presence of an NH group and a sulfur atom on the pyrimidine ring results in the highest activity level. Conversely, expanding the pyrimidine ring into triazole-pyrimidine or purine derivatives significantly decreases the activity.

#### 2.2.2. Effect on Normal Cells

The most promising antiproliferative agents, **9** and **10,** were subsequently investigated for their cytotoxicity against the normal breast (MCF-10A) cell line [46]. This study revealed that compounds **9** and **10** had IC_50_ values of 30.70 and 37.30 µM, respectively (Table 3). These values were higher than those obtained against the breast cancer MDA-MB231 cell line (4.70 and 10.70, respectively, Table 2). Consequently, both compounds exhibited a selectivity index of 6.5 and 3.5, respectively.

#### 2.2.3. Hsp90 Inhibitory Activity

Compounds **9** and **10**, the most effective antiproliferative derivatives, were studied further for their inhibitory action on Hsp90, a potential molecular target for their anti-breast cancer action, compared to novobiocin as a reference drug [47,49]. The outcomes of this in vitro assay test have been powerfully supplemented by the results of the antiproliferative assay. Compounds **9** and **10** efficiently inhibited Hsp90 with IC_50_ values of 2.44 and 7.30 µM, respectively. This is 2- and 6.5-fold less potent than the reference medication, novobiocin (IC_50_ = 1.14 µM), as shown in Table 4. These findings revealed that compounds **9** and **10** are prospective antiproliferative candidates that may act as Hsp90 inhibitors, requiring additional structural modification to yield more potent derivatives.

#### 2.2.4. Activation of Caspases Cascade

**Figure 5:**
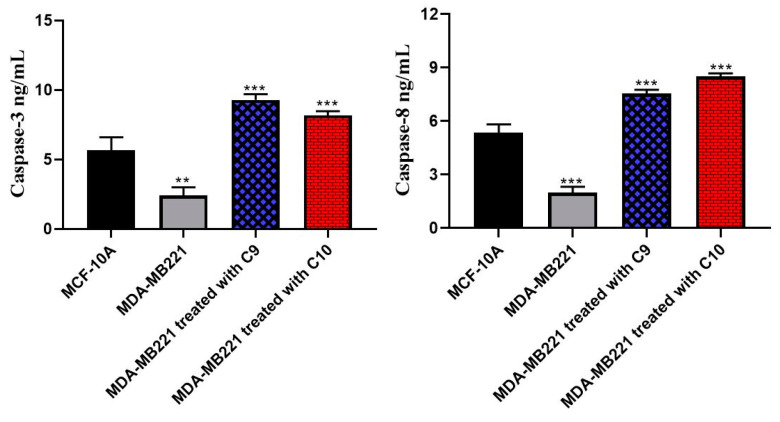
Graphical representation of the effect of compounds **9** and **10** on apoptotic caspases 3 and 8. ** when significance *p* < 0.01, *** when significance *p* < 0.001.

Caspase activation starts and controls apoptosis. Caspase-3, a common caspase, cleaves numerous cell proteins, causing apoptosis [50,51,52]. The impact of compounds **9** and **10** on caspase-3 activation was assessed and compared to the normal breast MCF-10A cell as a negative control and the breast cancer MDA-MB-231 cell as a positive control. The results indicated that compounds **9** and **10** exhibited a significant upregulation of caspase 3, with fold increases of 3.87 and 3.40, respectively, compared to the control group (Table 5 and Figure 5).

In addition, we assessed the impact of compounds **9** and **10** on caspase-8. Compound **9** caused a 3.81-fold increase in caspase-8 levels, whereas compound **10** resulted in a 4.29-fold increase. These observations suggest that **9** and **10** can activate intrinsic and extrinsic caspase, making them potential apoptosis inducers.

#### 2.2.5. BAX and Bcl-2 Assays

**Figure 6:**
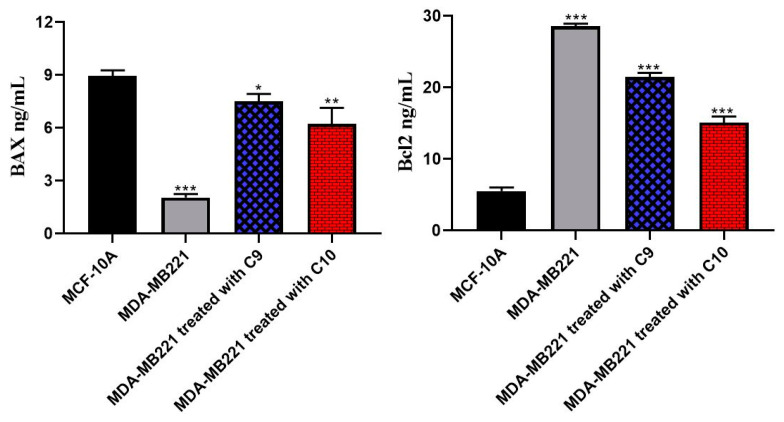
Graphical representation of the effect of compounds **9** and **10** on apoptotic Bax and anti-apoptoticBcl-2. * When significance *p* < 0.05, ** when significance *p* < 0.01, *** when significance *p* < 0.001.

Bax and Bcl-2 are primary regulators of the apoptosis process, maintaining equilibrium between living and deceased normal cells [53]. The deregulation or abnormality of these proteins is a frequent characteristic that leads to the uncontrolled development of cancer cells [54]. Therefore, the protein expression of apoptotic regulators was assessed using ELISA analysis [45]. The results are shown in Table 6 and Figure 6. Compounds **9** and **10** were applied to breast cancer MDA-MB-231 cells at their IC_50_ concentrations for 24 h. We used a normal MCF-10A cell at the same concentration as a positive reference. As a result, apoptotic protein (Bax) levels increased by 3.64 and 3.02 times, respectively, compared to the control group.

Similarly, the levels of the anti-apoptotic Bcl-2 reduced by 1.33-fold and 1.89-fold, respectively, compared to the control. These findings showed that compounds **9** and **10** enhanced MDA-MB-231 cells’ intrinsic apoptotic mechanism. This was carried out by increasing Bax and decreasing anti-apoptotic Bcl-2.

#### 2.2.6. CD1 Inhibition Activity

**Figure 7:**
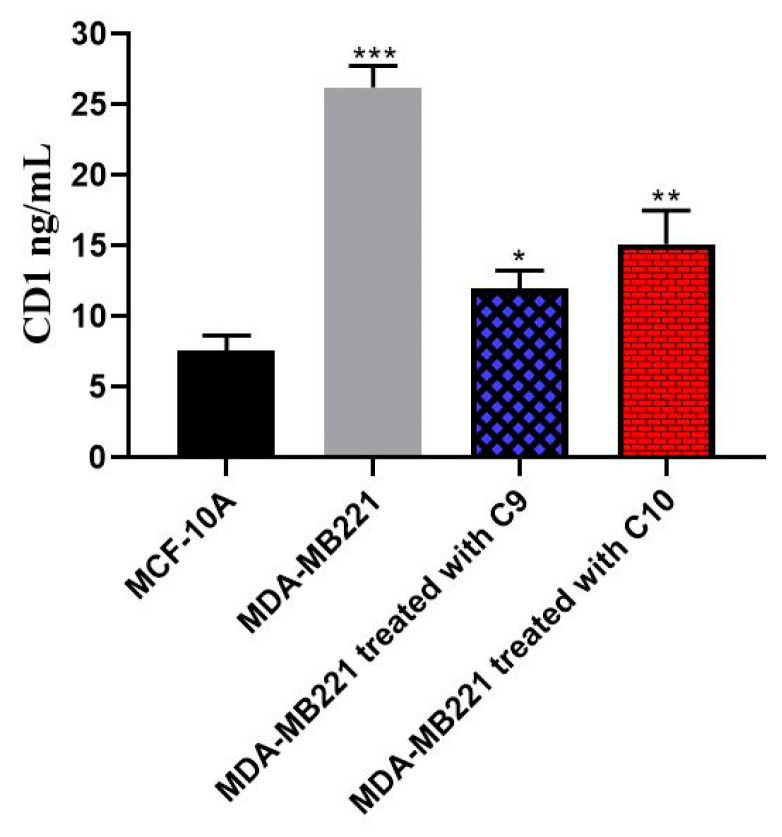
CD1 inhibitory activity of compounds **9** and **10** and controls. * When significance *p* < 0.05, ** when significance *p* < 0.01, *** when significance *p* < 0.001.

The rate-limiting D-type cyclins coordinate cell cycle activation and are necessary for G1 cell cycle progression [55]. D-type cyclins form complexes with and stimulate the activity of cyclin-dependent kinases Cdk4 and Cdk6, which subsequently phosphorylate the retinoblastoma protein Rb [56]. When Rb is phosphorylated, it activates E2F transcription factors, stimulating the production of genes involved in the S-phase of the cell cycle, leading to cell cycle progression [57]. Cyclin D1 increases in early G1 assist in titrating Kip/Cip proteins away from cyclin E/Cdk2 complexes, accelerating cell cycle progression. Increased CD1 levels convey mitogen signals to the Rb/E2F pathway, which regulates apoptosis [58]. In this investigation, the most active compounds, **9** and **10**, inhibited CD1 levels significantly in breast cancer MDA-MB-231 cells compared to the untreated control [59]. Compound **9** was the most active, inhibiting the protein by 55%, while compound **10** downregulated CD1 levels by 42.3%, suggesting that each compound may halt the cell cycle in the G1 phase (Figure 7 and Table 7).

### 2.3. Molecular Docking Studies

#### 2.3.1. Docking Study

This work involved docking compound **9**, which exhibited the highest activity level, with the Hsp90 receptor. The co-crystallized ligand of the crystal protein (PDB code: 2XDX) [60] obtained from the RCSB was utilized to generate the binding sites. The molecular docking process was carried out with the docking algorithms of Autodock Vina 4.0 [37]. The docking scores, which represent the affinity interaction energy, were measured for the most suitable poses with the active site of the Hsp90 protein, Table 8. The 3D orientation was also created using the Discovery Studio 2016 visualizer program [61] (refer to Supplementary S1 in Supplementary file for more details).

**Figure 8:**
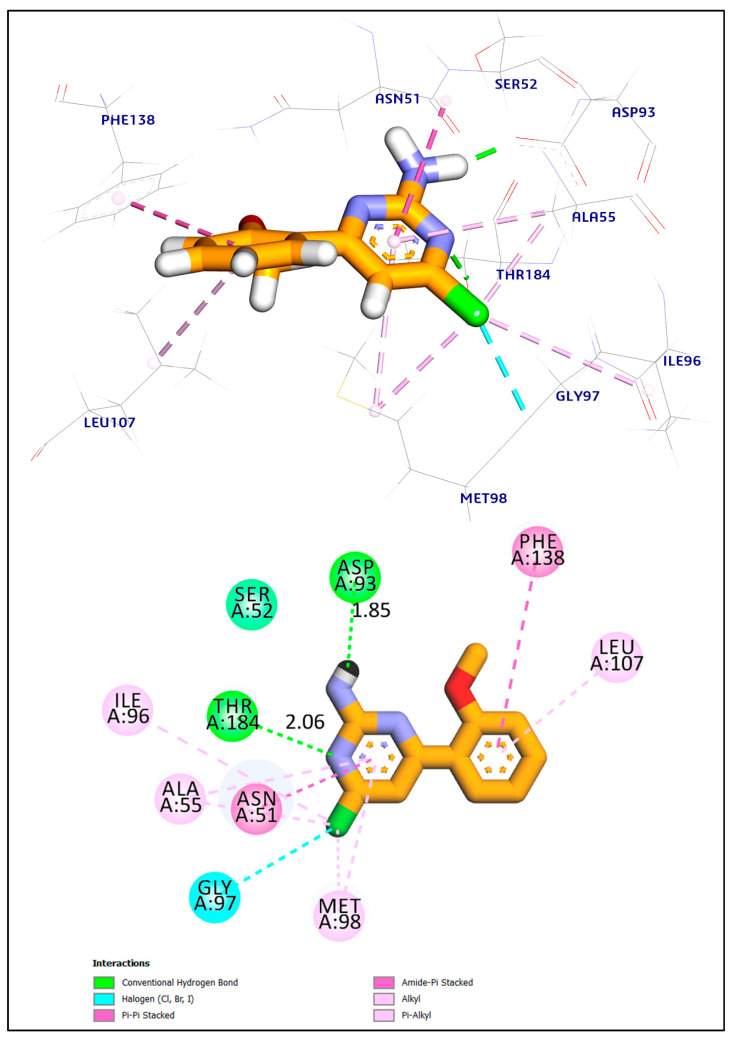
Three-dimensional and 2D figures of the co-crystalized ligand docked in HSP90.

**Figure 9:**
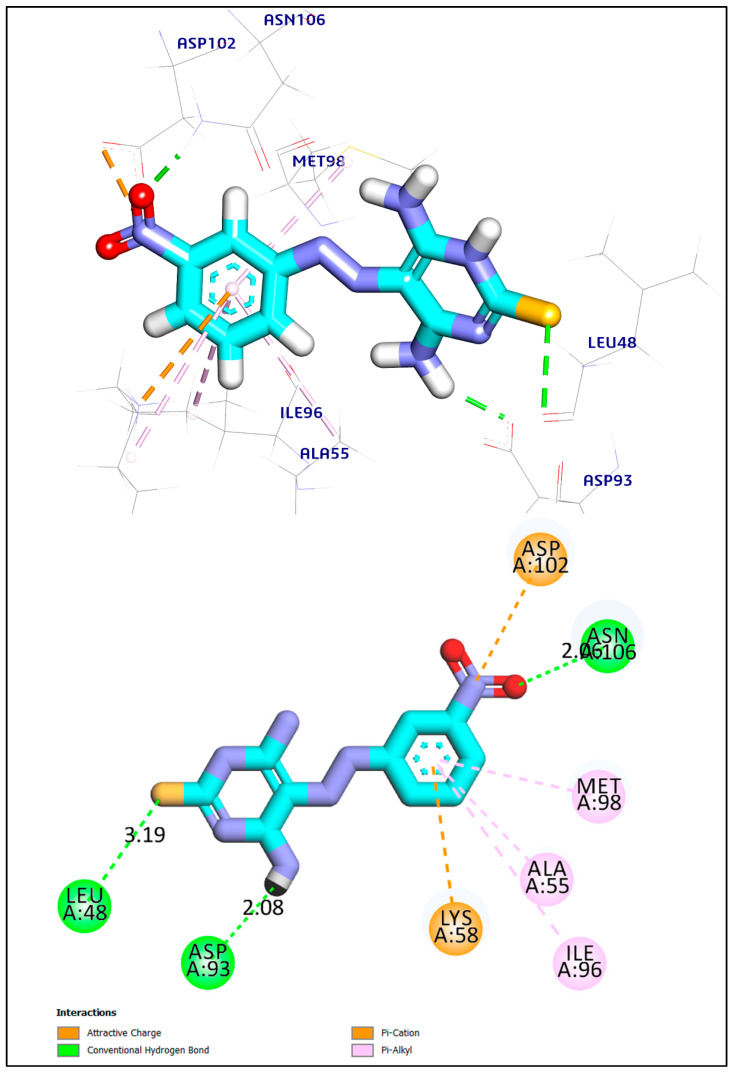
Three-dimensional and 2D figures of compound **9** docked in HSP90.

The co-crystalized ligand demonstrated a binding mode with an affinity score of −6.75 kcal/mol against Hsp90. The ligand in its co-crystallized form established a total of eight hydrophobic interactions, specifically π-π and π–alkyl interactions, with Leu107, Phe138, Met98, Asn51, Ala55, and Ile96. In addition, it established two hydrogen bonds with Asp93 and Thr184, with distances measuring 1.85 and 2.06 Å, respectively (Figure 8). However, compound **9** had a binding energy of −6.45 kcal/mol towards Hsp90. The compound had five hydrophobic π–cation and π–alkyl interactions with Met98, Ala55, Ile96, and Lys58. Additionally, it formed three hydrogen bonds and one ionic interaction with Asn106, Asp93, Leu98, and Asp102, with distances of 2.06, 2.08, and 3.19 Å, respectively (Figure 9).

#### 2.3.2. Molecular Dynamics (MD) Simulation Study

To study the compounds’ stability with the best docking orientation in Hsp90 active sites, MD simulations were conducted for 100 ns. The obtained root-mean-square deviations (RMSDs) for the complexes and the ligands concerning their original positions within the active site were analyzed and are reported in this paper. Frontier compound interactions were also analyzed and evaluated in detail. Finally, the MM-GBSA free binding energy was estimated for the tested complex during the simulation trajectories [62].

##### Protein and Ligand RMSD Analysis

**Figure 10:**
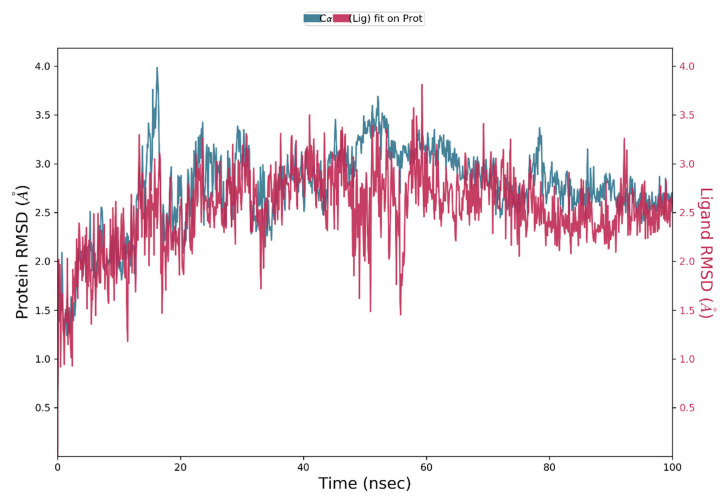
The RMSD of compound **9** Hsp90 complex for 100 ns.

Compound **9** was selected for MD simulation, complexed with Hsp90. The conformational stability of the proteins was monitored through the C*α* atoms of the protein concerning their initial position. As shown in Figure 10, the compound **9** Hsp90 complex showed high stability with an RMSD value within 2.50 Å, an acceptable value below 3.00 Å.

**Figure 11:**
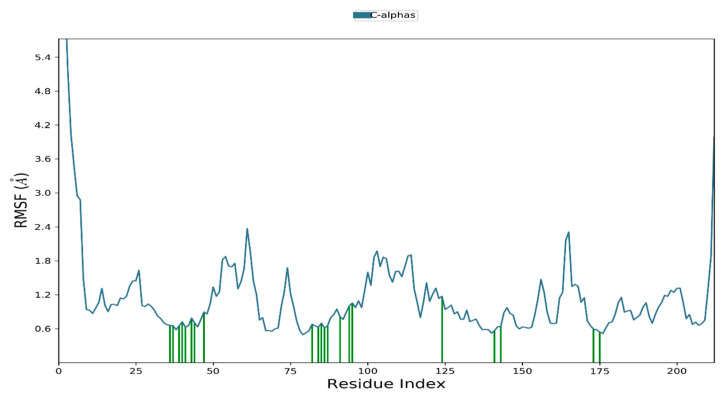
The RMSF of compound **9** Hsp90 complex for 100 ns.

The RMSF was measured for ligands’ atoms based on the initial position of the protein active site. The compound **9** Hsp90 complex showed stability inside the target pocket over time with no major fluctuation (Figure 11). The Hsp90 protein showed conformational changes over the MD simulation period that appeared to have minor fluctuations from 50 to 60 ns (Figure 11).

**Figure 12:**
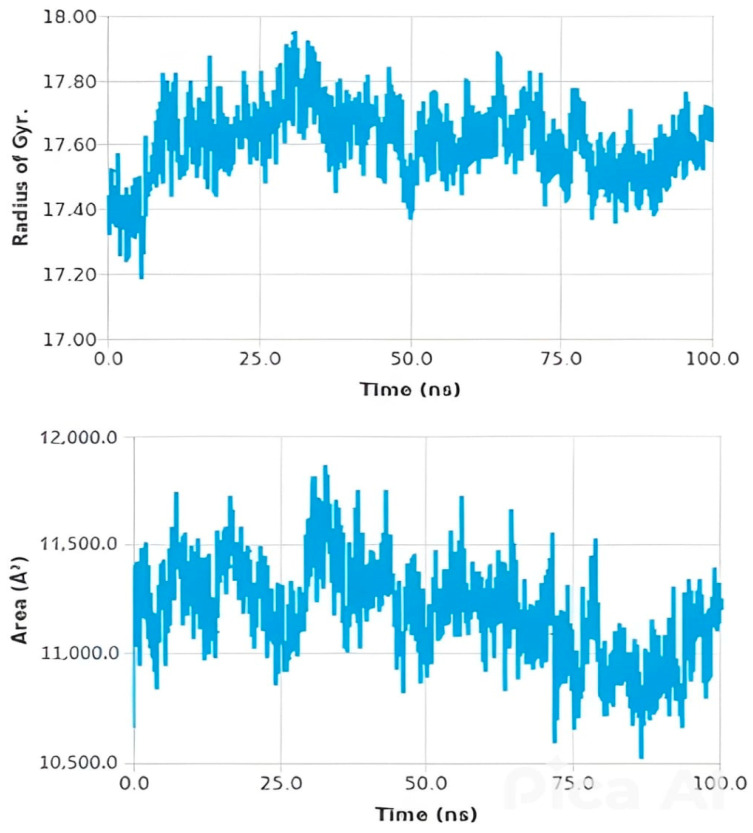
The Rg and SASA of compound **9** Hsp90 complex for 100 ns.

On the other hand, the compactness of the complex was represented by the radius of gyration (Rg). The lower degree of fluctuation throughout the simulation period indicates the greater compactness of a system. The Rg of the compound **9** Hsp90 complex was the same as that of the starting period. The interaction between protein–ligand complexes and solvents was measured by the solvent-accessible surface area (SASA) over the simulation period. So, the SASA of the complex was calculated to analyze the extent of the conformational changes that occurred during the interaction. Interestingly, the compound **9** Hsp90 complex featured a reduction in the surface area, showing a relatively lower SASA value than the starting period (Figure 12).

##### Histogram and Heat Map Analysis

**Figure 13:**
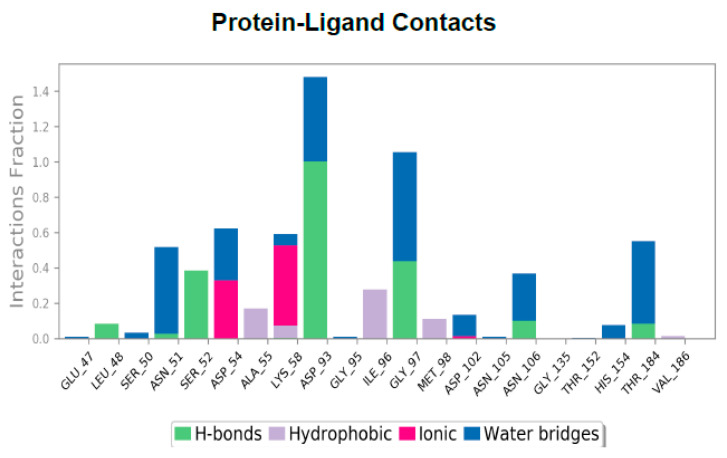
Histogram describing the binding interactions of compound **9** Hsp90 complex during the simulation time (100 ns).

Compound **9** showed high stability within the Hsp90 active pocket; their interactions are discussed in detail. Compound 9 formed H-bond interactions with the following residues, Leu48 (~10%), Ser52 (~75%), Asp93 (~100%), Gly97 (~50%), Asn106 (~10%), and Thr187 (~10%), as presented in Figure 13.

**Figure 14:**
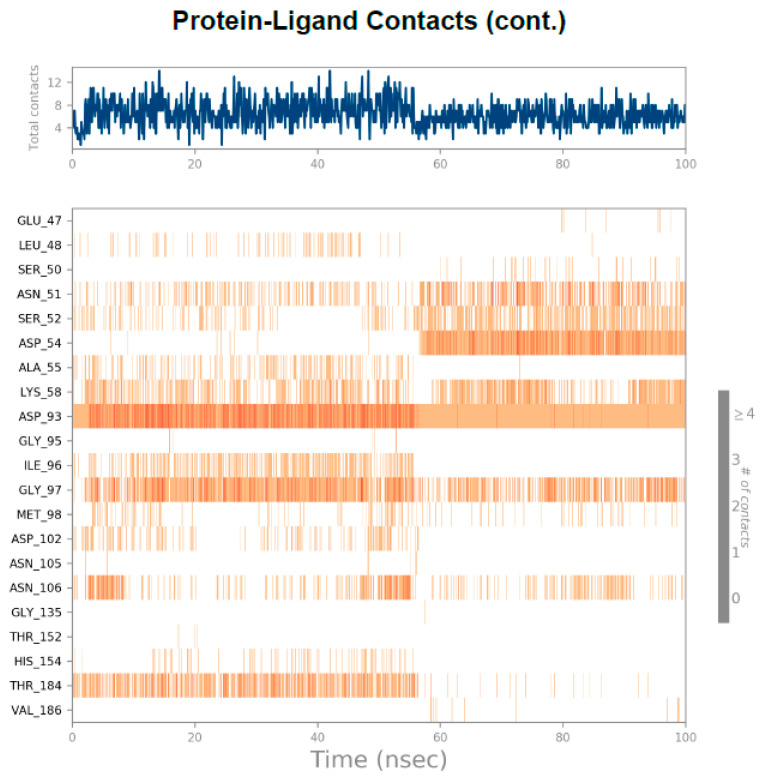
Heat map describing the total interactions within Compound 9 HSP90 complex during the 100 ns.

Compound **9** was able to form hydrophobic interactions with residues Ala55 (~55%), Lys58 (~55%), Ile96 (~30%), and Met98 (~15%) and ionic interactions with Asp54 (~30%) and Lys45 (~45%). Another method to monitor these interactions involves plotting the number of interactions concerning time. A heat map (Figure 14) indicates the number of interactions at each Compound **9** Hsp90 complex frame, whereas the dark color indicates more interactions. The heat map figures showed that the highest protein conformations formed up to eight hydrogen bonds of the compound **9** HSP90 complex.

## 3. Materials and Methods

### 3.1. Chemistry

General details: See Supplementary S1 (Supplementary File).

#### 3.1.1. General Synthetic Method of Derivatives (**2**–**12**)

A solution of compound **1** (2.15 g, 0.01 mol) in ethanol (20 mL) was refluxed for 2–16 h with phenylhydrazine (1.08 mL, 0.01 mol), 2,4-dinitrophenyl hydrazine (1.98 mL, 0.01 mol), semicarbazide hydrochloride (1.11 g, 0.01 mol), thiosemicarbazide (0.91 g, 0.01 mol), cyanoacetohydrazide (0.99 g, 0.01 mol), benzo hydrazide (1.36 g, 0.01 mol), urea (0.60 g, 0.01 mol), thiourea (0.76 g, 0.01 mol), guanidine hydrochloride (0.95 g, 0.01 mol), 3-amine-1,2,4-triazole (0.84 g, 0.01 mol), and guanine (1.51 g, 0.01 mol) separately. After cooling, the obtained solid was filtered, washed three times with ethanol (10 mL), and recrystallized from the appropriate solvent to afford compounds **2**–**12**, respectively.

##### 4-((3-Nitrophenyl)diazenyl)-1-phenyl-1*H*-pyrazole-3,5-diamine (**2**)

Brown crystals from methanol; m.p. 222–224 °C. IR (cm^−1^) ʋ: 3421, 3321, 3256, 3160 (NH_2_), 1601 (C=N), 1565 (C=C), 1522 (N=N), 1389 (N-O). ^1^H-NMR (300 MHz, DMSO-d_6_) δ (ppm): 5.97 (s, 2H, NH_2_, D_2_O-exchangeable), 6.34 (s, 2H, NH_2_, D_2_O-exchangeable), 7.55 (d, 1H, Ar-H), 8.59 (d, 2H, Ar-H), 7.23 (d, 1H, Ar-H), 7.88 (s, 1H, Ar-H), 8.02 (t, 1H, Ar-H), 8.19 (d, 2H, Ar-H), 8.43 (s, 1H, Ar-H).^13^C NMR (100 MHz, DMSO-d_6_) δ (ppm): 112.4, 113.3, 114.9, 116.5, 118.8, 121.2, 122.8, 126.7, 129.5, 129.8, 130.6, 130.8, 139.2, 149.3, and 155.0. MS (*m*/*z*): 323 (M^+^, 13.92%). Anal. Calcd for C_15_H_13_N_7_O_2_ (323): C, 55.73; H, 4.02; N, 30.34. Found: C, 55.69; H, 3.98; N, 30.51.

##### 1-(2,4-Dinitrophenyl)-4-((3-nitrophenyl)diazenyl)-1*H*-pyrazole-3,5-diamine (**3**)

Reddish-brown crystals from acetic acid; m.p. > 300 °C. IR (cm^−1^) ʋ: 3467, 3359, 3197, 3102 (NH_2_), 1619 (C=N), 1579 (C=N), 1579 (C=C), 1543 (N=N), 1356 (N-O). ^1^H-NMR (300 MHz, DMSO-d_6_) δ (ppm): 7.55 (s, 1H, Ar-H), 7.59 (t, 1H, Ar-H), 7.80 (d, 1H, Ar-H), 7.86 (d, 1H, Ar-H), 8.02 (s, 1H, Ar-H), 8.11 (d, 1H, Ar-H), 8.19 (d, 1H, Ar-H), 11.95 (s, 2H, NH_2_, D_2_O-exchangeable), 13.91 (s, 2H, NH_2_, D_2_O-exchangeable). ^13^C NMR (100 MHz, DMSO-d_6_) δ (ppm):110.0, 110.5, 110.8, 111.3, 11.4, 118.2, 118.9, 119.9, 122.1, 122.6, 130.7, 131.1, 143.8, 148.9, and 162.8. MS (*m*/*z*): 413 (M^+^, 14.45%). Anal. Calcd for C_15_H_11_N_9_O_6_ (413): C, 43.58; H, 2.66; N, 30.51. Found: C, 43.44; H, 2.79; N, 30.50.

##### 3,5-Diamino-4-((3-nitrophenyl)diazenyl)-1*H*-pyrazole-1-carboxamide (**4**)

Orange crystals from ethanol; m.p. 250–252 °C. IR (cm^−1^) ʋ: 3414, 3307, 3201 (NH_2_), 1650 (C=O), 1616 (C=N), 1607 (C=C), 1526 (N=N), 1346 (N-O). ^1^H-NMR (300 MHz, DMSO-*d_6_*) δ (ppm): 5.87 (s, 2H, NH_2_, D_2_O-exhangeable), 7.05 (s, 4H, 2NH_2_, D_2_O-exhangeable), 7.59 (d, 2H, Ar-H), 8.09 (t, 1H, Ar-H), 8.43 (d, 2H, Ar-H), 8.78 (s, 1H, Ar-H).^13^C NMR (100 MHz, DMSO-d_6_) δ (ppm): 109.4, 123.0, 127.7, 129.1, 130.7, 130.9, 149.2, 149.3, 149.4 and 152.9. MS (*m*/*z*): 290 (M^+^, 4.43%). Anal. Calcd for C_10_H_10_N_8_O_3_ (290): C, 41.38; H, 3.45; N, 38.62. Found: C, 41.47; H, 3.22; N, 38.71.

##### 3,5-Diamino-4-((3-nitrophenyl)diazenyl)-1*H*-pyrazole-1-carbothioamide (**5**)

Orange crystals from ethanol; m.p. 278–280 °C. IR (cm^−1^) ʋ: 3426–2969 (broad, NH_2_), 1621 (C=N), 1552 (C=C), 1521 (N=N), 1347 (N-O), 1279 (C=S).^1^H-NMR (300 MHz, DMSO-*d_6_*) δ (ppm): 6.90 (s, 2H, NH_2_, D_2_O-exchangeable), 7.20 (s, 4H, 2NH_2_, D_2_O-exchangeable), 7.58 (d, 2H, Ar-H), 8.07 (t, 1H, Ar-H), 8.43 (d, 2H, Ar-H), 8.79 (s, 1H, Ar-H).^13^C NMR (100 MHz, DMSO-d_6_) δ (ppm): 96.5, 122.9, 127.7, 129.1, 130.6, 131.0, 149.3, 169.7, and 186.1. MS (*m*/*z*): 306 (M^+^, 14.51%). Anal. Calcd for C_10_H_10_N_8_O_2_S (306): C, 39.22; H, 3.27; N, 36.60; S, 10.46. Found: C, 39.37; H, 3.30; N, 36.49; S, 10.33.

##### 3-(3,5-Diamino-4-((3-nitrophenyl)diazenyl)-1*H*-pyrazol-1-yl)-3-oxopropanenitrile (**6**)

Brown crystals from butanol; m.p. 262–264 °C. IR (cm^−1^) ʋ: 3485, 3360, 3202, 3132 (NH_2_), 2216 (CN), 1690 (C=O), 1620 (C=N), 1550 (C=C), 1510 (N=N), 1356(N-O). ^1^H-NMR (300 MHz, DMSO-*d_6_*) δ (ppm): 3.61 (s, 2H, CH_2_CN), 7.08 (s, 2H, NH_2_, D_2_O-exhangeable), 7.73 (t, 1H, Ar-H), 8.13 (d, 1H, Ar-H), 8.18 (s, 2H, NH_2_, D_2_O-exhangeable), 8.47 (d, 1H, Ar-H), 8.85 (s, 1H, Ar-H).^13^C NMR (100 MHz, DMSO-d_6_) δ (ppm): 27.8, 113.4, 116.0, 117.0, 127.7, 129.1, 130.7, 149.2, 149.4, 153.6, 154.7, and 161.4. MS (*m*/*z*): 314 (M^+^, 11.67%). Anal. Calcd for C_12_H_10_N_8_O_3_ (314): C, 45.85; H, 3.18; N, 35.67. Found: C, 45.92; H, 2.97; N, 35.59.

##### (3,5-Diamino-4-((3-nitrophenyl)diazenyl)-1*H*-pyrazol-1-yl)(phenyl) methanone (**7**)

Brown crystals from methanol; m.p. > 300 °C. IR (cm^−1^) ʋ: 3440, 3347, 3189, 3071 (NH_2_), 1670 (C=O), 16010 (C=N), 1603 (C=C), 1536 (N=N), 1350 (N-O).^1^H-NMR (300 MHz, DMSO-*d_6_*) δ (ppm): 7.07 (d, 1H, Ar-H), 7.73 (t, 1H, Ar-H), 8.10 (d, 1H, Ar-H), 8.14 (t, 1H, Ar-H), 8.25 (s, 2H, NH_2_, D_2_O-exchangeable), 8.46 (d, 1H, Ar-H), 8.47 (d, 1H, Ar-H), 8.52 (d, 2H, Ar-H), 8.84 (s, 1H, Ar-H), 9.51 (s, 2H, NH_2_, D_2_O-exchangeable). ^13^C NMR (100 MHz, DMSO-d_6_) δ (ppm): 109.4, 113.4, 116.9, 117.5, 121.8, 123.0, 127.7, 129.0, 130.7, 131.0, 149.2, 149.4, 153.6, 154.6 and 177.8. MS (*m*/*z*): 351 (M^+^,61.83%). Anal. Calcd for C_16_H_13_N_7_O_3_ (351): C, 54.70; H, 3.70; N, 27.92. Found: C, 54.75; H, 3.62; N, 27.87.

##### 4,6-Diamino-5-((3-nitrophenyl)diazenyl)pyrimidin-2(5*H*)-one (**8**)

Brown crystals from acetic acid; m.p. > 300 °C. IR (cm^−1^) ʋ:3400–3072 (broad, NH_2_), 1664 (C=O), 1611 (C=N), 1535 (N=N), 1382 (N-O).^1^H-NMR (300 MHz, DMSO-d_6_) δ (ppm): 3.47 (s, 1H, CH-N=N), 7.18 (broad, 4H, 2NH_2_, D_2_O-exhangeable), 7.65 (t, 1H, Ar-H), 8.19 (d, 1H, Ar-H), 8.84 (d, H, Ar-H), 8.21 (s, 1H, Ar-H).^13^C NMR (100 MHz, DMSO-d_6_) δ (ppm):86.4, 109.7, 111.3, 114.1, 120.1, 122.5, 131.4, 142.7, 148.8, and 163.9. MS (*m*/*z*): 275 (M^+^, 14.57%). Anal. Calcd for C_10_H_9_N_7_O_3_ (275): C, 43.64; H, 3.27; N, 35.64. Found: C, 43.59; H, 3.09; N, 35.78.

##### 4,6-Diamino-5-((3-nitrophenyl)diazenyl)pyrimidine-2(5*H*)-thione (**9**)

Reddish-brown crystals from methanol; m.p. 246–248 °C. IR (cm^−1^) ʋ: 3379, 3276, 3186, 3129 (NH_2_), 1601 (C=N), 1537 (N=N), 1351 (N-O), 1262 (C=S). ^1^H-NMR (300 MHz, DMSO-d_6_) δ (ppm): 3.42 (s, 1H, CH-N=N), 6.88–7.22 (broad, 4H, 2NH_2_, D_2_O-exhangeable), 7.65 (t, 1H, Ar-H), 8.22 (d, 1H, Ar-H), 8.82 (d, H, Ar-H), 8.21 (s, 1H, Ar-H). ^13^C NMR (100 MHz, DMSO-d_6_) δ (ppm): 87.3, 109.8, 111.3, 114.1, 120.1, 122.5, 131.4, 142.8, 148.7, and 184.3. MS (*m*/*z*): 291 (M^+^, 10.07%). Anal. Calcd for C_10_H_9_N_7_O_2_S (291): C, 41.24; H, 3.09; N, 33.68; S, 10.99. Found: C, 41.30; H, 3.12; N, 33.79; S, 11.08.

##### 2-Imino-5-((3-nitrophenyl)diazenyl)-2,5-dihydropyrimidine-4,6-diamine (**10**)

Orange crystals from methanol; m.p. > 300 °C. IR (cm^−1^) ʋ:3423, 3302, 3263 (NH_2_), 3093 (NH), 1622 (C=N), 1534 (N=N), 1345 (N-O). ^1^H NMR (300 MHz, DMSO-*d_6_*) δ (ppm): 3.46 (s, 1H, CH-N=N), 7.16 (broad, 4H, 2NH_2_, D_2_O-exhangeable), 7.65 (t, 1H, Ar-H), 8.20 (d, 1H, Ar-H), 8.84 (d, 1H, Ar-H), 8.21 (s, 1H, Ar-H), 13.46 (broad, 1H, NH, D_2_O-exhangeable).^13^C NMR (100 MHz, DMSO-d_6_) δ (ppm):87.3, 109.8, 111.2, 114.0, 120.1, 122.5, 131.3, 142.7, 148.7, and 161.4. MS (*m*/*z*): 274 (M^+^, 30.09%). Anal. Calcd for C_10_H_10_N_8_O_2_ (274): C, 43.80; H, 3.65; N, 40.88. Found: C, 43.68; H, 3.44; N, 40.92.

##### 6-((3-Nitrophenyl)diazenyl)-[1,2,4]triazolo[4,3-a]pyrimidine-5,7-diamine (**11**)

Orange crystals from acetone; m.p. > 300 °C. IR (cm^−1^) ʋ: 3472, 3429, 3318, 3201 (NH_2_), 1610 (C=N), 1522 (N=N), 1349 (N-O). ^1^H-NMR (300 MHz, DMSO-*d_6_*) δ (ppm): 6.04 (broad, 2H, NH_2_, D_2_O-exhangeable), 7.57 (s, 1H, Ar-H), 7.63 (t, 1H, Ar-H), 7.89 (d, 1H, Ar-H), 8.04 (s, 1H, Ar-H), 8.14 (d, 1H, Ar-H), 8.60 (broad, 2H, NH_2_, D_2_O-exhangeable). ^13^C NMR (100 MHz, DMSO-d_6_) δ (ppm): 108.7, 113.5, 117.8, 123.5, 127.7, 130.8, 137.7, 140.4, 149.3, 153.3, and 155.9. MS (*m*/*z*): 299 (M^+^, 13.60%). Anal. Calcd for C_11_H_9_N_9_O_2_ (299): C, 44.15; H, 3.01; N, 42.14. Found: C, 44.09; H, 2.94; N, 41.99.

##### 6,8-Diamino-7-((3-nitrophenyl)diazenyl)pyrimido[1,2-a]purin-10(3*H*)-one (**12**)

Brown crystals from acetone; m.p. > 300 °C. IR (cm^−1^) ʋ: 3319–2691 (broad, NH_2_), 1697 (C=O), 1621 (C=N), 1610 (C=C), 1550 (N=N), 1372 (N-O). ^1^H-NMR (300 MHz, DMSO-*d_6_*) δ (ppm): 6.52 (s, 2H, NH_2_, D_2_O-exhangeable), 7.56 (s, 2H, NH_2_, D_2_O-exhangeable), 7.61 (t, 1H, Ar-H), 7.89 (d, 1H, Ar-H), 8.04 (s, 1H, Ar-H), 8.14 (d, 1H, Ar-H), 8.43 (s, 1H, Ar-H), 11.98 (s, 1H, NH, D_2_O-exhangeable). ^13^C NMR (100 MHz, DMSO-d_6_) δ (ppm): 110.7, 110.9, 111.5, 118.5, 122.3, 131.0, 138.4, 143.9, 149.1, 156.0, 156.3, 156.9, and 162.7. MS (*m*/*z*): 366 (M^+^, 22.31%). Anal. Calcd for C_14_H_10_N_10_O_3_ (366): C, 45.90; H, 2.73; N, 38.25. Found: C, 46.01; H, 2.64; N, 38.31.

### 3.2. Biological Evaluation

#### 3.2.1. MTT Assay for Cell Viability

To investigate the effect of the newly synthesized compounds on breast cancer cells, an MTT assay [44,45,63] was performed against MCF-7 and MDA-MB-231 cell lines as representative of cancerous cells and MCF-10 for the normal ones (see Supplementary S1).

#### 3.2.2. Hsp90 Inhibitory Assay

The Hsp90 assay was performed using the established method using the Human Hsp90 Sandwich ELISA Kit [47] (Catalog Number: KE00054, sensitivity: 90 pg/mL) for selected synthetic compounds **9** and **10**. Details are summarized in Supplementary S1.

#### 3.2.3. Activation of Caspases

For a deep and systematic investigation of cell apoptosis, the effect of compounds **9** and **10** on caspases 3 and 8 was evaluated and compared to positive and passive controls; details are summarized in Supplementary S1.

#### 3.2.4. Effects on BAX and Bcl-2 Proteins

The activities of compounds **9** and **10** against Bcl2 and BAX using the MDA-MB-231 cell line and normal MCF-10 as the negative control were investigated according to the literature. See Supplementary S1.

#### 3.2.5. Cyclin-D1 Inhibitory Assay

A cyclin-D1 assay was performed by the established reported method using G1/S-specific cyclin-D1 (CCND1) (Human) ELISA Kit (Catalog Number: Catalog # E4287-100) for selected synthetic compounds **9** and **10**. Details are summarized in Supplementary S1.

#### 3.2.6. Molecular Modeling

##### Molecular Docking Study

The docking studies were performed on compound **9** using the co-crystallized ligand of crystal protein (PDB code: 2XDX) obtained from the RCSB to generate the binding sites. Molecular docking was performed using docking algorithms of Autodock Vina 4.0. The docking scores of the best-fitted poses with the active site of the Hsp90 were recorded, and the 3D orientation was generated using Discovery Studio 2016 visualizer software; for more details, see Supplementary S1.

##### Molecular Dynamics (MD) Simulation Study

The Desmond simulation package from Schrödinger LLC was utilized to conduct molecular dynamics (MD) simulations for compound **9**. Details are shown in Supplementary S1.

## 4. Conclusions

Our current research aims to make advancements in the treatment of breast cancer by developing and producing new compounds **2**–**12** based on pyrazole/pyrimidine that act as inhibitors of Hsp90. Compounds **9** and **10** have been identified as the most potent anti-breast cancer agents, exhibiting a substantial inhibition of Hsp90. They demonstrated promising IC_50_ values against the tested cell lines. Compounds **9** and **10** exhibited no impact on non-tumor cells MCF-10A, indicating the selective targeting of these compounds towards tumor cells. The apoptotic studies demonstrate that compounds **9** and **10** can enhance the expression of proapoptotic markers (caspase 3, caspase 8, and BAX) while inhibiting the expression of the anti-apoptotic protein BCL-2. Compound **9** demonstrated favorable binding affinities with the active sites of Hsp90, as shown by molecular docking and simulation assays. Based on the data, **9** and **10** are promising Hsp90 inhibitor candidates for advancing innovative breast cancer treatments.

## Figures and Tables

**Table 1:** Comparative study between conventional, grinding, and microwave techniques.

Cpd. No.	Time “min”			Yield %			YE			RME			OE			AE
	Th.	G.	M.W.	Th.	G.	M.W.	Th.	G.	M.W.	Th.	G.	M.W.	Th.	G.	M.W.	
**2**	120	10	1	49	79	92	0.4083	7.9	92.00	0.4289	0.6915	0.8053	0.4289	0.6915	0.8053	100
**3**	300	12	2	52	78	94	0.1733	6.5	47.00	0.4679	0.7018	0.8458	0.4679	0.7018	0.8458	100
**4**	480	13	4	51	79	92	0.1063	6.08	23.00	0.3976	0.6159	0.7172	0.4469	0.6923	0.8062	88.96
**5**	600	16	5	48	77	95	0.0800	4.81	19.00	0.4173	0.6694	0.8256	0.4173	0.6694	0.8256	100
**6**	720	14	5	53	77	91	0.0736	5.50	18.20	0.4623	0.6716	0.7937	0.4623	0.6716	0.7937	100
**7**	960	18	5	53	79	95	0.0552	4.39	19.00	0.4686	0.6984	0.8399	0.4686	0.6984	0.8399	100
**8**	240	12	2	51	80	91	0.2125	6.67	45.50	0.4369	0.6854	0.7796	0.4369	0.6854	0.7796	100
**9**	300	15	3	50	78	92	0.1667	5.20	30.67	0.4318	0.6735	0.7944	0.4318	0.6735	0.7944	100
**10**	300	14	3	52	76	94	0.1733	5.43	31.33	0.4002	0.5849	0.7234	0.4528	0.6617	0.8184	88.39
**11**	600	16	4	54	76	92	0.0900	4.75	23.00	0.4680	0.6587	0.7973	0.4680	0.6587	0.7973	100
**12**	720	16	4	49	78	90	0.0681	4.88	22.50	0.4353	0.6929	0.7995	0.4353	0.6929	0.7995	100

Th.: conventional reactions; G.: grinding reactions; M.W.: microwave; YE: yield economy; RME: maximum reaction efficiency; OE: optimum efficiency; AE: atomic economy.

**Table 2:**
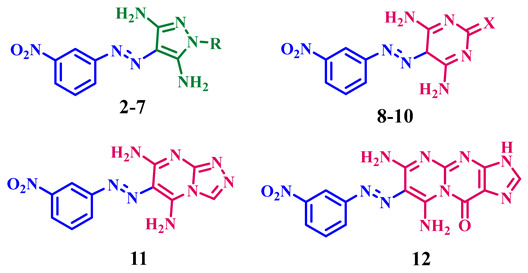
Antiproliferative action of targets **2**–**12** against MCF7 and MDAMB231 cell lines.

Compound	R	X	Antiproliferative Activity IC_50_ (µM)	
			MCF-7	MDA-MB231
**2**	Phenyl	NA	6.20 ± 0.40	46.40 ± 2.40
**3**	2,4-Dinitro phenyl	NA	247.30 ± 8.20	68.30 ± 3.40
**4**	CO-NH_2_	NA	236.40 ± 6.90	14.50 ± 1.10
**5**	CS-NH_2_	NA	85.90 ± 3.80	19.90 ± 1.30
**6**	CO-CH_2_CN	NA	234.50 ± 6.50	35.90 ± 2.10
**7**	CO-phenyl	NA	221.40 ± 6.40	72.50 ± 3.30
**8**	NA	O	33.60 ± 2.00	39.80 ± 2.50
**9**	NA	S	26.10 ± 1.30	4.70 ± 0.20
**10**	NA	NH	7.70 ± 0.50	10.70 ± 0.40
**11**	NA	NA	237.20 ± 7.00	25.80 ± 1.80
**12**	NA	NA	244.40 ± 8.00	57.30 ± 2.90
**Doxorubicin** [47]	--	--	33.20 ± 3.50	3.20 ± 0.10
**Cisplatin** [48]	--	--	3.70 ± 0.35	--

NA: Not applicable.

**Table 3:** The cytotoxic effect of compounds **9** and **10** against MCF-10A normal cell line.

**MCF-10A** **IC_50_ (µM)**	**Compound 9**	**Compound 10**
	30.70 ± 0.94	37.30 ± 1.40

**Table 4:** Hsp90 inhibitory activity of compounds **9** and **10** and novobiocin on MDA-MB231 cell line.

Compound	Hsp90 (IC_50_ = µM)
**9**	2.44 ± 0.08
**10**	7.30 ± 0.24
**Novobiocin**	1.14 ± 0.04

**Table 5:** Caspases-3 and 8 levels of **9** and **10** in breast cancer MDA-MB231 cell line.

	Normal Cell Line MCF-10A	Untreated MDA-MB231 Cell Line	Compound 9	Compound 10
**Caspase-3 ng/mL**	5.65 ± 0.95	2.40 ± 0.60	9.30 ± 0.40	8.20 ± 0.30
**Caspase-8 ng/mL**	5.30 ± 0.50	2.00 ± 0.33	7.60 ± 0.20	8.50 ± 0.20

**Table 6:** Bax and Bcl-2 levels for **9** and **10** on cancerous MDA-MB231 cell line.

	Normal MCF-10A Cell Line	Untreated MDA-MB231 Cell Line	Compound 9	Compound 10
**Bcl2 ng/mL**	5.50 ± 0.50	28.60 ± 0.40	21.46 ± 0.60	15.10 ± 0.85
**BAX ng/mL**	8.95 ± 0.30	2.10 ± 0.20	7.50 ± 0.40	6.30 ± 0.90

**Table 7:** CD1 inhibitory activity of compounds **9** and **10** and controls.

	MCF-10A	MDA-MB231	Compound 9	Compound 10
**CD1 ng/mL**	7.571 ± 1.10	26.30 ± 1.50	11.95 ± 1.30	15.15 ± 2.40

**Table 8:** (∆G) kcal/mol of compound **9** against (HSP90) target site PDB ID: 2XDX.

Ligand	RMSD Value (Å)	Docking Score (kcal/mol)	Interactions	
			H.B	Pi-Interactions
**Compound 9**	1.41	−6.45	3	5
**Co-crystalized ligand**	1.02	−6.75	2	8

## Data Availability

The original contributions presented in the study are included in the article/Supplementary Materials, further inquiries can be directed to the corresponding authors.

## Supplementary Materials

The following supporting information can be downloaded at: https://www.mdpi.com/article/10.3390/ph17101284/s1.

## References

[1] Afifi A.M., Saad A.M., Al-Husseini M.J., Elmehrath A.O., Northfelt D.W., Sonbol M.B. (2020). Causes of death after breast cancer diagnosis: A us population-based analysis. Cancer.

[2] Ahmad A. (2019). Breast cancer statistics: Recent trends. Breast Cancer Metastasis and Drug Resistance: Challenges and Progress.

[3] Arnold M., Morgan E., Rumgay H., Mafra A., Singh D., Laversanne M., Vignat J., Gralow J.R., Cardoso F., Siesling S. (2022). Current and future burden of breast cancer: Global statistics for 2020 and 2040. Breast.

[4] Sung H., Ferlay J., Siegel R.L., Laversanne M., Soerjomataram I., Jemal A., Bray F. (2021). Global cancer statistics 2020: Globocan estimates of incidence and mortality worldwide for 36 cancers in 185 countries. CA A Cancer J. Clin..

[5] Ngumi M.W. (2023). Experiences of Women with Breast Cancer during the COVID-19 Pandemic in Nairobi, Kenya. Ph.D. Thesis.

[6] Lakkis N.A., Abdallah R.M., Musharrafieh U.M., Issa H.G., Osman M.H. (2024). Epidemiology of breast, corpus uteri, and ovarian cancers in lebanon with emphasis on breast cancer incidence trends and risk factors compared to regional and global rates. Cancer Control.

[7] Zhu J.W., Charkhchi P., Adekunte S., Akbari M.R. (2023). What is known about breast cancer in young women?. Cancers.

[8] Hoter A., El-Sabban M.E., Naim H.Y. (2018). The Hsp90 family: Structure, regulation, function, and implications in health and disease. Int. J. Mol. Sci..

[9] Sykut M.R.H. (2021). Heat Shock Protein: Potential Approach for Chemotherapy. Ph.D. Thesis.

[10] Zhang M., Bi X. (2024). Heat shock proteins and breast cancer. Int. J. Mol. Sci..

[11] Barrott J.J., Haystead T.A. (2013). Hsp90, an unlikely ally in the war on cancer. FEBS J..

[12] Birbo B., Madu E.E., Madu C.O., Jain A., Lu Y. (2021). Role of Hsp90 in cancer. Int. J. Mol. Sci..

[13] Maloney A., Workman P. (2002). Hsp90 as a new therapeutic target for cancer therapy: The story unfolds. Expert Opin. Biol. Ther..

[14] Whitesell L., Lin N.U. (2012). Hsp90 as a platform for the assembly of more effective cancer chemotherapy. Biochim. Biophys. Acta (BBA)-Mol. Cell Res..

[15] Kerru N., Gummidi L., Maddila S., Gangu K.K., Jonnalagadda S.B. (2020). A review on recent advances in nitrogen-containing molecules and their biological applications. Molecules.

[16] Tsygankova V., Ya A., Shtompel O., Romaniuk O., Yaikova M., Hurenko A., Solomyanny R., Abdurakhmanova E., Klyuchko S., Holovchenko O. (2017). Application of synthetic low molecular weight heterocyclic compounds derivatives of pyrimidine, pyrazole and oxazole in agricultural biotechnology as a new plant growth regulating substances. Int. J. Med. Biotechnol. Genet..

[17] Solomyanny R., Mrug G., Frasinyuk M., Shablykin O., Brovarets V. (2016). Study of auxin, cytokinin and gibberellin-like activity of heterocyclic compounds derivatives of pyrimidine, pyridine, pyrazole and isoflavones. Eur. J. Biotechnol. Biosci..

[18] Loto R., Loto C., Popoola A., Ranyaoa M. (2012). Pyrimidine derivatives as environmentally-friendly corrosion inhibitors: A review. Int. J. Phys. Sci..

[19] Tsygankova V., Voloshchuk I., Klyuchko S., Pilyo S., Brovarets V., Kovalenko O. (2022). The effect of pyrimidine and pyridine derivatives on the growth and productivity of sorghum. Int. J. Bot. Stud.

[20] Choudhary D., Garg S., Kaur M., Sohal H.S., Malhi D.S., Kaur L., Verma M., Sharma A., Mutreja V. (2023). Advances in the synthesis and bio-applications of pyrazine derivatives: A review. Polycycl. Aromat. Compd..

[21] Kumar R., Sharma R., Sharma D.K. (2023). Pyrazole; a privileged scaffold of medicinal chemistry: A comprehensive review. Curr. Top. Med. Chem..

[22] Othman I.M., Alamshany Z.M., Tashkandi N.Y., Gad-Elkareem M.A., Anwar M.M., Nossier E.S. (2021). New pyrimidine and pyrazole-based compounds as potential egfr inhibitors: Synthesis, anticancer, antimicrobial evaluation and computational studies. Bioorg. Chem..

[23] Abdellatif K.R., Bakr R.B. (2021). Pyrimidine and fused pyrimidine derivatives as promising protein kinase inhibitors for cancer treatment. Med. Chem. Res..

[24] Nassar I.F., Atta-Allah S.R., Elgazwy A.-S.S.H. (2015). A convenient synthesis and molecular modeling study of novel pyrazolo [3, 4-d] pyrimidine and pyrazole derivatives as anti-tumor agents. J. Enzym. Inhib. Med. Chem..

[25] El-Gamal M.I., Zaraei S.-O., Madkour M.M., Anbar H.S. (2022). Evaluation of substituted pyrazole-based kinase inhibitors in one decade (2011–2020): Current status and future prospects. Molecules.

[26] Ardevines S., Marques-Lopez E., Herrera R.P. (2023). Heterocycles in breast cancer treatment: The use of pyrazole derivatives. Curr. Med. Chem..

[27] Amewu R.K., Sakyi P.O., Osei-Safo D., Addae-Mensah I. (2021). Synthetic and naturally occurring heterocyclic anticancer compounds with multiple biological targets. Molecules.

[28] Harrison C., Kiladjian J.-J., Al-Ali H.K., Gisslinger H., Waltzman R., Stalbovskaya V., McQuitty M., Hunter D.S., Levy R., Knoops L. (2012). Jak inhibition with ruxolitinib versus best available therapy for myelofibrosis. N. Engl. J. Med..

[29] Longley D.B., Harkin D.P., Johnston P.G. (2003). 5-fluorouracil: Mechanisms of action and clinical strategies. Nat. Rev. Cancer.

[30] Kverka M., Rossmann P., Tlaskalova-Hogenova H., Klimesova K., Jharap B., de Boer N.K., Vos R.M., van Bodegraven A.A., Lukas M., Mulder C.J. (2011). Safety and efficacy of the immunosuppressive agent 6-tioguanine in murine model of acute and chronic colitis. BMC Gastroenterol..

[31] Mohamady S., Ismail M.I., Mogheith S.M., Attia Y.M., Taylor S.D. (2020). Discovery of 5-aryl-3-thiophen-2-yl-1h-pyrazoles as a new class of Hsp90 inhibitors in hepatocellular carcinoma. Bioorg. Chem..

[32] Zajec Ž., Dernovšek J., Distel M., Gobec M., Tomašič T. (2023). Optimisation of pyrazolo [1,5-a] pyrimidin-7 (4h)-one derivatives as novel Hsp90 c-terminal domain inhibitors against ewing sarcoma. Bioorg. Chem..

[33] Al-Wahaibi L.H., Abou-Zied H.A., Beshr E.A., Youssif B.G., Hayallah A.M., Abdel-Aziz M. (2023). Design, synthesis, antiproliferative actions, and dft studies of new bis–pyrazoline derivatives as dual egfr/brafv600e inhibitors. Int. J. Mol. Sci..

[34] Al-Wahaibi L.H., Abou-Zied H.A., Hisham M., Beshr E.A., Youssif B.G., Bräse S., Hayallah A.M., Abdel-Aziz M. (2023). Design, synthesis, and biological evaluation of novel 3-cyanopyridone/pyrazoline hybrids as potential apoptotic antiproliferative agents targeting egfr/brafv600e inhibitory pathways. Molecules.

[35] Al-Wahaibi L.H., Gouda A.M., Abou-Ghadir O.F., Salem O.I., Ali A.T., Farghaly H.S., Abdelrahman M.H., Trembleau L., Abdu-Allah H.H., Youssif B.G. (2020). Design and synthesis of novel 2,3-dihydropyrazino [1,2-a] indole-1,4-dione derivatives as antiproliferative egfr and brafv600e dual inhibitors. Bioorg. Chem..

[36] Al-Wahaibi L.H., Mahmoud M.A., Mostafa Y.A., Raslan A.E., Youssif B.G. (2023). Novel piperine-carboximidamide hybrids: Design, synthesis, and antiproliferative activity via a multi-targeted inhibitory pathway. J. Enzym. Inhib. Med. Chem..

[37] Elbastawesy M.A., Aly A.A., Ramadan M., Elshaier Y.A., Youssif B.G., Brown A.B., Abuo-Rahma G.E.-D.A. (2019). Novel pyrazoloquinolin-2-ones: Design, synthesis, docking studies, and biological evaluation as antiproliferative egfr-tk inhibitors. Bioorg. Chem..

[38] Mohamed M.F., Youssif B.G., Shaykoon M.S.A., Abdelrahman M.H., Elsadek B.E., Aboraia A.S., Abuo-Rahma G.E.-D.A. (2019). Utilization of tetrahydrobenzo [4, 5] thieno [2, 3-d] pyrimidinone as a cap moiety in design of novel histone deacetylase inhibitors. Bioorg. Chem..

[39] Essa B.M., Selim A.A., Sayed G.H., Anwer K.E. (2022). Conventional and microwave-assisted synthesis, anticancer evaluation, 99mtc-coupling and in-vivo study of some novel pyrazolone derivatives. Bioorg. Chem..

[40] Anwer K.E., Sayed G.H., Ramadan R.M. (2022). Synthesis, spectroscopic, dft calculations, biological activities and molecular docking studies of new isoxazolone, pyrazolone, triazine, triazole and amide derivatives. J. Mol. Struct..

[41] Husseiny E.M., Abulkhair H.S., El-Dydamony N.M., Anwer K.E. (2023). Exploring the cytotoxic effect and cdk-9 inhibition potential of novel sulfaguanidine-based azopyrazolidine-3, 5-diones and 3, 5-diaminoazopyrazoles. Bioorg. Chem..

[42] Anwer K.E., Hamza Z.K., Ramadan R.M. (2023). Synthesis, spectroscopic, dft calculations, biological activity, sar, and molecular docking studies of novel bioactive pyridine derivatives. Sci. Rep..

[43] Naguib H., Dauoud N., Shaban S., Abdelghaffar N., Sayed G., Anwer K. (2022). Synthesis of pyrazolone derivatives by grinding, microwave, and conventional techniques and their antimicrobial activity. Russ. J. Org. Chem..

[44] Mahmoud M.A., Mohammed A.F., Salem O.I., Rabea S.M., Youssif B.G. (2023). Design, synthesis, and antiproliferative properties of new 1, 2, 3-triazole-carboximidamide derivatives as dual egfr/vegfr-2 inhibitors. J. Mol. Struct..

[45] El-Sheref E.M., Elbastawesy M.A., Brown A.B., Shawky A.M., Gomaa H.A., Bräse S., Youssif B.G. (2021). Design and synthesis of (2-oxo-1,2-dihydroquinolin-4-yl)-1,2,3-triazole derivatives via click reaction: Potential apoptotic antiproliferative agents. Molecules.

[46] Youssif B.G., Abdelrahman M.H., Abdelazeem A.H., Ibrahim H.M., Salem O.I., Mohamed M.F., Treambleau L., Bukhari S.N.A. (2018). Design, synthesis, mechanistic and histopathological studies of small-molecules of novel indole-2-carboxamides and pyrazino [1,2-a] indol-1(2h)-ones as potential anticancer agents effecting the reactive oxygen species production. Eur. J. Med. Chem..

[47] Alosaimy A.M., Abouzied A.S., Alsaedi A.M., Alafnan A., Alamri A., Alamri M.A., Break M.K.B., Sabour R., Farghaly T.A. (2023). Discovery of novel indene-based hybrids as breast cancer inhibitors targeting Hsp90: Synthesis, bio-evaluation and molecular docking study. Arab. J. Chem..

[48] Mahmoud H.K., Abdelhady H.A., Elaasser M.M., Hassain D.Z.H., Gomha S.M. (2021). Microwave-assisted one-pot three component synthesis of some thiazolyl (hydrazonoethyl)thiazoles as potential anti-breast cancer agents. Polycycl. Aromat. Compd..

[49] Kusuma B.R., Khandelwal A., Gu W., Brown D., Liu W., Vielhauer G., Holzbeierlein J., Blagg B.S.J. (2014). Synthesis and biological evaluation of coumarin replacements of novobiocin as Hsp90 inhibitors. Bioorg. Med. Chem..

[50] Fan T.-J., Han L.-H., Cong R.-S., Liang J. (2005). Caspase family proteases and apoptosis. Acta Biochim. Biophys. Sin..

[51] Abou-Zied H.A., Youssif B.G., Mohamed M.F., Hayallah A.M., Abdel-Aziz M. (2019). Egfr inhibitors and apoptotic inducers: Design, synthesis, anticancer activity and docking studies of novel xanthine derivatives carrying chalcone moiety as hybrid molecules. Bioorg. Chem..

[52] Youssif B.G., Mohamed A.M., Osman E.E.A., Abou-Ghadir O.F., Elnaggar D.H., Abdelrahman M.H., Treamblu L., Gomaa H.A. (2019). 5-chlorobenzofuran-2-carboxamides: From allosteric cb1 modulators to potential apoptotic antitumor agents. Eur. J. Med. Chem..

[53] Cory S., Adams J.M. (2002). The bcl2 family: Regulators of the cellular life-or-death switch. Nat. Rev. Cancer.

[54] Khan Z., Bisen P.S. (2013). Oncoapoptotic signaling and deregulated target genes in cancers: Special reference to oral cancer. Biochim. Biophys. Acta (BBA)-Rev. Cancer.

[55] Wang Z. (2021). Regulation of cell cycle progression by growth factor-induced cell signaling. Cells.

[56] Choi Y.J., Anders L. (2014). Signaling through cyclin d-dependent kinases. Oncogene.

[57] Giacinti C., Giordano A. (2006). Rb and cell cycle progression. Oncogene.

[58] Gitig D.M. (2001). Molecular Interactions between p27 (Kip1) and p21 (Cip1) and G1 Cyclin/Cdk Complexes. Ph.D. Thesis.

[59] Coqueret O. (2002). Linking cyclins to transcriptional control. Gene.

[60] Mey A.S., Juárez-Jiménez J., Hennessy A., Michel J. (2016). Blinded predictions of binding modes and energies of Hsp90-α ligands for the 2015 d3r grand challenge. Bioorg. Med. Chem..

[61] Elbastawesy M.A., Mohamed F.A., Zaki I., Alahmdi M.I., Alzahrani S.S., Alzahrani H.A., Gomaa H.A., Youssif B.G. (2023). Design, synthesis and antimicrobial activity of novel quinoline-2-one hybrids as promising DNA gyrase and topoisomerase iv inhibitors. J. Mol. Struct..

[62] Ivanova L., Tammiku-Taul J., García-Sosa A.T., Sidorova Y., Saarma M., Karelson M. (2018). Molecular dynamics simulations of the interactions between glial cell line-derived neurotrophic factor family receptor gfrα1 and small-molecule ligands. ACS Omega.

[63] Frejat F.O.A., Zhai H., Cao Y., Wang L., Mostafa Y.A., Gomaa H.A., Youssif B.G., Wu C. (2022). Novel indazole derivatives as potent apoptotic antiproliferative agents by multi-targeted mechanism: Synthesis and biological evaluation. Bioorg. Chem..

