# Supplementary material for: New Pyrazole/Pyrimidine-Based Scaffolds as Inhibitors of Heat Shock Protein 90 Endowed with Apoptotic Anti-Breast Cancer Activity
Source: Pharmaceuticals (Basel). 2024 Sep 27;17(10):1284. doi: 10.3390/ph17101284 (PMC11510237; doi:10.3390/ph17101284)

Supplementary File

Contents:

Cover Page	2
General details	3
Spectral Data	4
Biological evaluation methods	52

New Pyrazole/Pyrimidine-Based Scaffolds as Inhibitors of Heat Shock Protein 90 Endowed with Apoptotic Anti-Breast Cancer Activity

Lamya H. Al-Wahaibi [1], Mohammed A. I. Elbastawesy [2], Nader E. Abodya [3], Bahaa G. M. Youssif [4,*], Stefan Bräse [5,*], Sara N. Shabaan [6], Galal H. Sayed [7] and Kurls E. Anwer [7]

[1] Department of Chemistry, College of Sciences, Princess Nourah bint Abdulrahman University, Riyadh 11671, Saudi Arabia; lhalwahaibi@pnu.edu.sa
[2] Department of Pharmaceutical Organic Chemistry, Faculty of Pharmacy, Al-Azhar University, Assiut 71524, Egypt; mohamedali.pharm.ast@azhar.edu.eg
[3] Department of Pharmaceutical Chemistry, Faculty of Pharmacy, University of Tabuk, Tabuk 71491, Saudi Arabia; nabodya@ut.edu.sa
[4] Department of Pharmaceutical Organic Chemistry, Faculty of Pharmacy, Assiut University, Assiut 71526, Egypt
[5] Institute of Biological and Chemical Systems, IBCS-FMS, Karlsruhe Institute of Technology, 76131 Karlsruhe, Germany
[6] Department of Chemistry, Faculty of Science (Girls), Al-Azhar University, Nasr City, Cairo 11754, Egypt; saranabil.2259@azhar.edu.eg
[7] Heterocyclic Synthesis Lab., Chemistry Department, Faculty of Science, Ain Shams University, Abbassia, Cairo 11566, Egypt; galal.hosny.sayed@gmail.com (G.H.S.); kurlsekram@sci.asu.edu.eg (K.E.A.)
* Correspondence: bahaa.youssif@pharm.aun.edu.eg or bgyoussif2@gmail.com (B.G.M.Y.); braese@kit.edu (S.B.); Tel.: +20-(002)-01098294419 (B.G.M.Y.)

Spectra of compounds 2-12

Figure S1: IR spectrum of compound 2

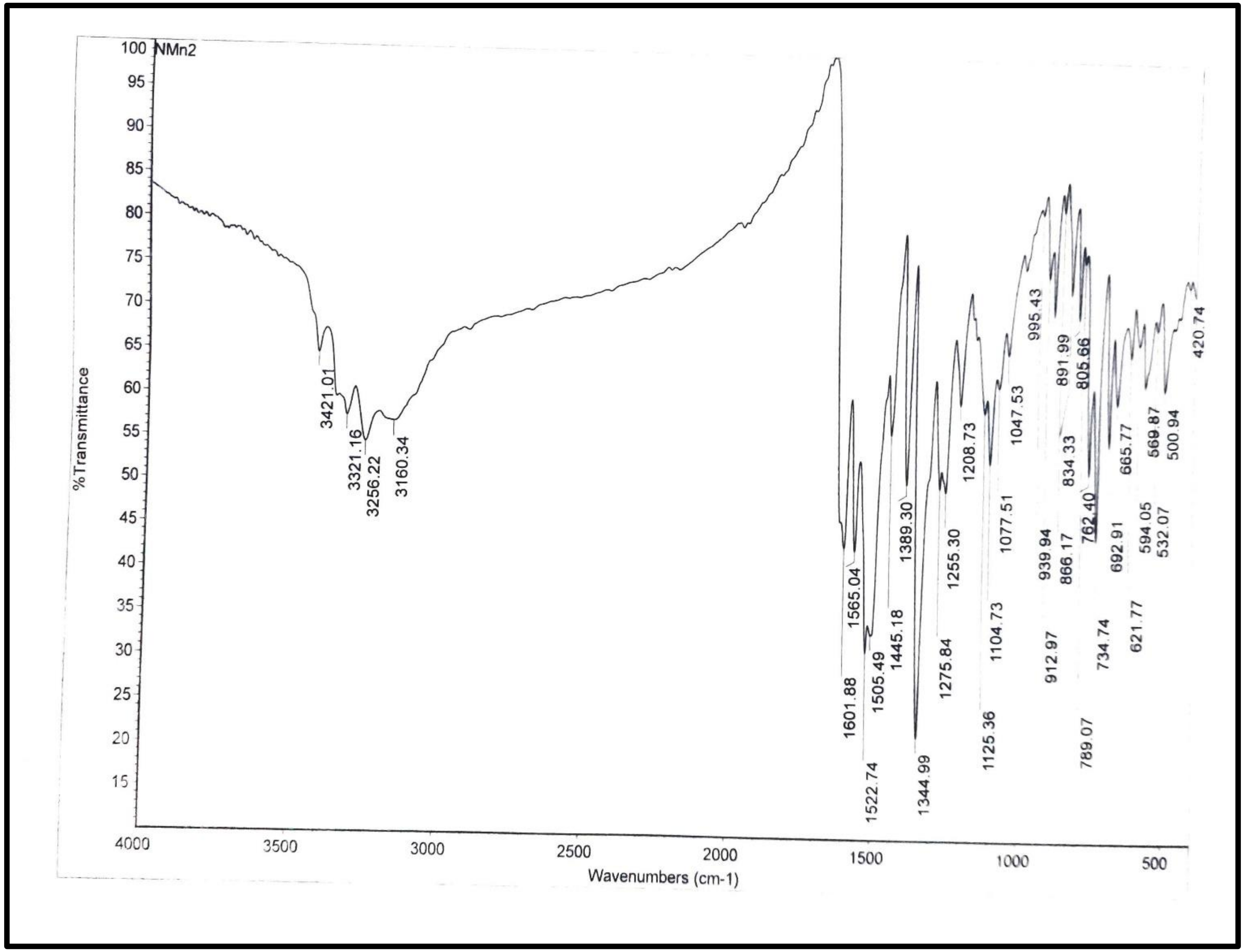

Figure S2: ^{1}H NMR spectrum of compound 2

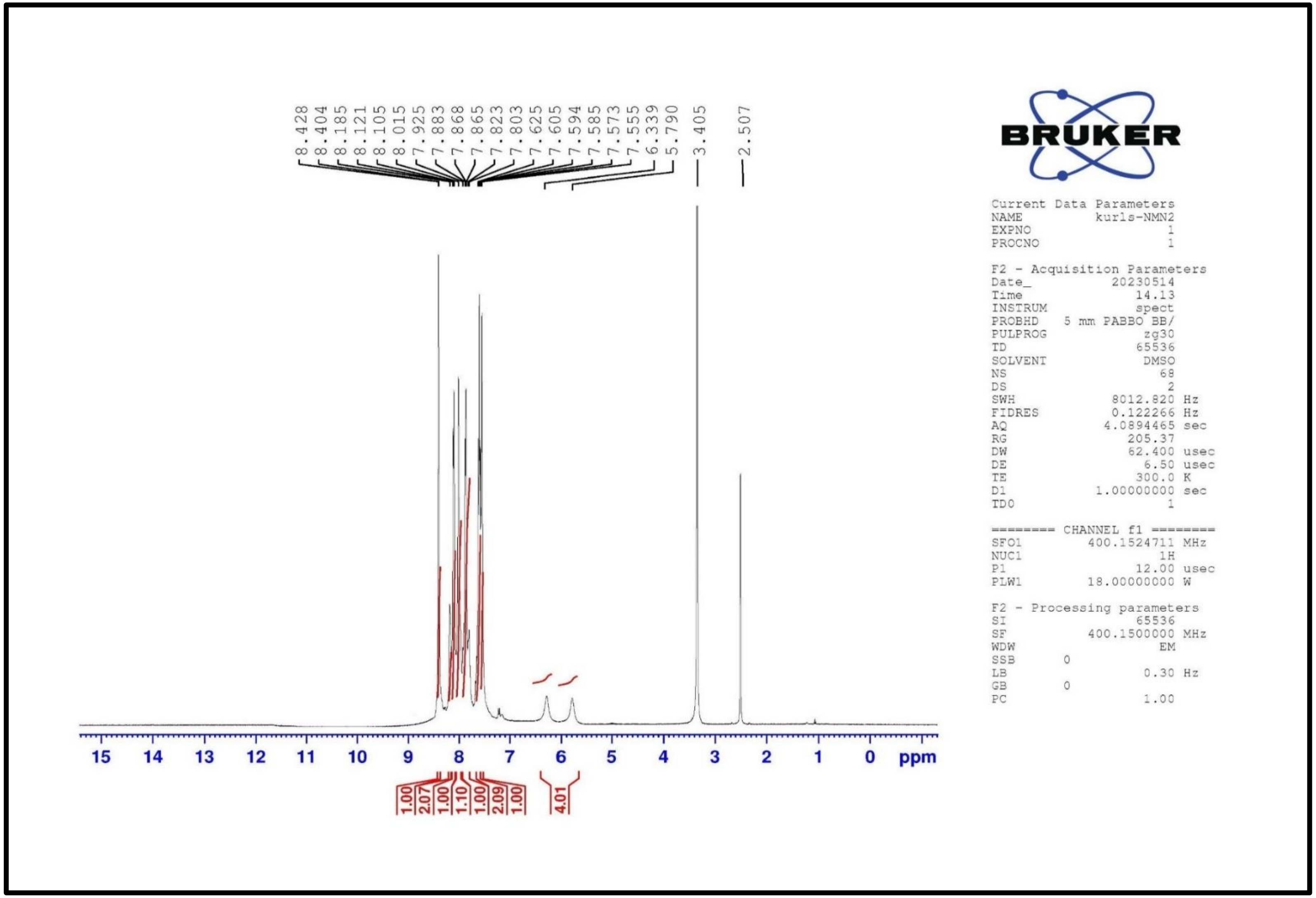

Figure S3: ^{13}C NMR spectrum of compound 2

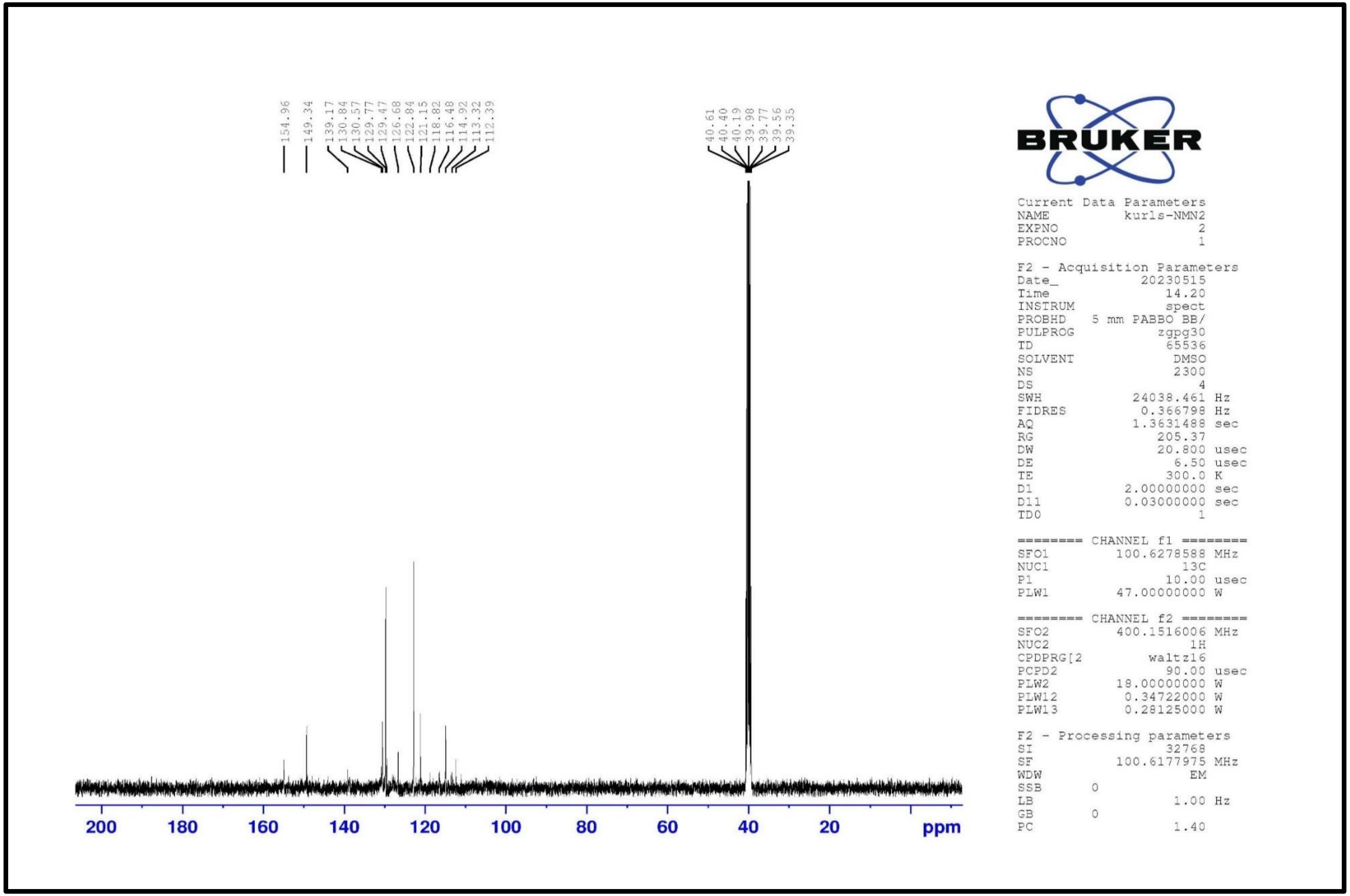
154.96
149.34
139.17
130.84
130.57
129.77
129.47
126.68
122.84
121.15
118.82
116.48
114.92
113.32
112.39
40.61
40.40
40.19
39.98
39.77
39.56
39.35
200
180
160
140
120
100
80
60
40
20
ppm
BRUKER
Current Data Parameters
NAME kurls-NMN2
EXPNO 2
PROCNO 1
F2 - Acquisition Parameters
Date_ 20230515
Time 14.20
INSTRUM spect
PROBHD 5 mm PABBO BB/
PULPROG zgpg30
TD 65536
SOLVENT DMSO
NS 2300
DS 4
SWH 24038.461 Hz
FIDRES 0.366798 Hz
AQ 1.3631488 sec
RG 205.37
DW 20.800 usec
DE 6.50 usec
TE 300.0 K
D1 2.00000000 sec
D11 0.03000000 sec
TD0 1
======== CHANNEL f1 ========
SFO1 100.6278588 MHz
NUC1 13C
P1 10.00 usec
PLW1 47.00000000 W
======== CHANNEL f2 ========
SFO2 400.1516006 MHz
NUC2 1H
CPDPRG[2 waltz16
PCPD2 90.00 usec
PLW2 18.00000000 W
PLW12 0.34722000 W
PLW13 0.28125000 W
F2 - Processing parameters
SI 32768
SF 100.6177975 MHz
WDW EM
SSB 0
LB 1.00 Hz
GB 0
PC 1.40

Figure S4: Mass spectrum of compound 2

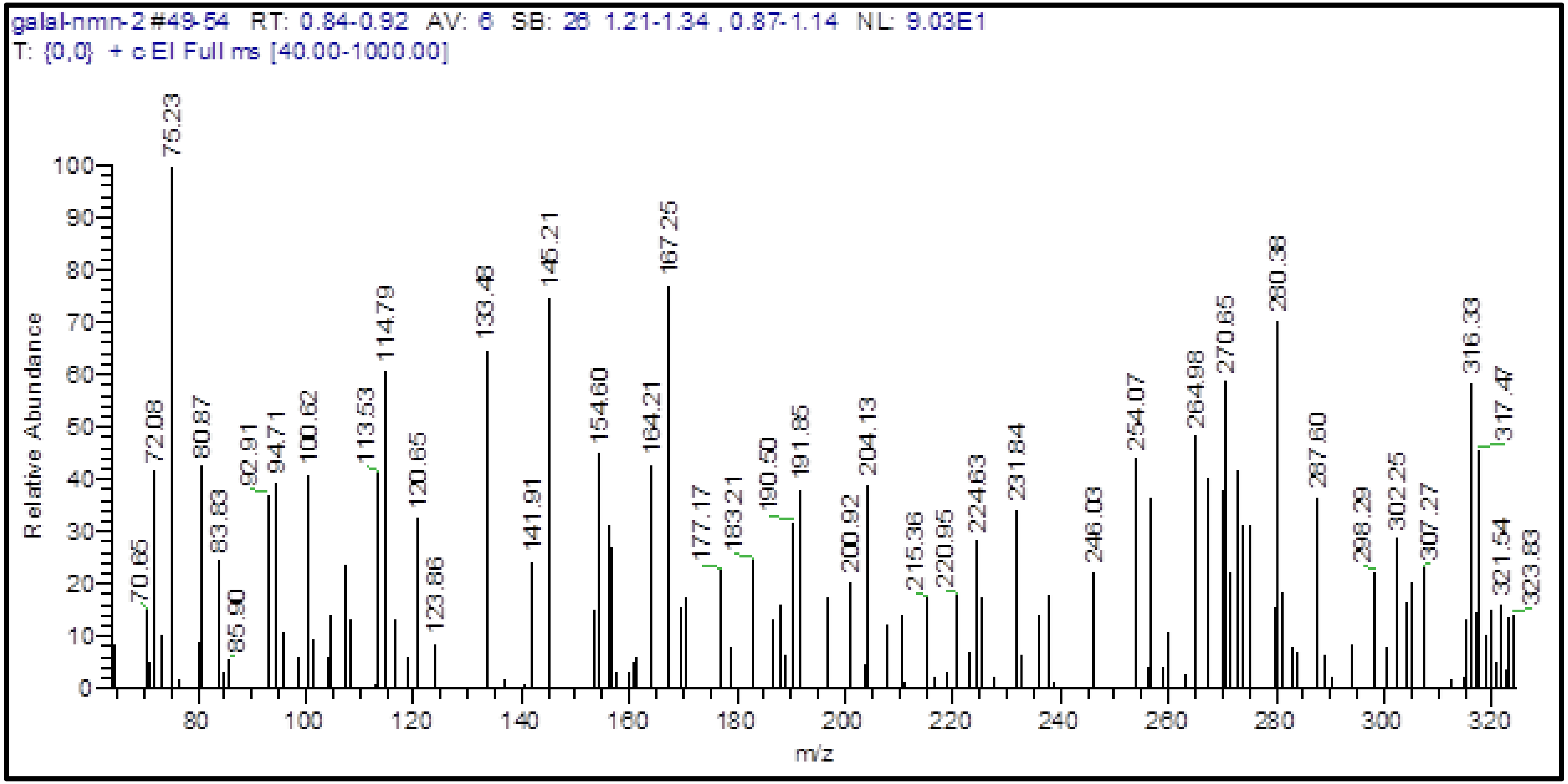

Figure S5: IR spectrum of compound 3

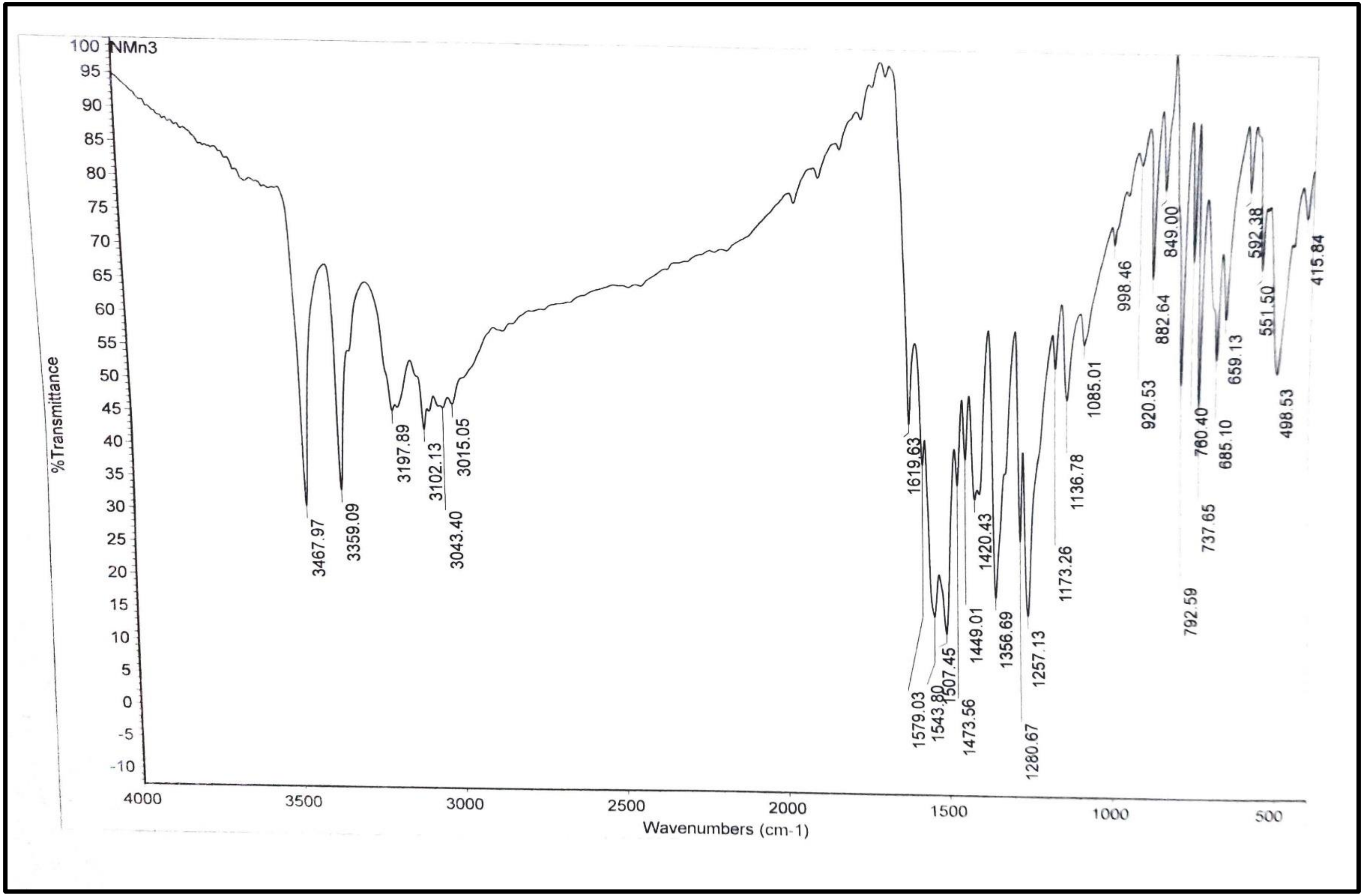
NMn3
%Transmittance
Wavenumbers (cm-1)
3467.97
3359.09
3197.89
3102.13
3043.40
3015.05
1619.63
1579.03
1543.80
1507.45
1473.56
1449.01
1420.43
1356.69
1280.67
1257.13
1173.26
1136.78
1085.01
998.46
920.53
882.64
849.00
792.59
760.40
737.65
685.10
659.13
592.38
551.50
498.53
415.84

Figure S6: ^{1}H NMR spectrum of compound 3

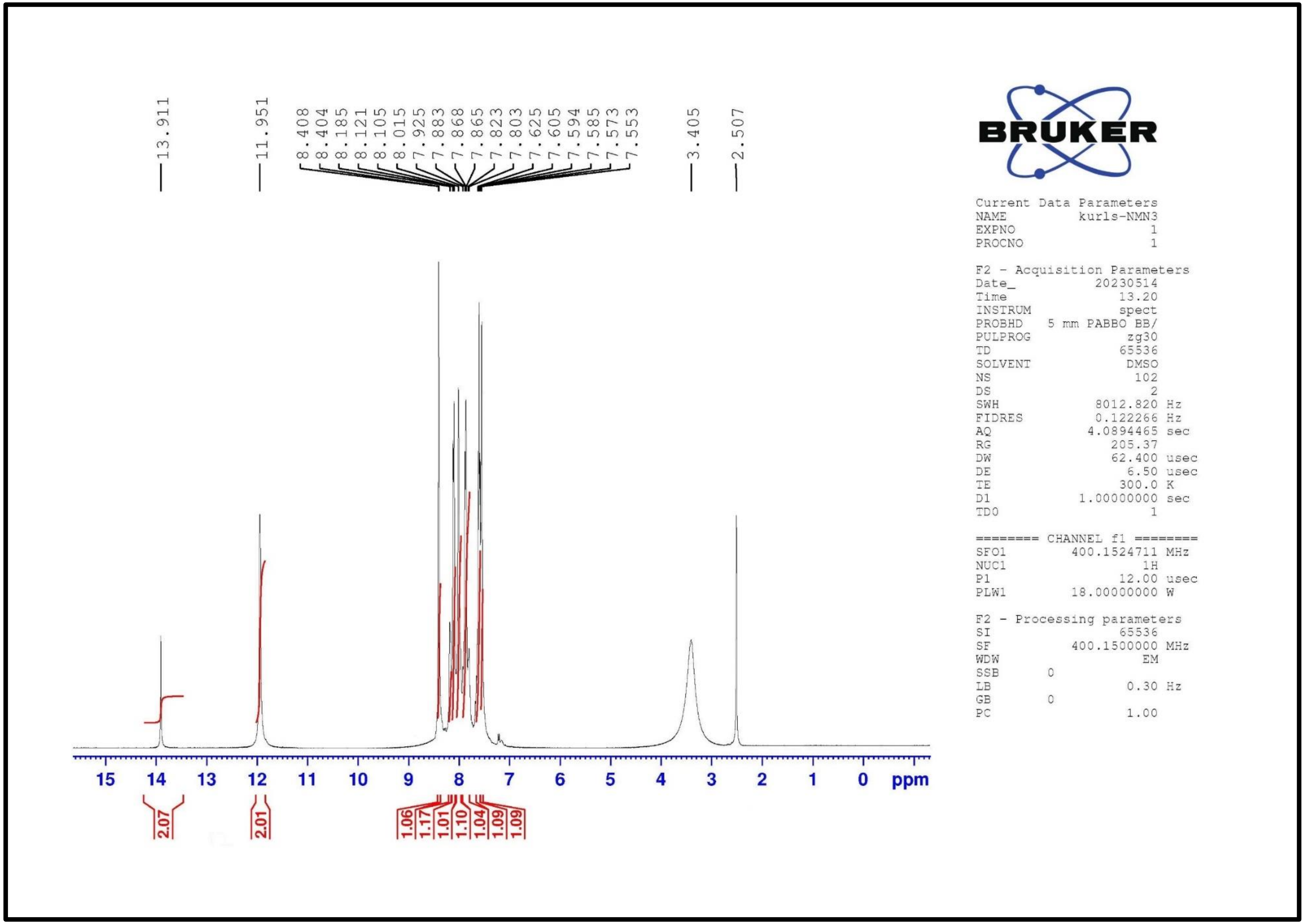

Figure S7: Expanded ^1H NMR spectrum of compound 3

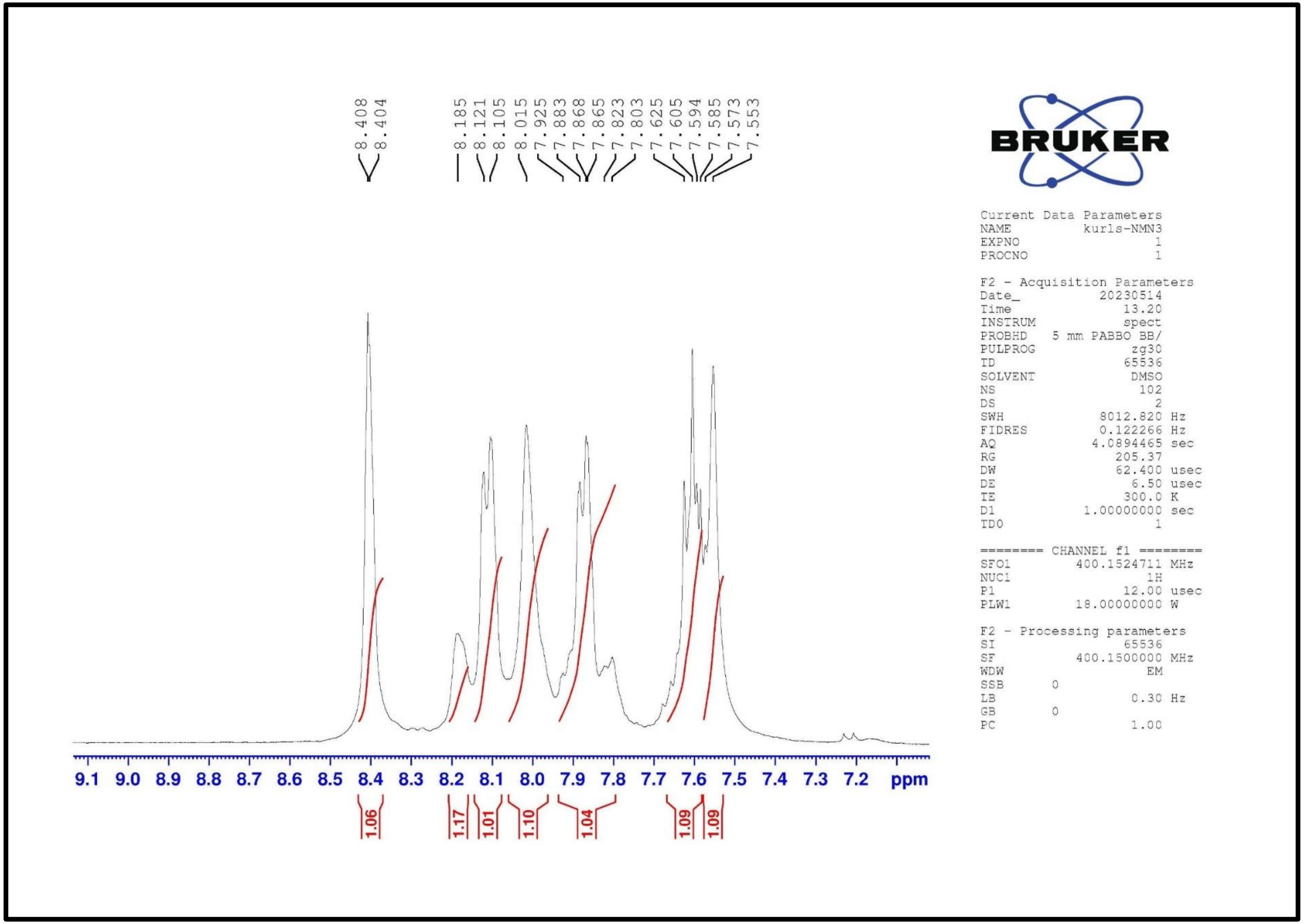

Figure S8: ^{13}C NMR spectrum of compound 3

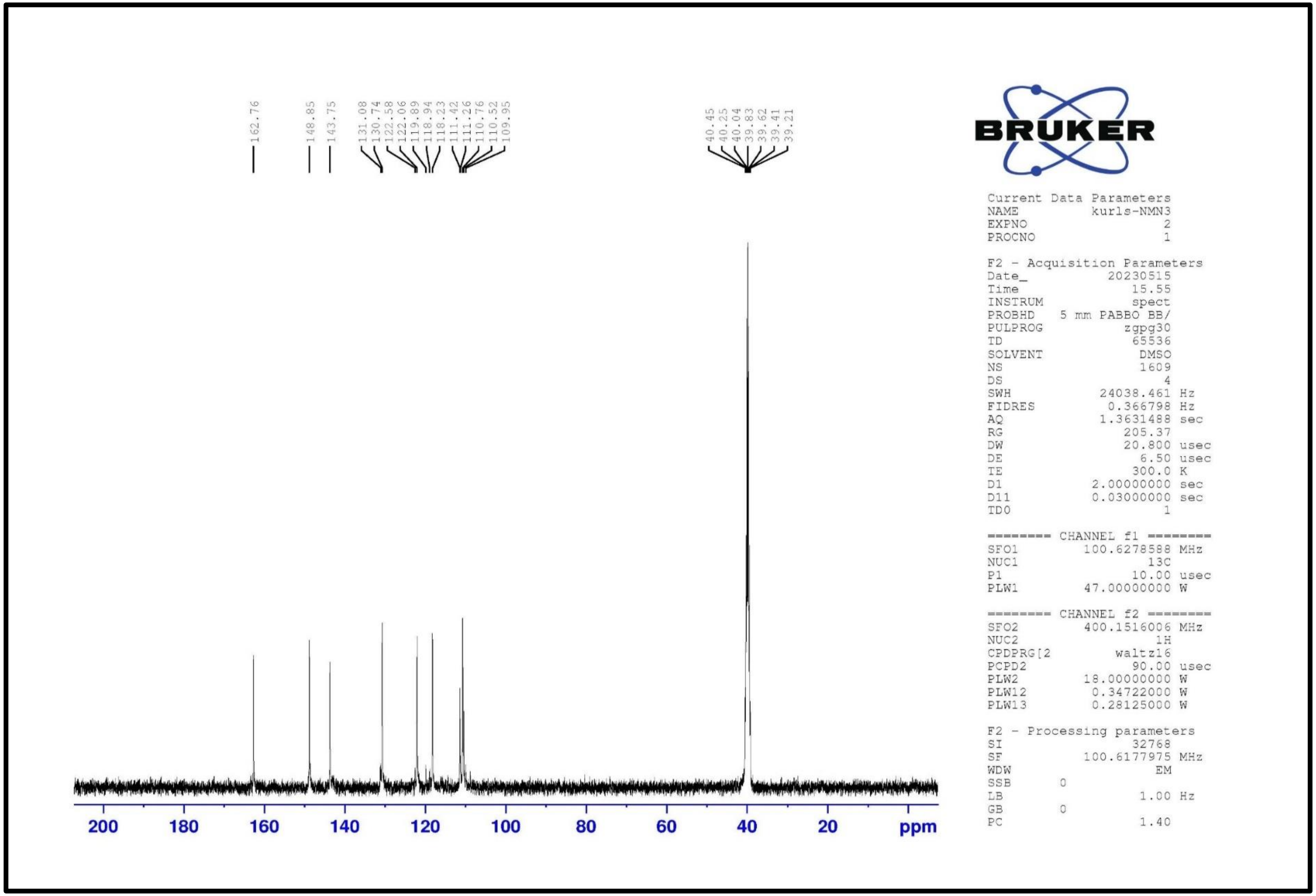

Figure S9: Mass spectrum of compound 3

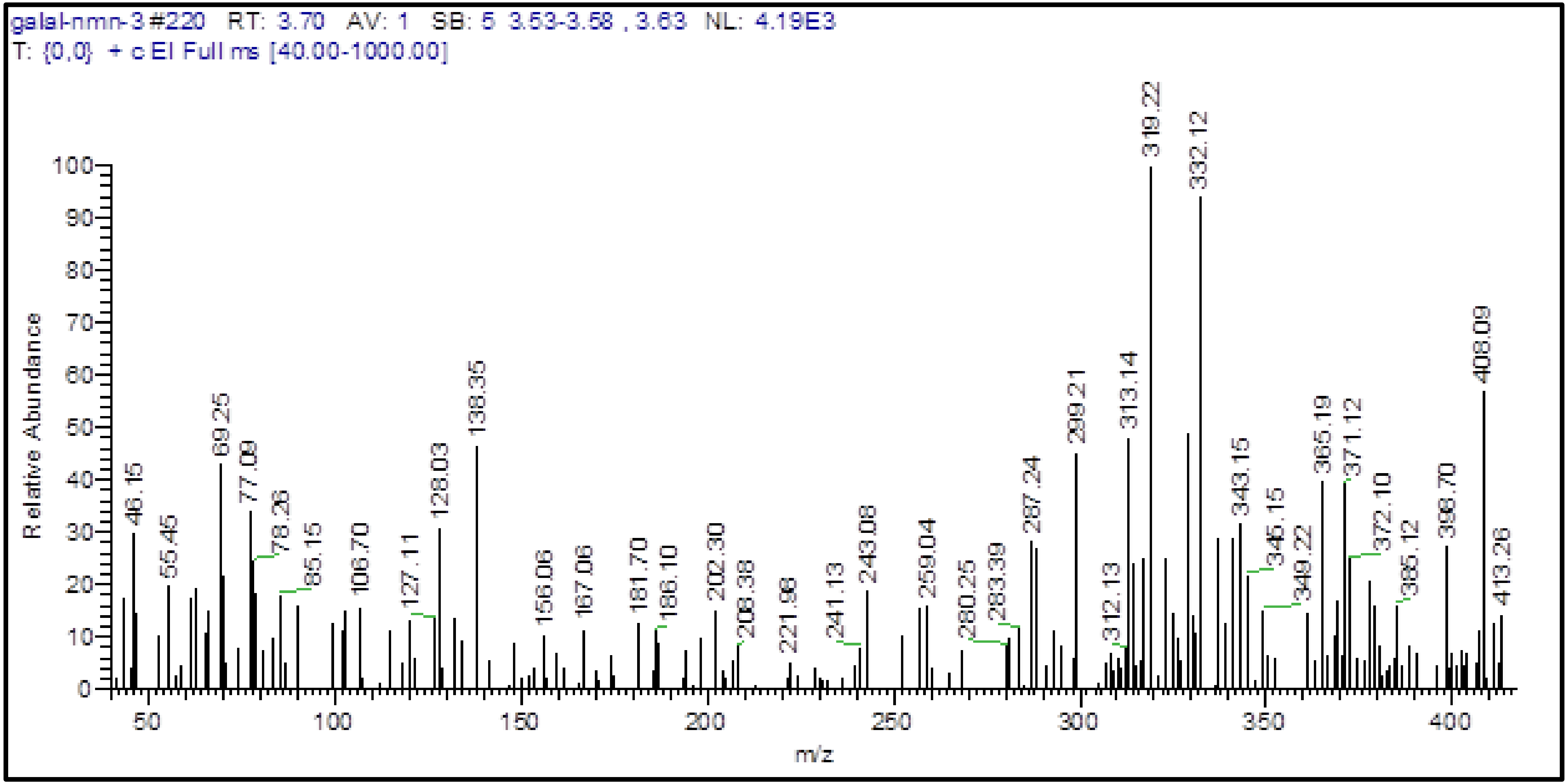

Figure S10: IR spectrum of compound 4

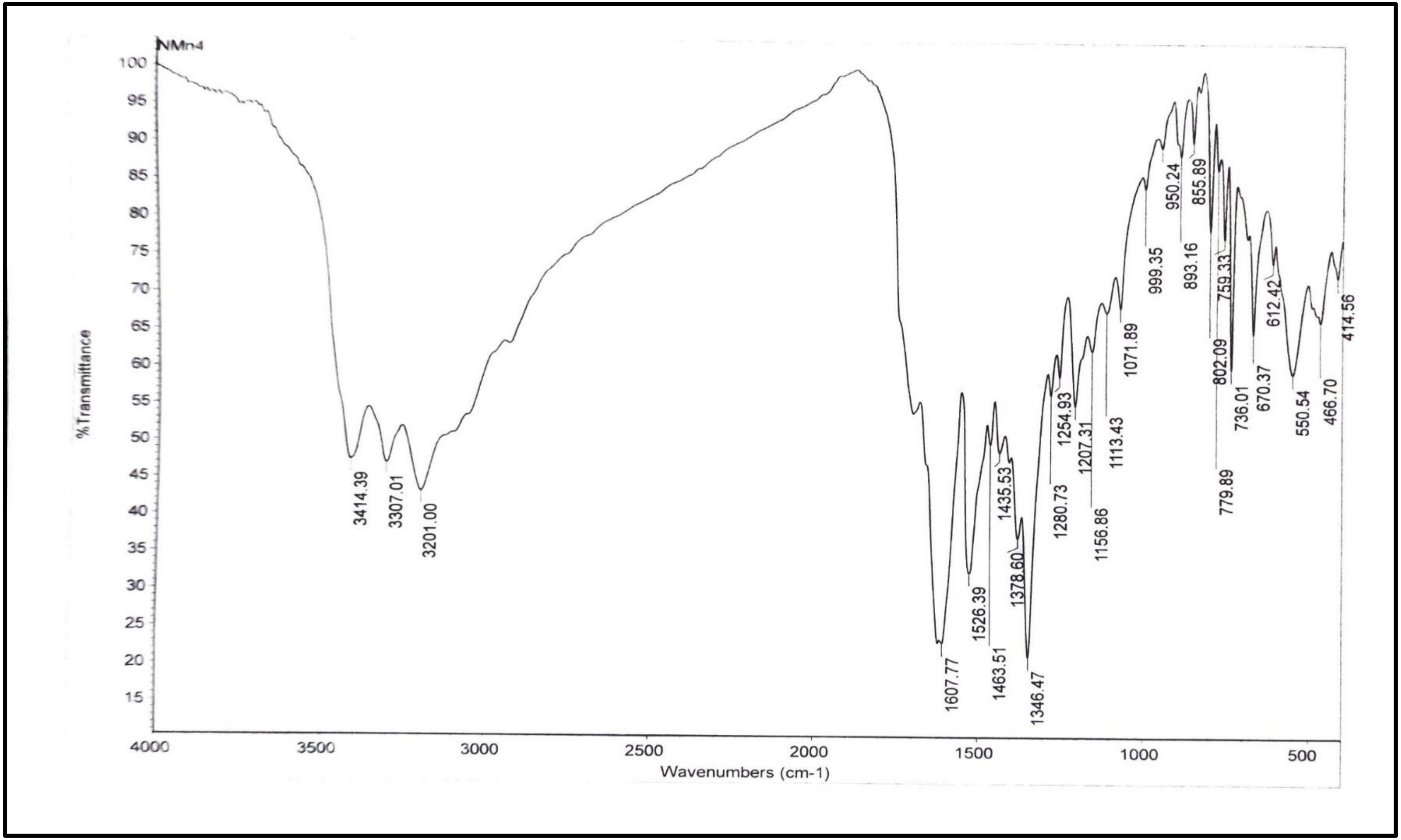

Figure S11: ^{1}H NMR spectrum of compound 4

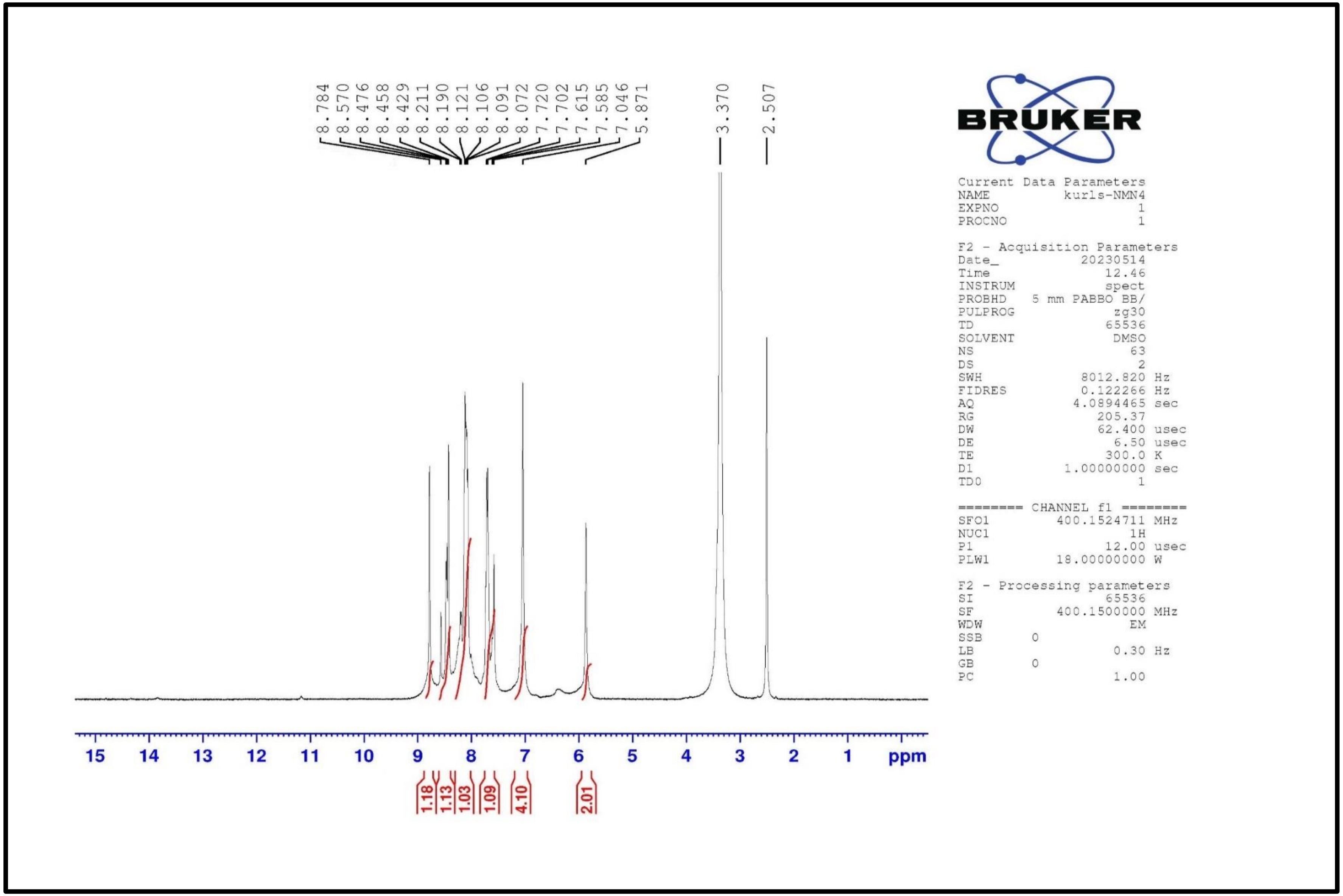

Figure S12: Expanded ^{1}H NMR spectrum of compound 4

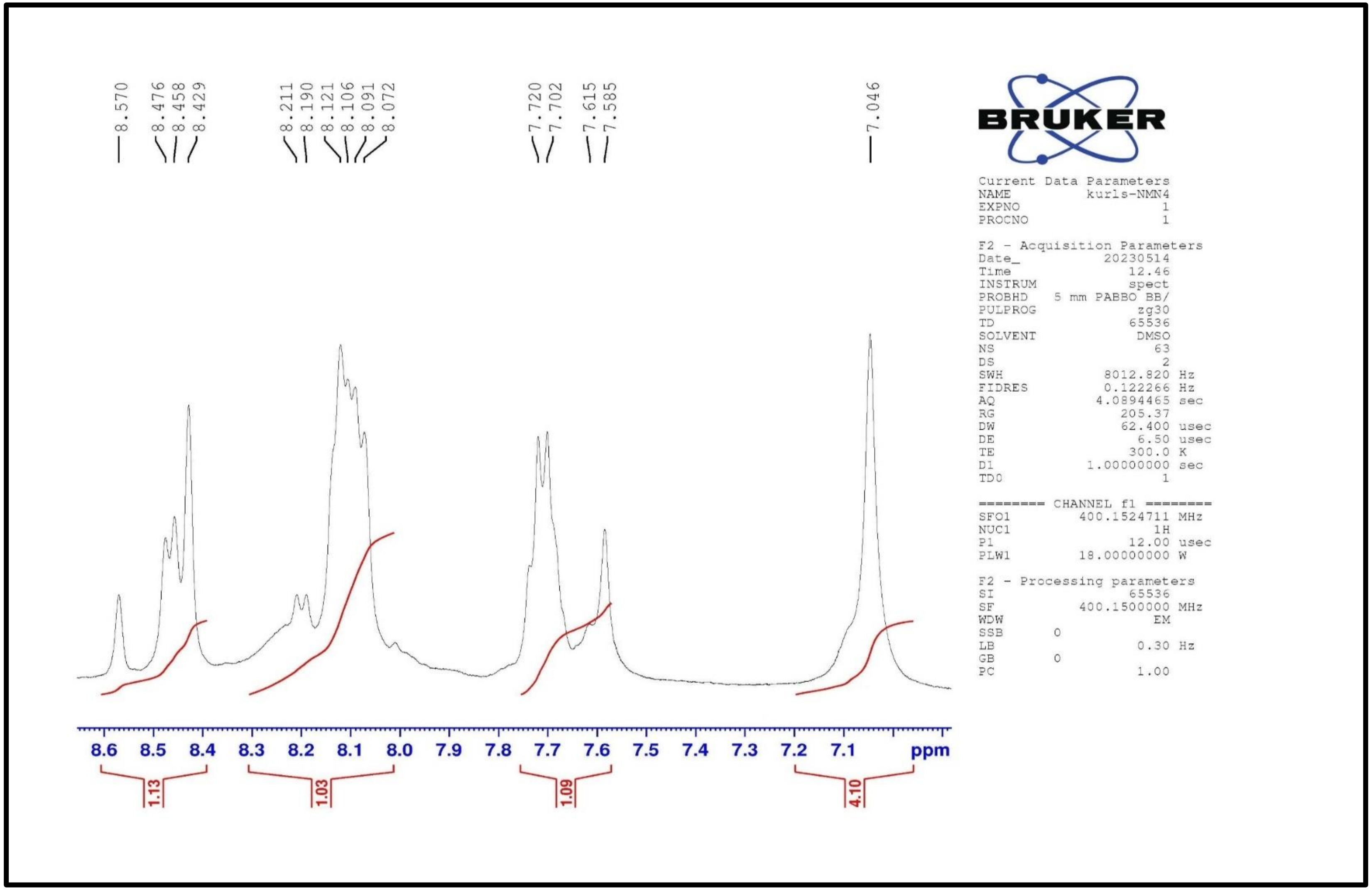

Figure S13: ^{13}C NMR spectrum of compound 4

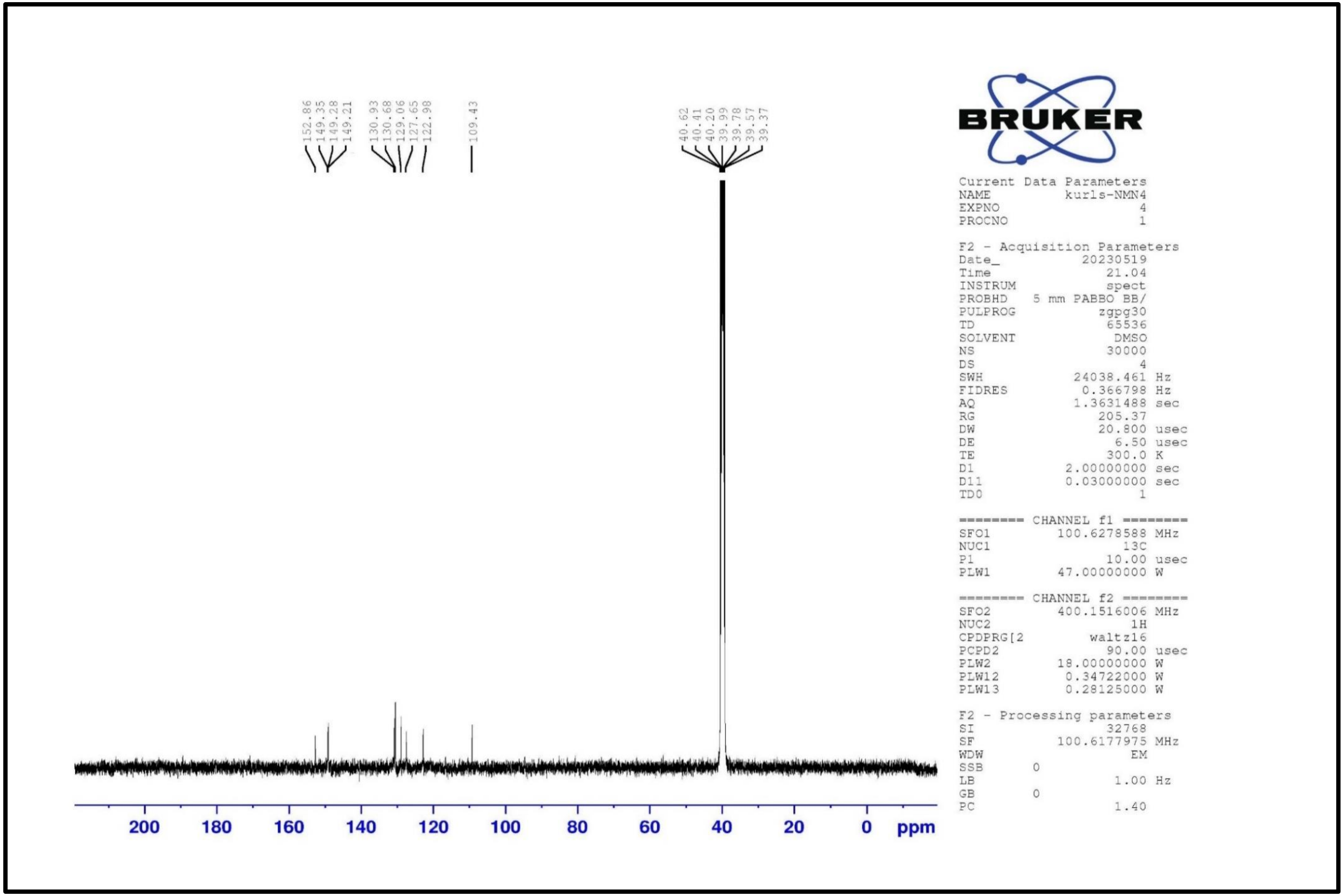

Figure S14: Mass spectrum of compound 4

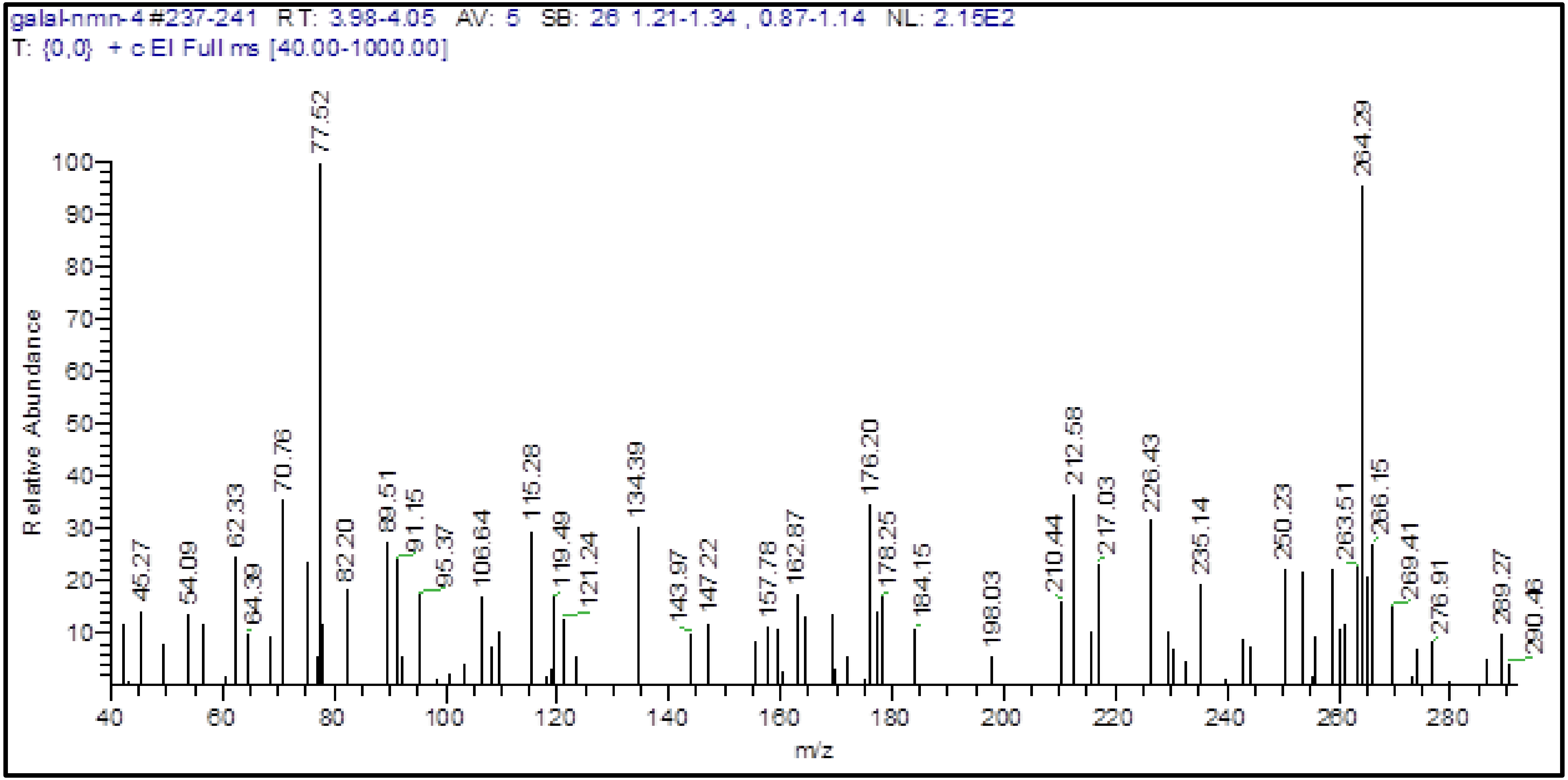

Figure S15: IR spectrum of compound 5

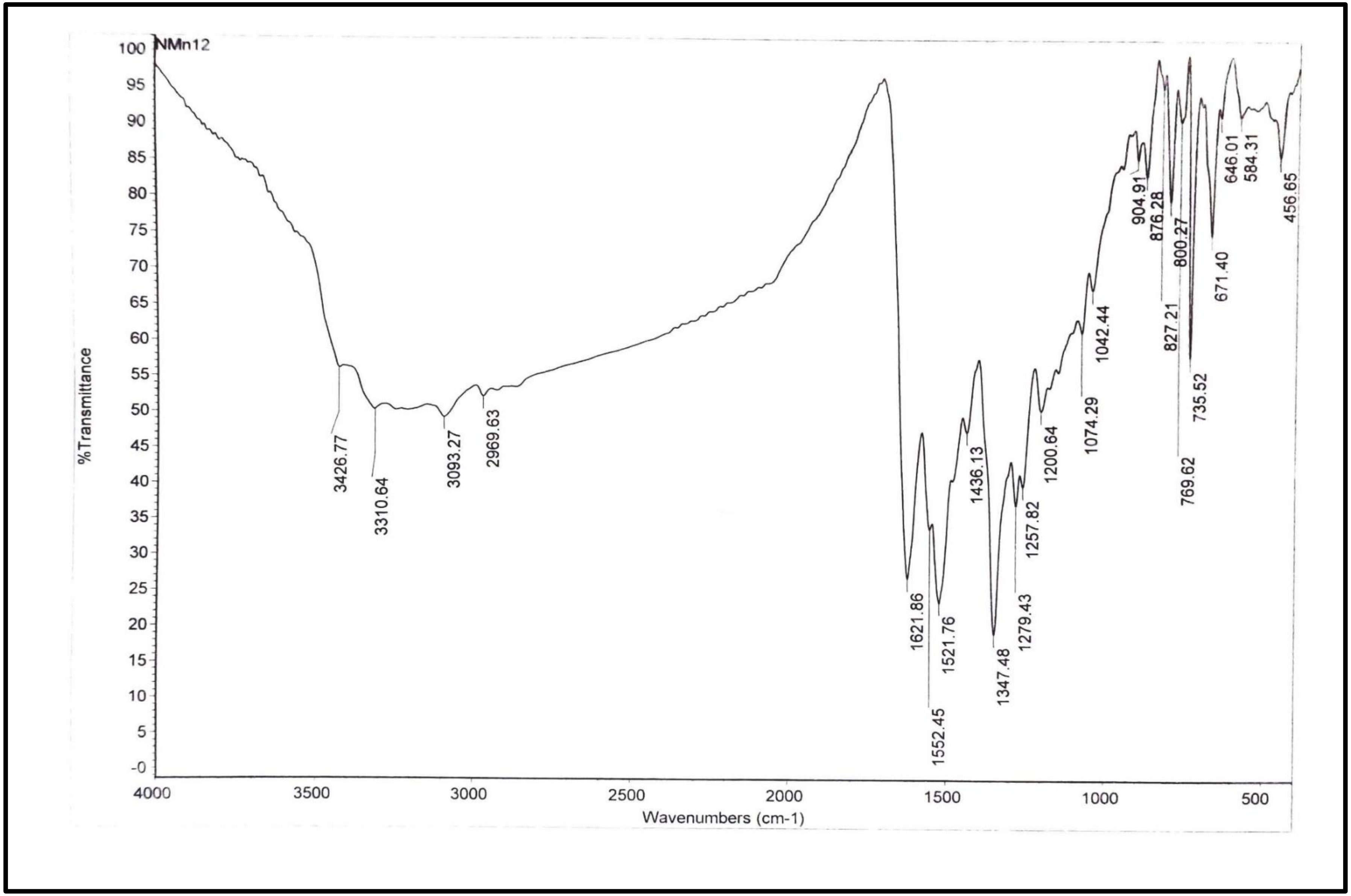

Figure S16: ^{1}H NMR spectrum of compound 5

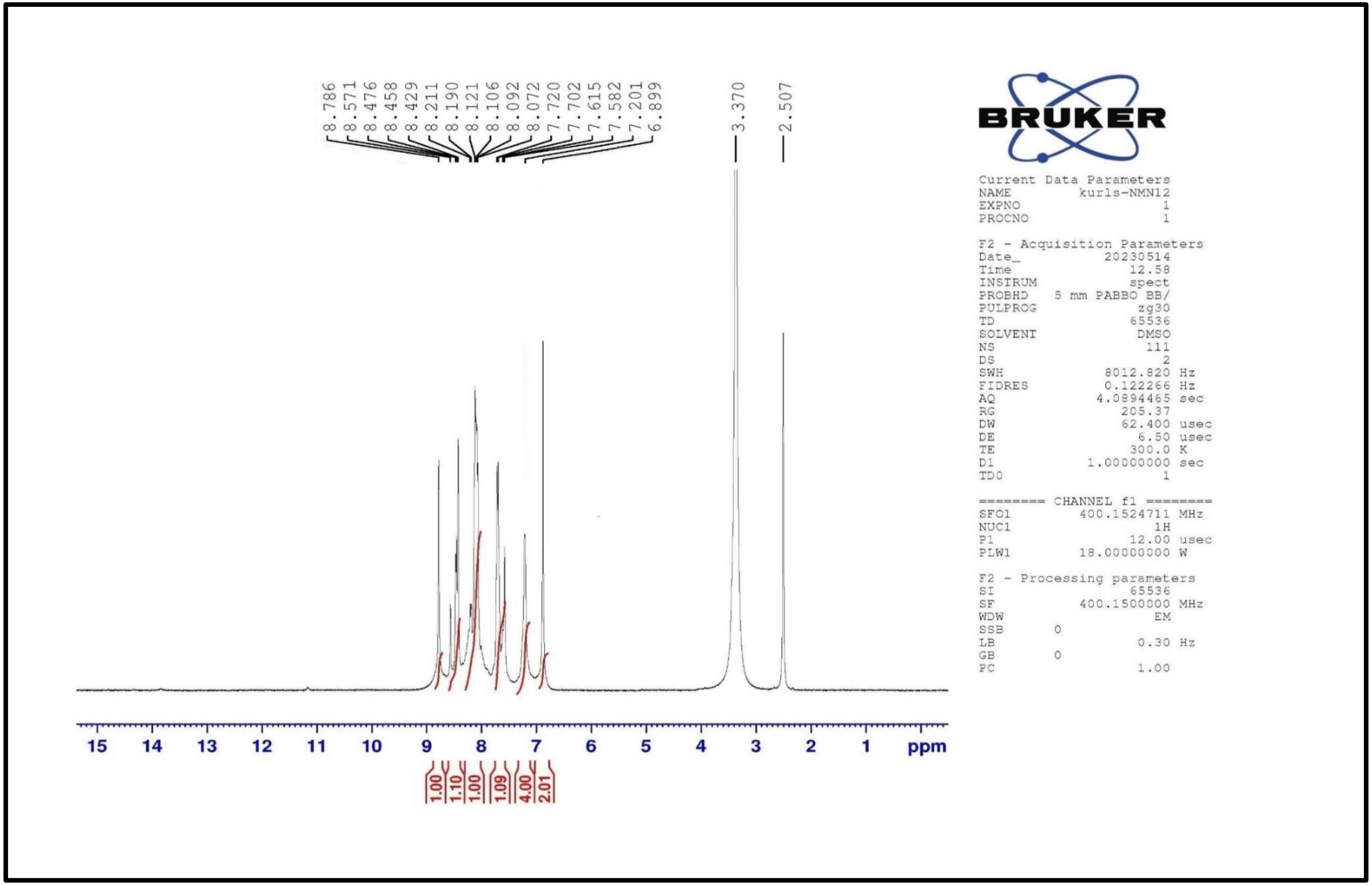

Figure S17: ^{13}C NMR spectrum of compound 5

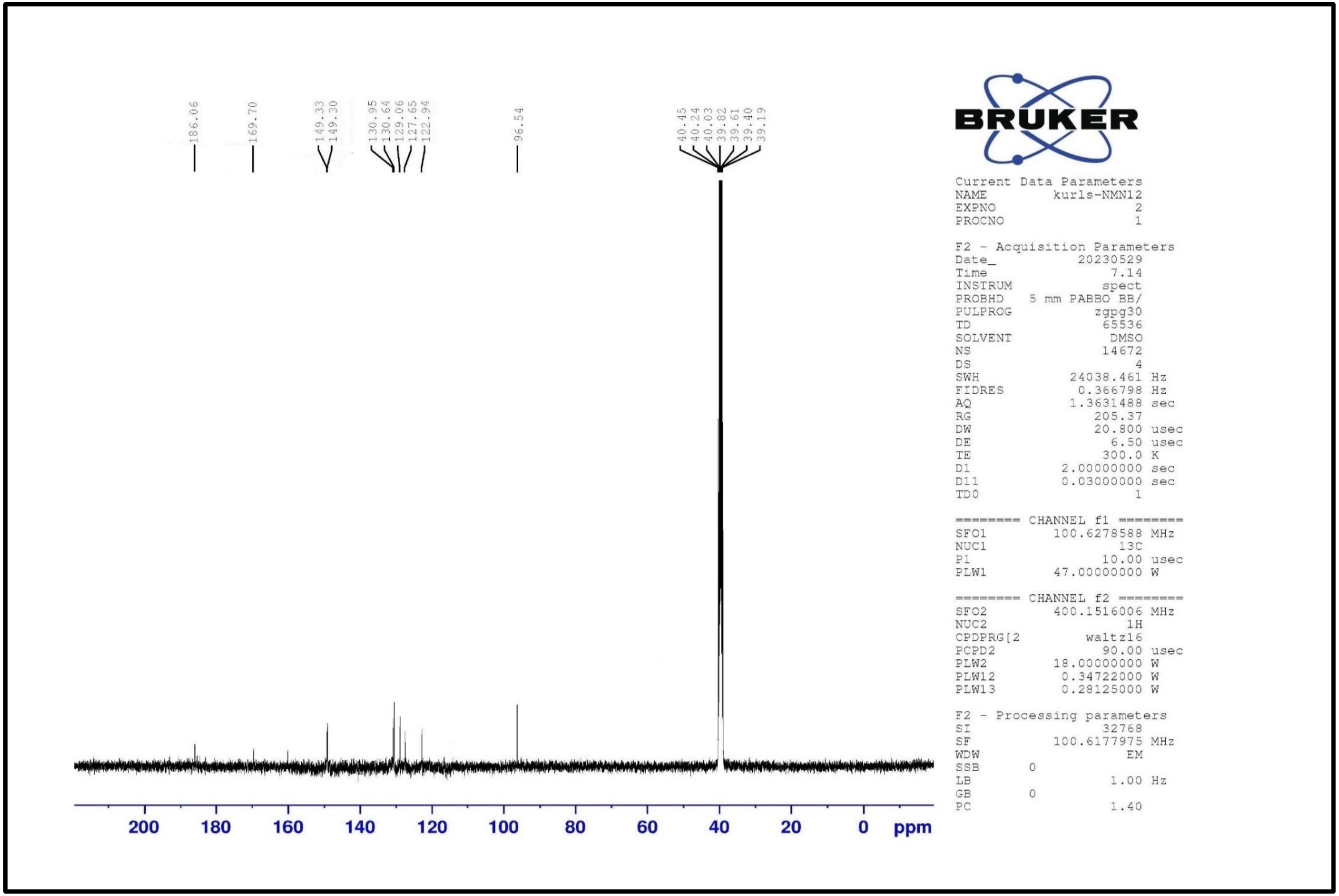

Figure S18: Mass spectrum of compound 5

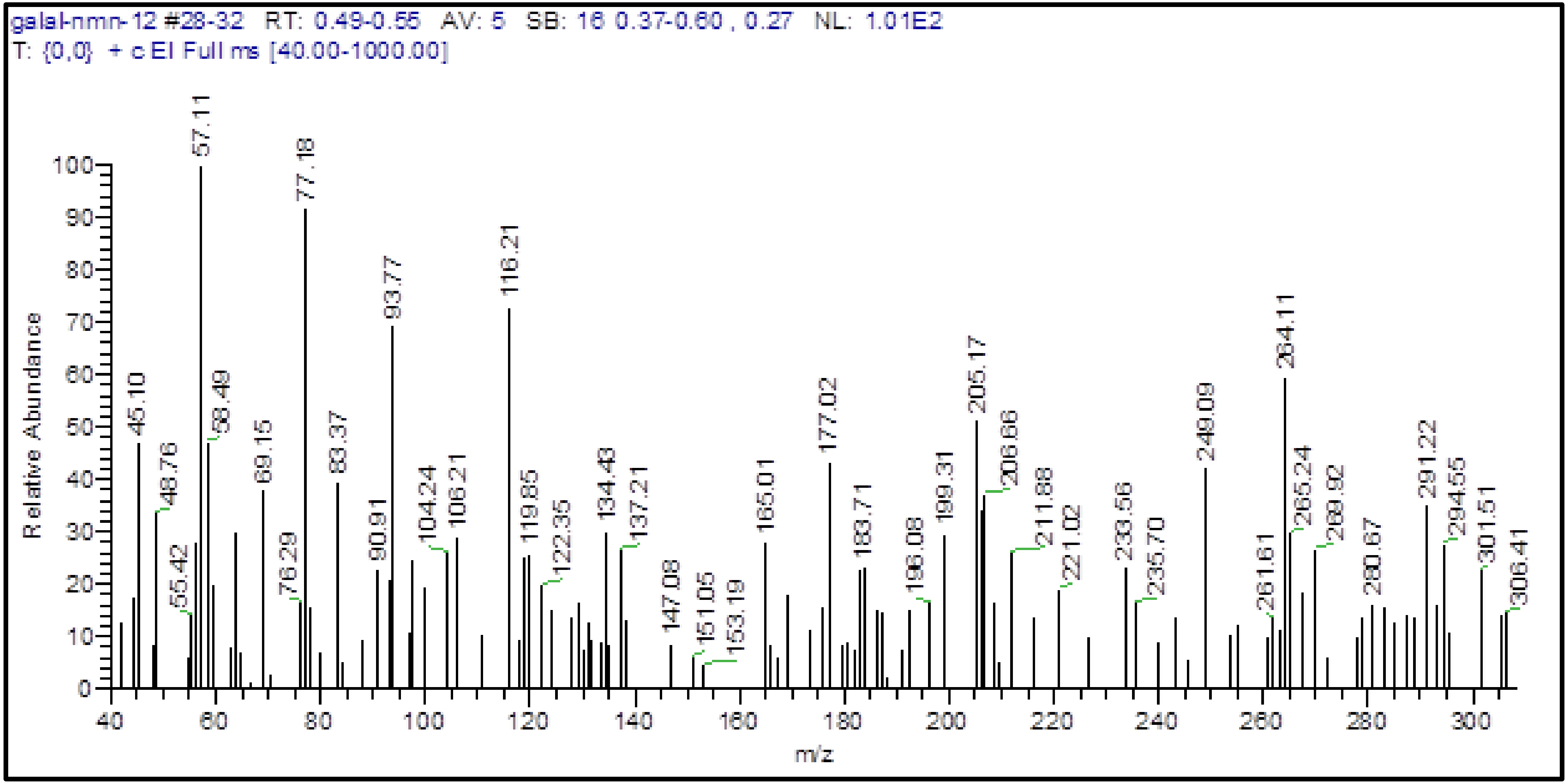

Figure S19: IR spectrum of compound 6

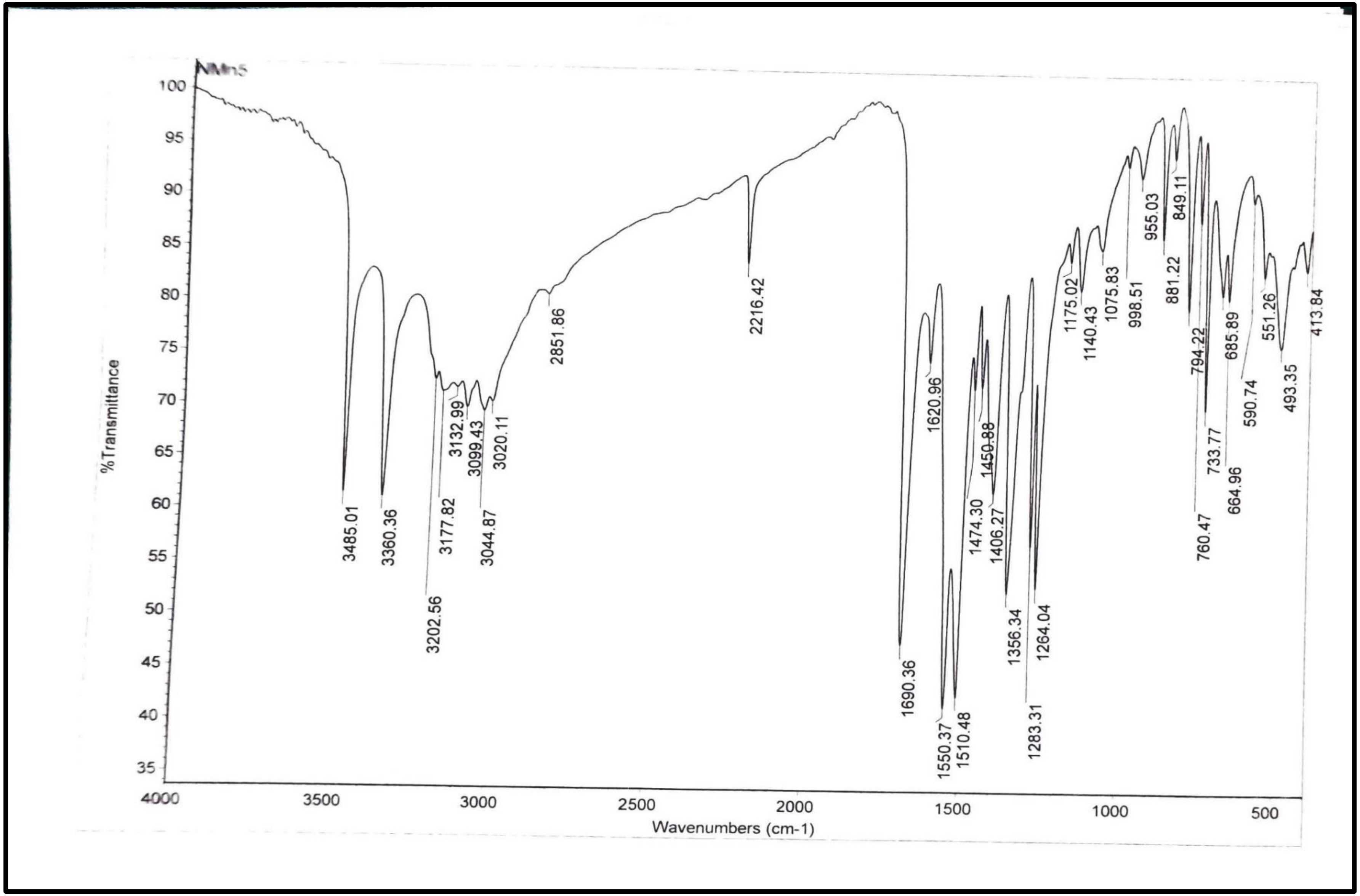

Figure S20: ^{1}H NMR spectrum of compound 6

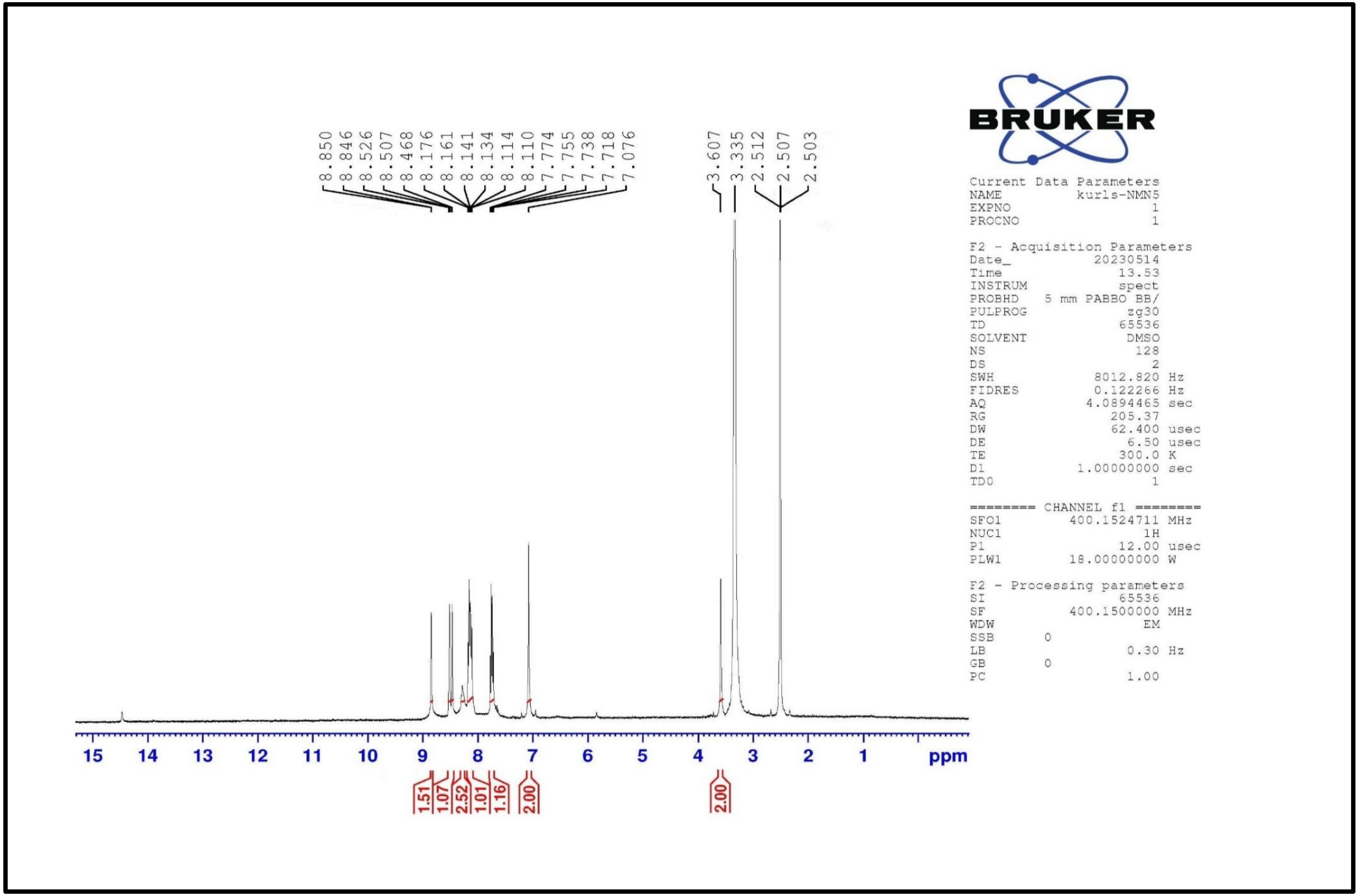

Figure S21: ^{13}C NMR spectrum of compound 6

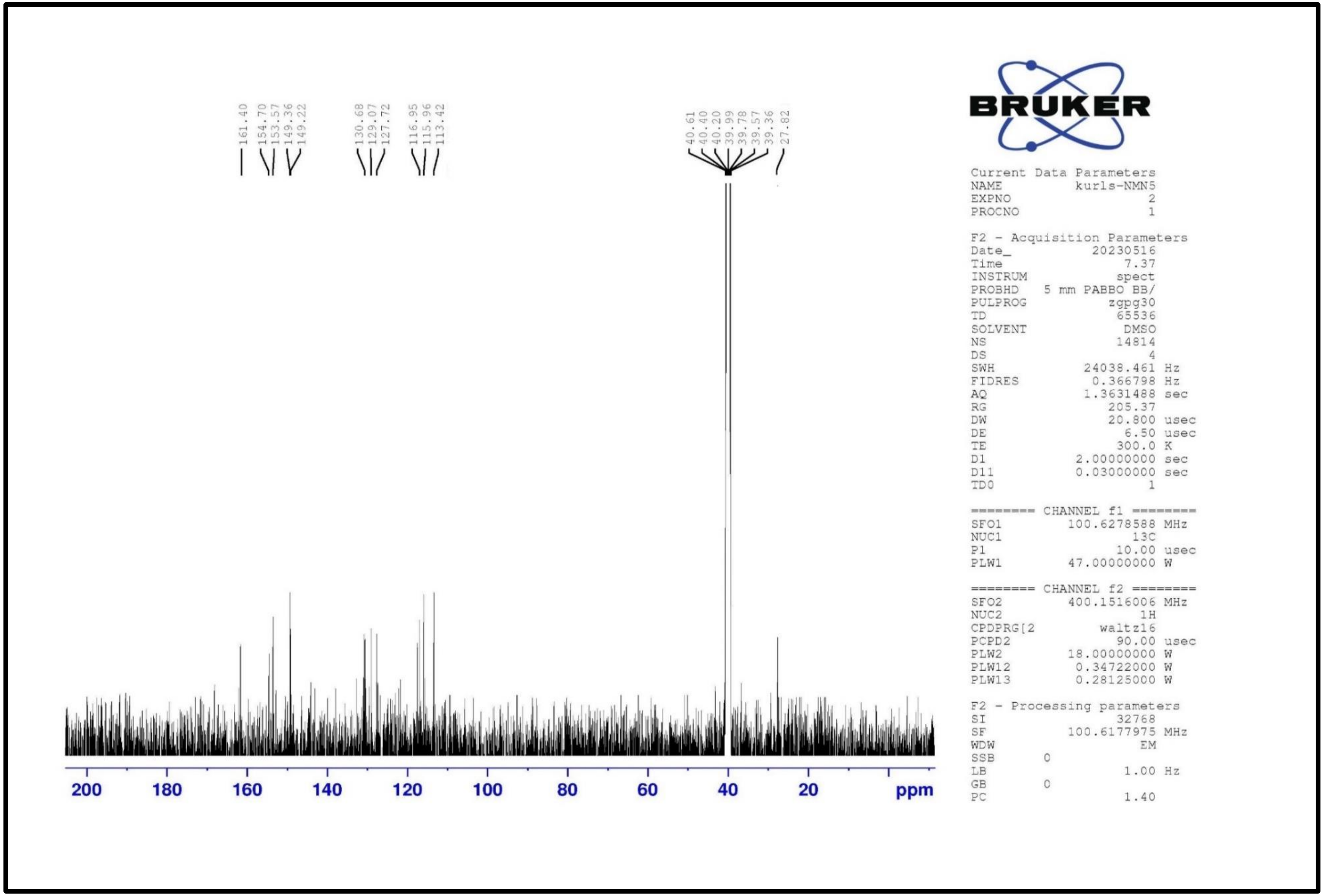

Figure S22: Mass spectrum of compound 6

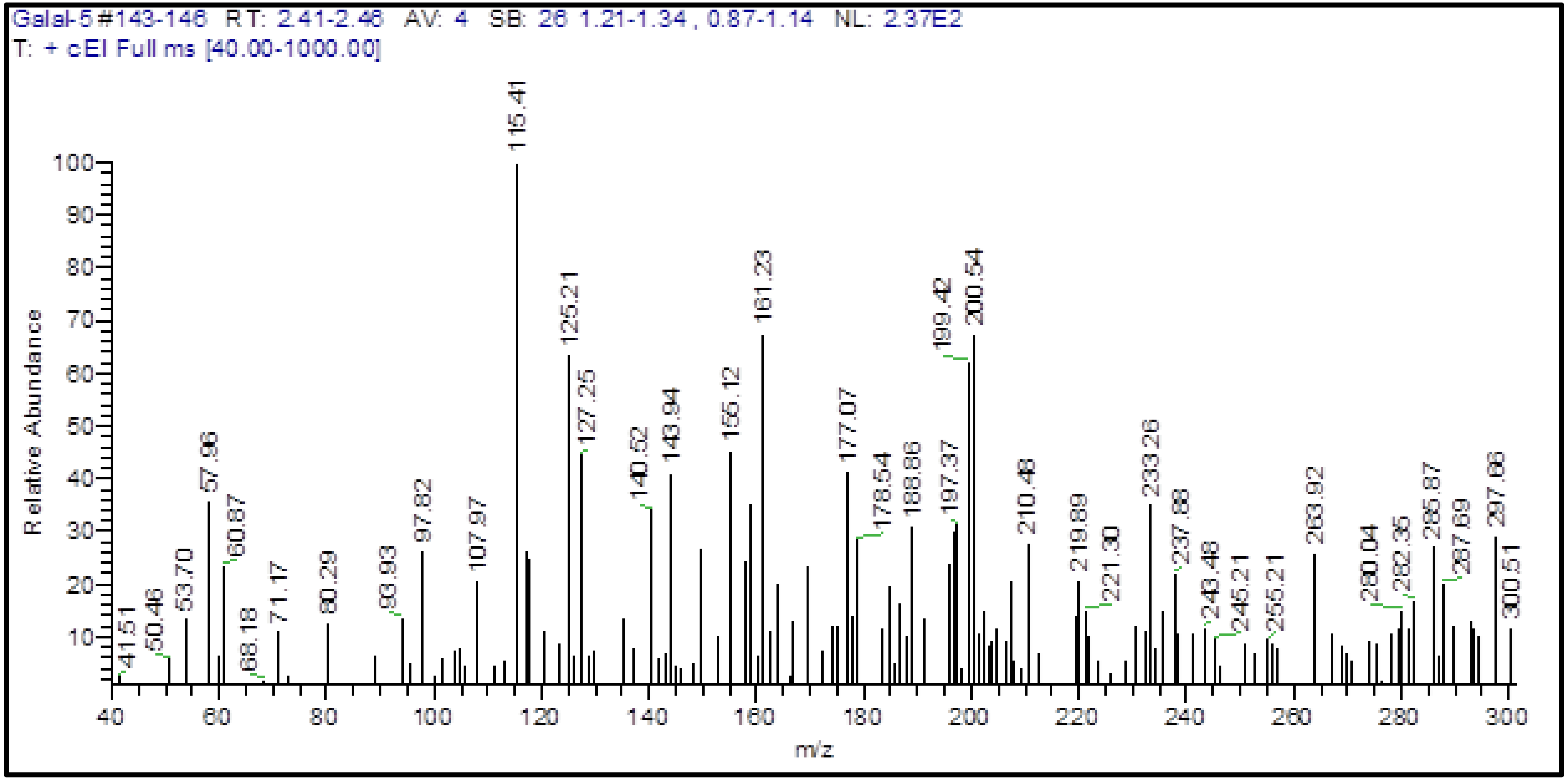

Figure S23: IR spectrum of compound 7

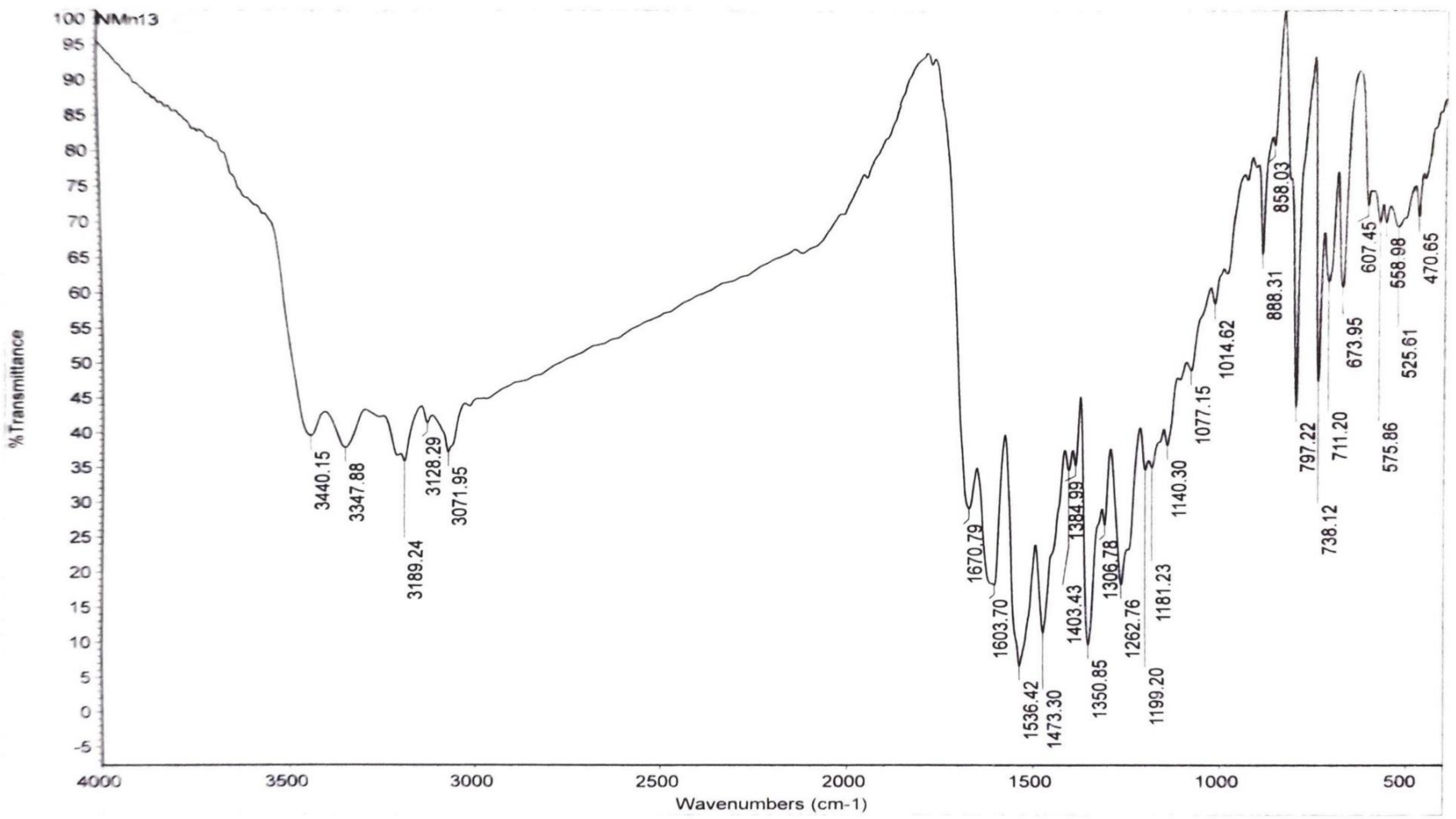

Figure S24: ^{1}H NMR spectrum of compound 7

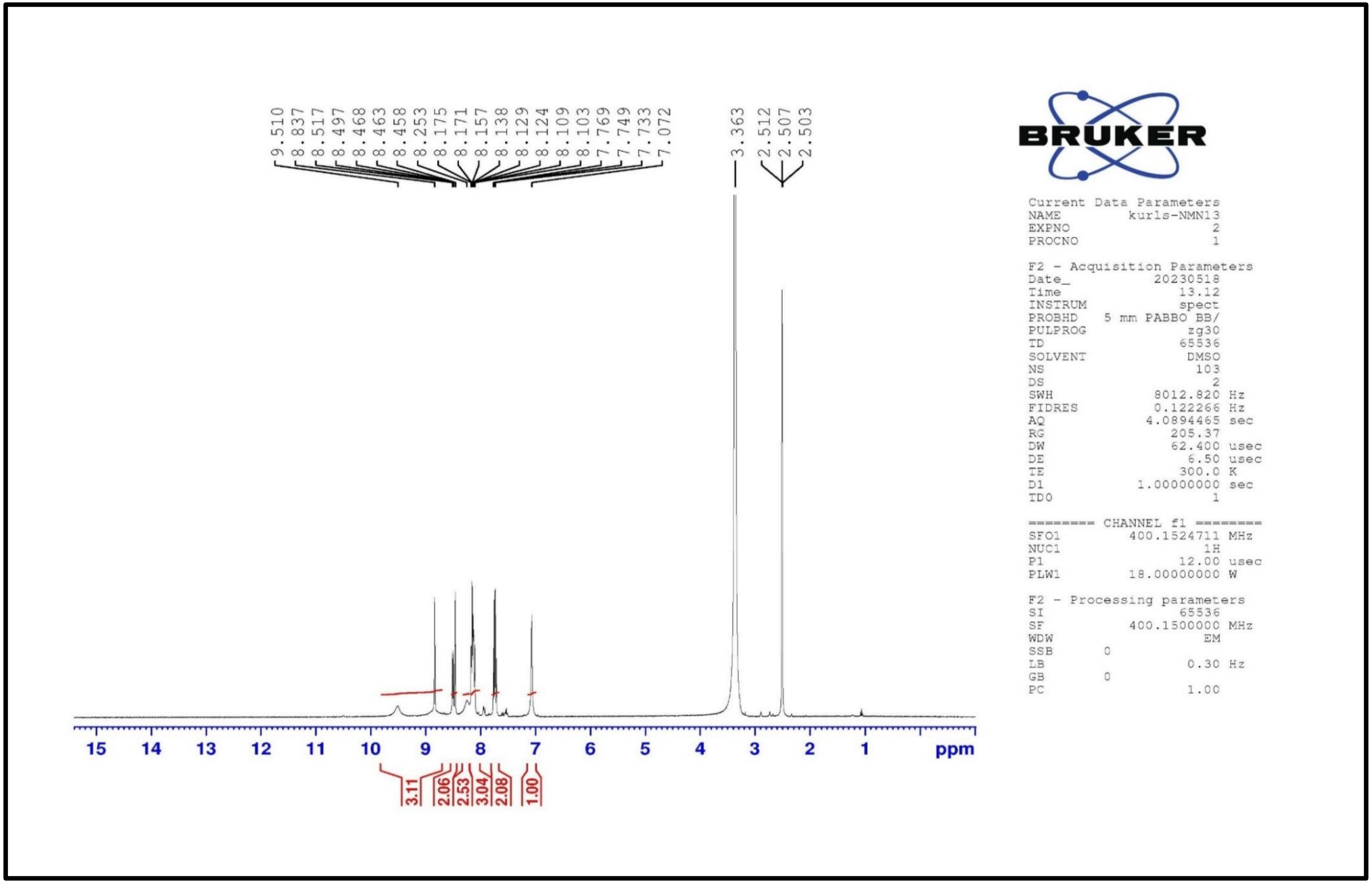

Figure S25: D_2O-^1H NMR spectrum of compound 7

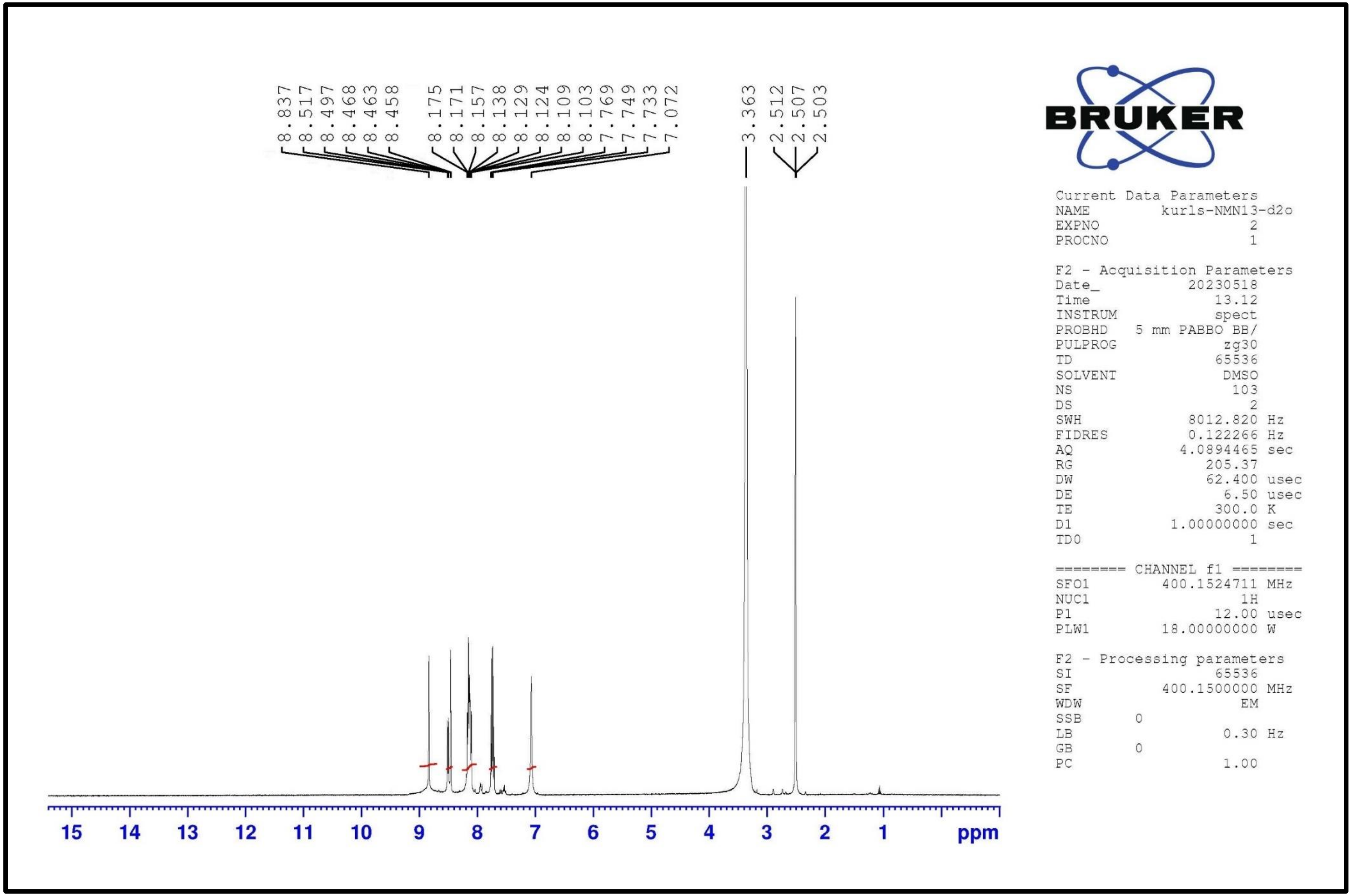

Figure S26: ^{13}C NMR spectrum of compound 7

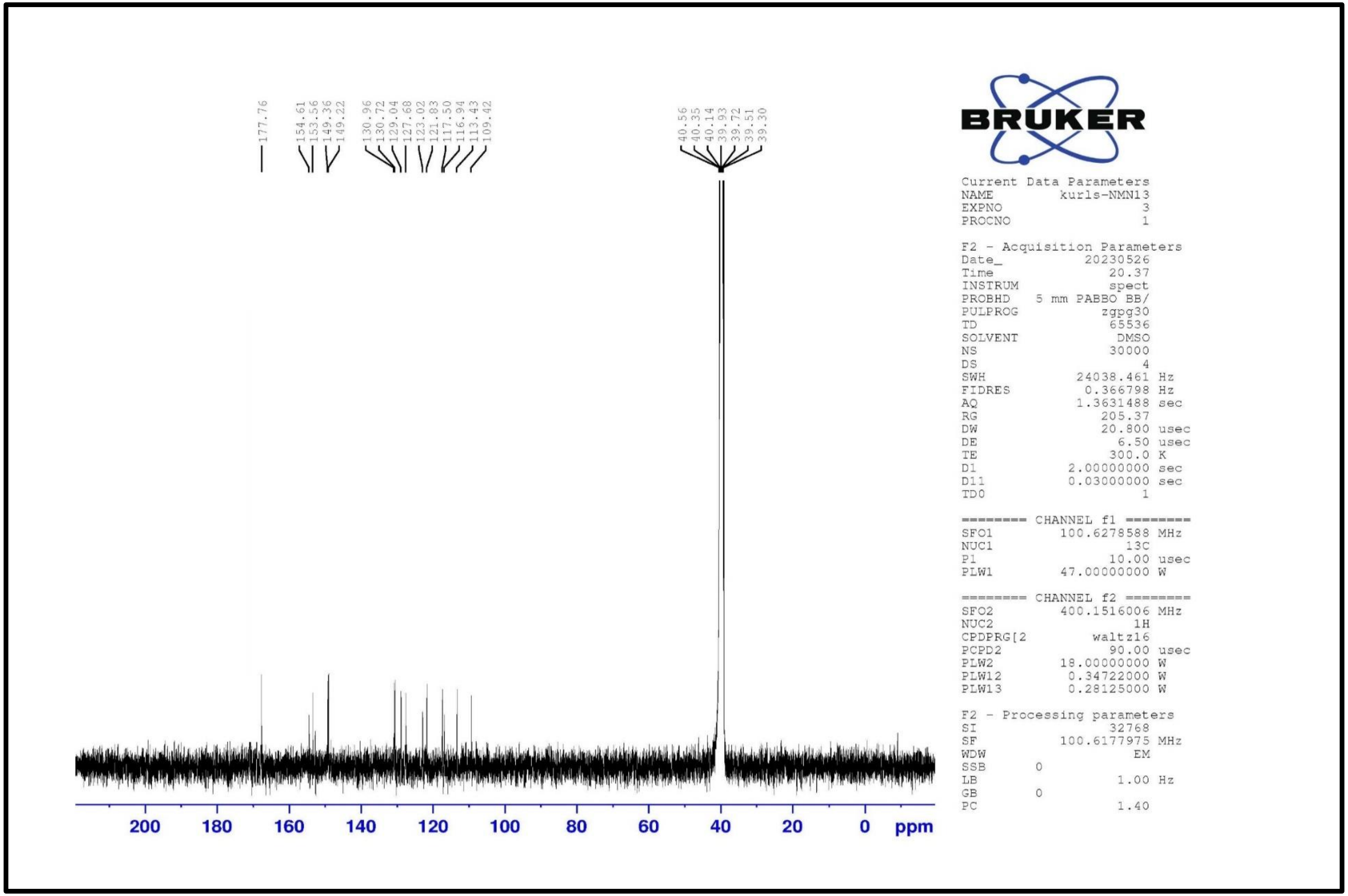

Figure S27: Mass spectrum of compound 7

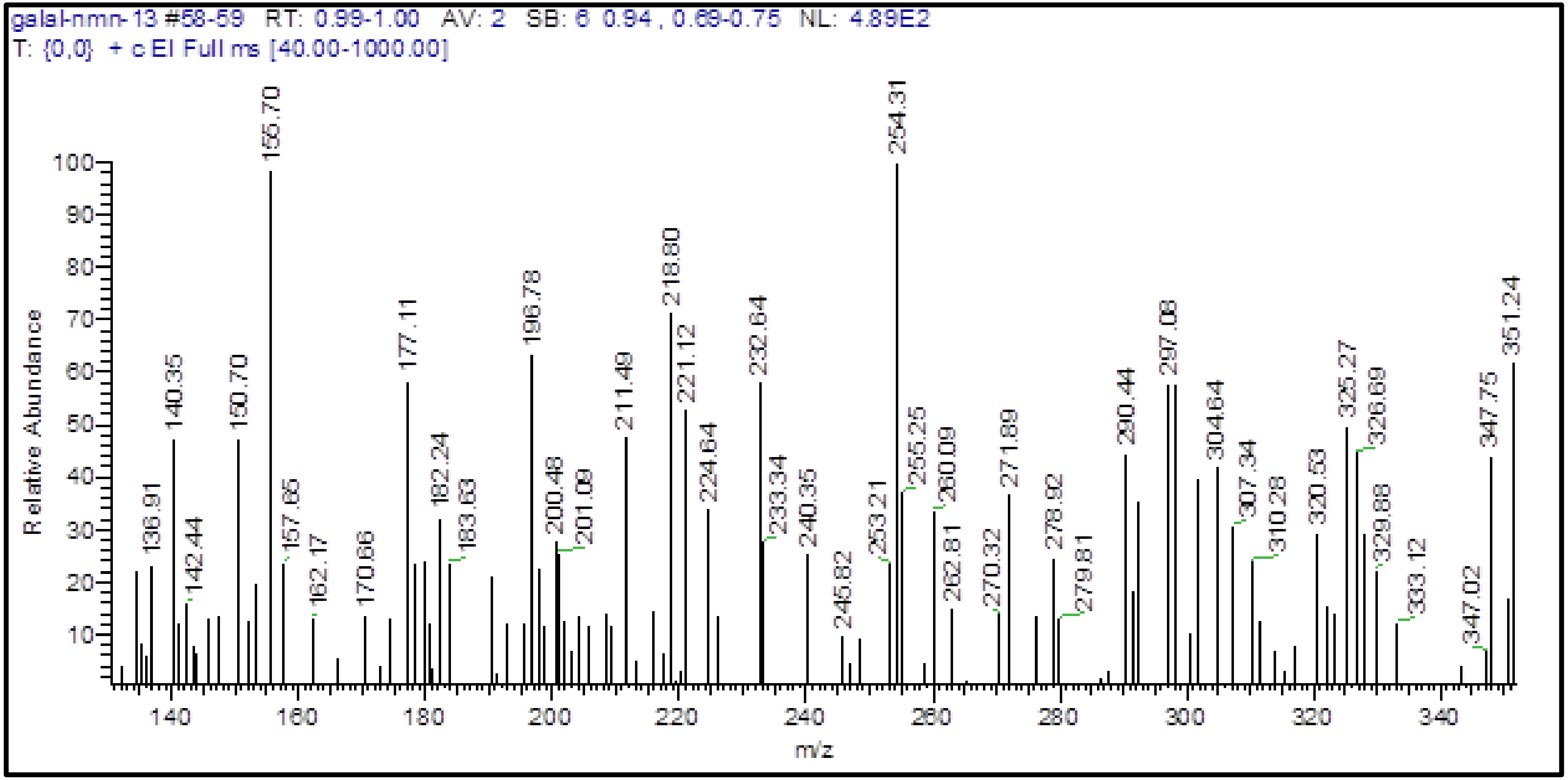

Figure S28: IR spectrum of compound 8

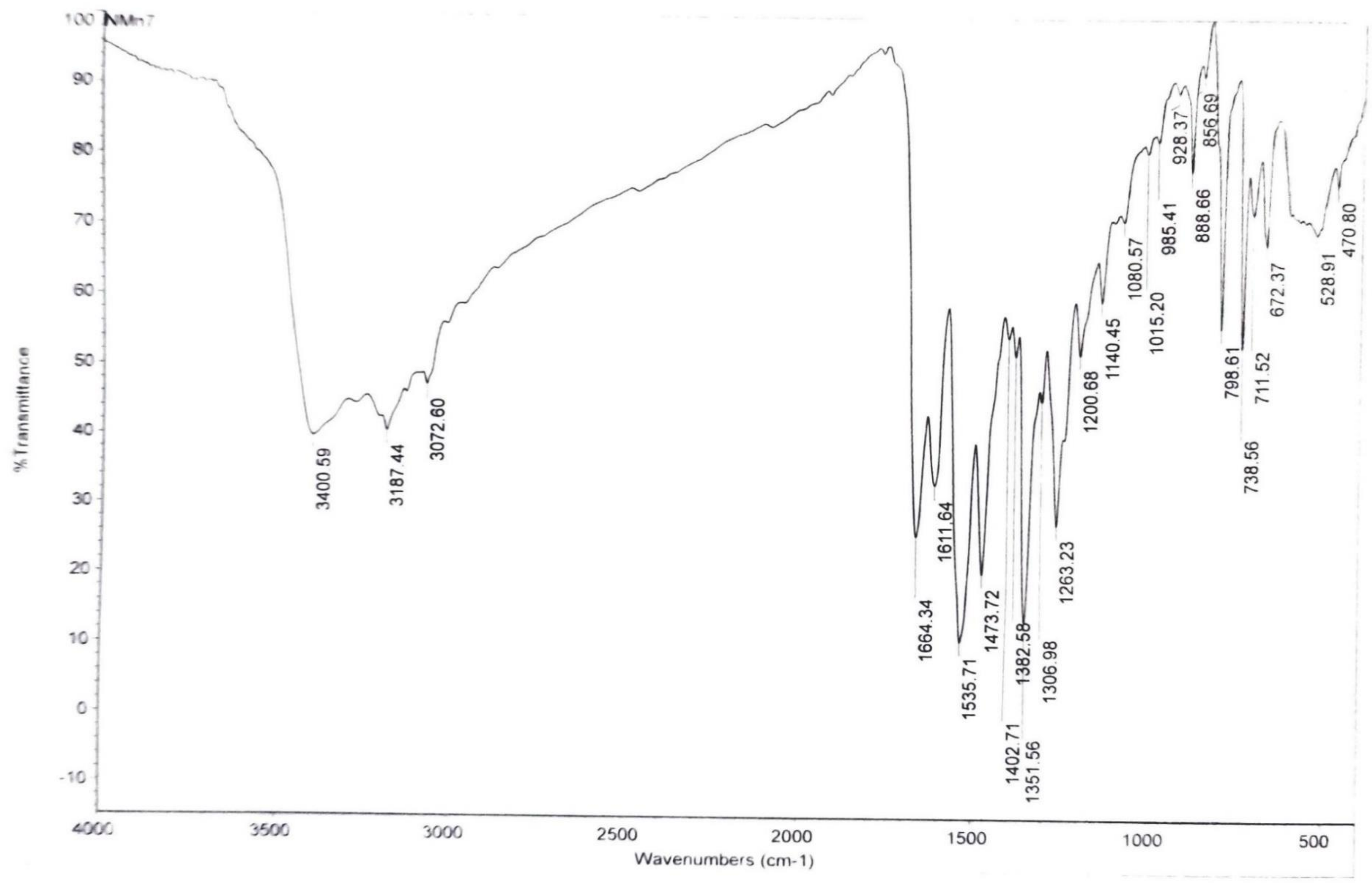

Figure S29: ^{1}H NMR spectrum of compound 8

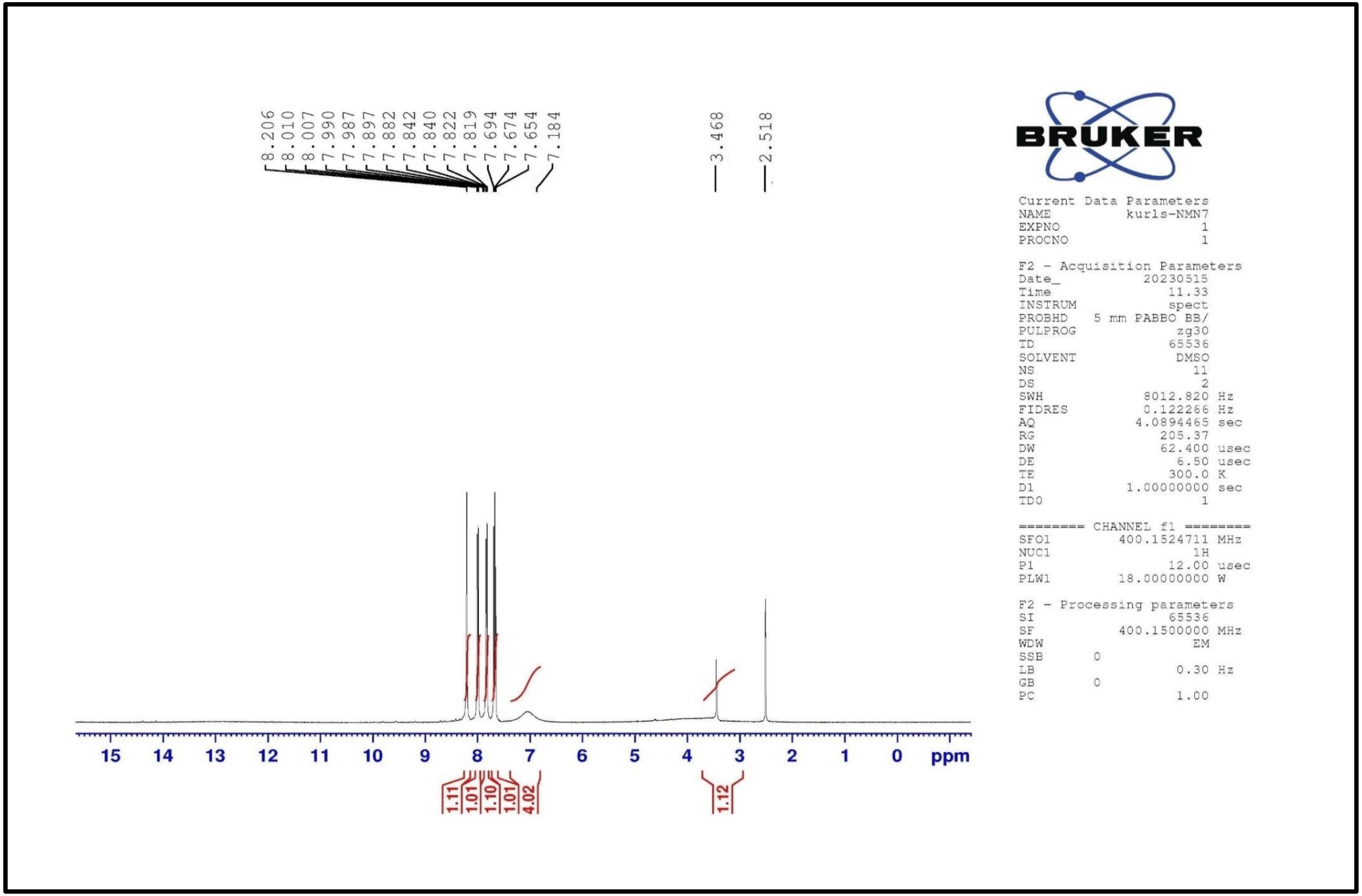

Figure S30: ^{13}C NMR spectrum of compound 8

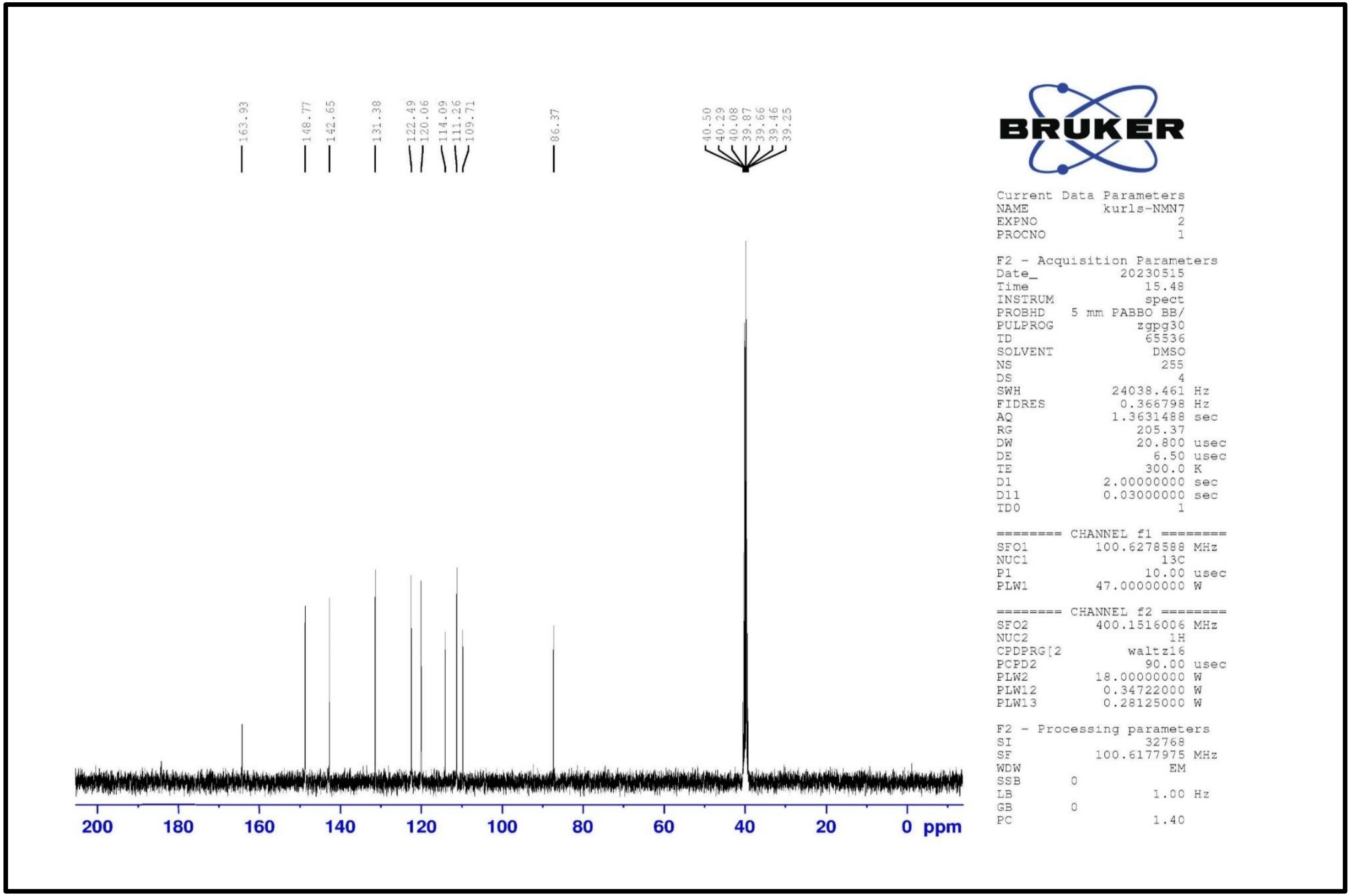

Figure S31: Mass spectrum of compound 8

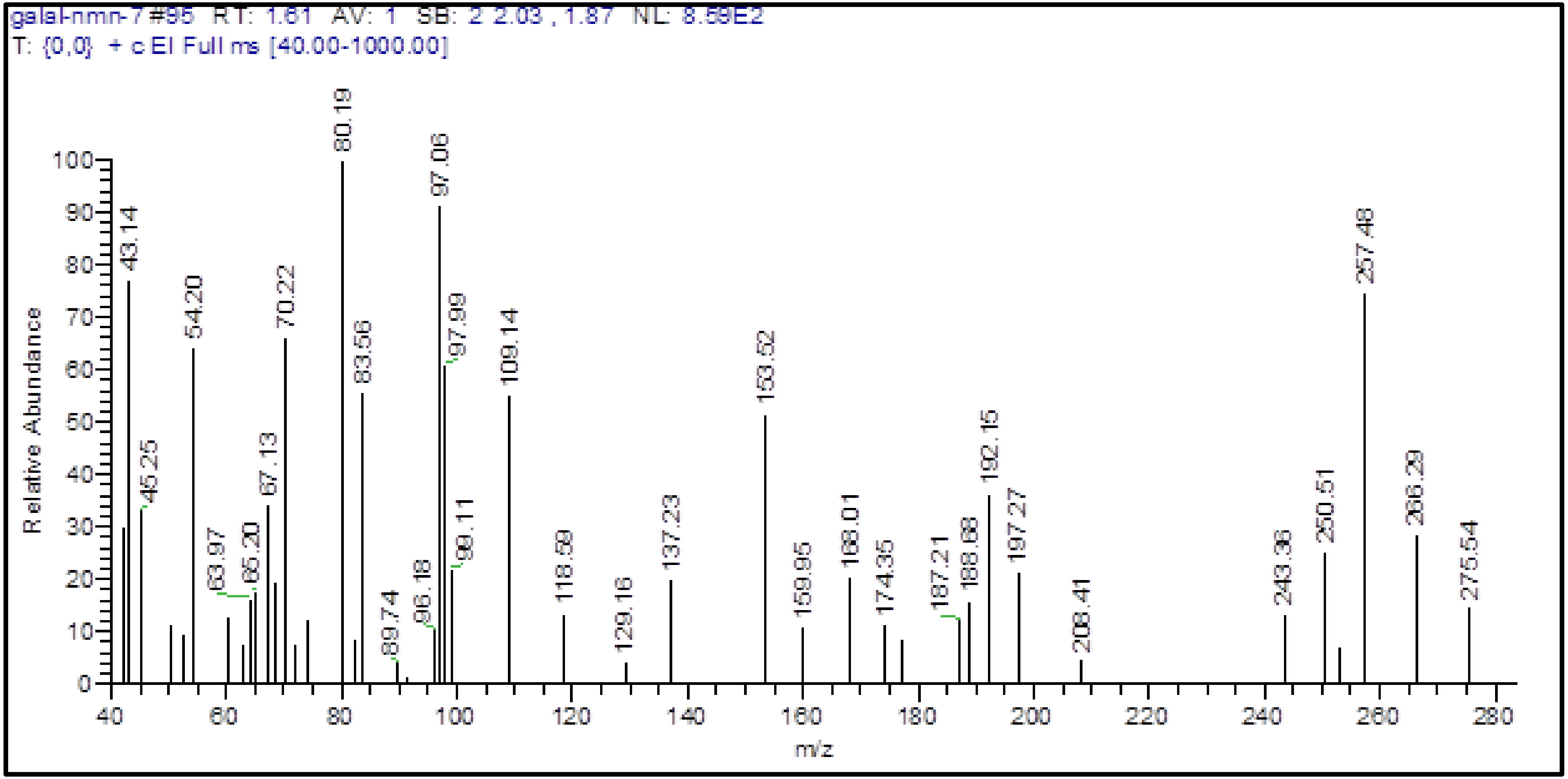

Figure S32: IR spectrum of compound 9

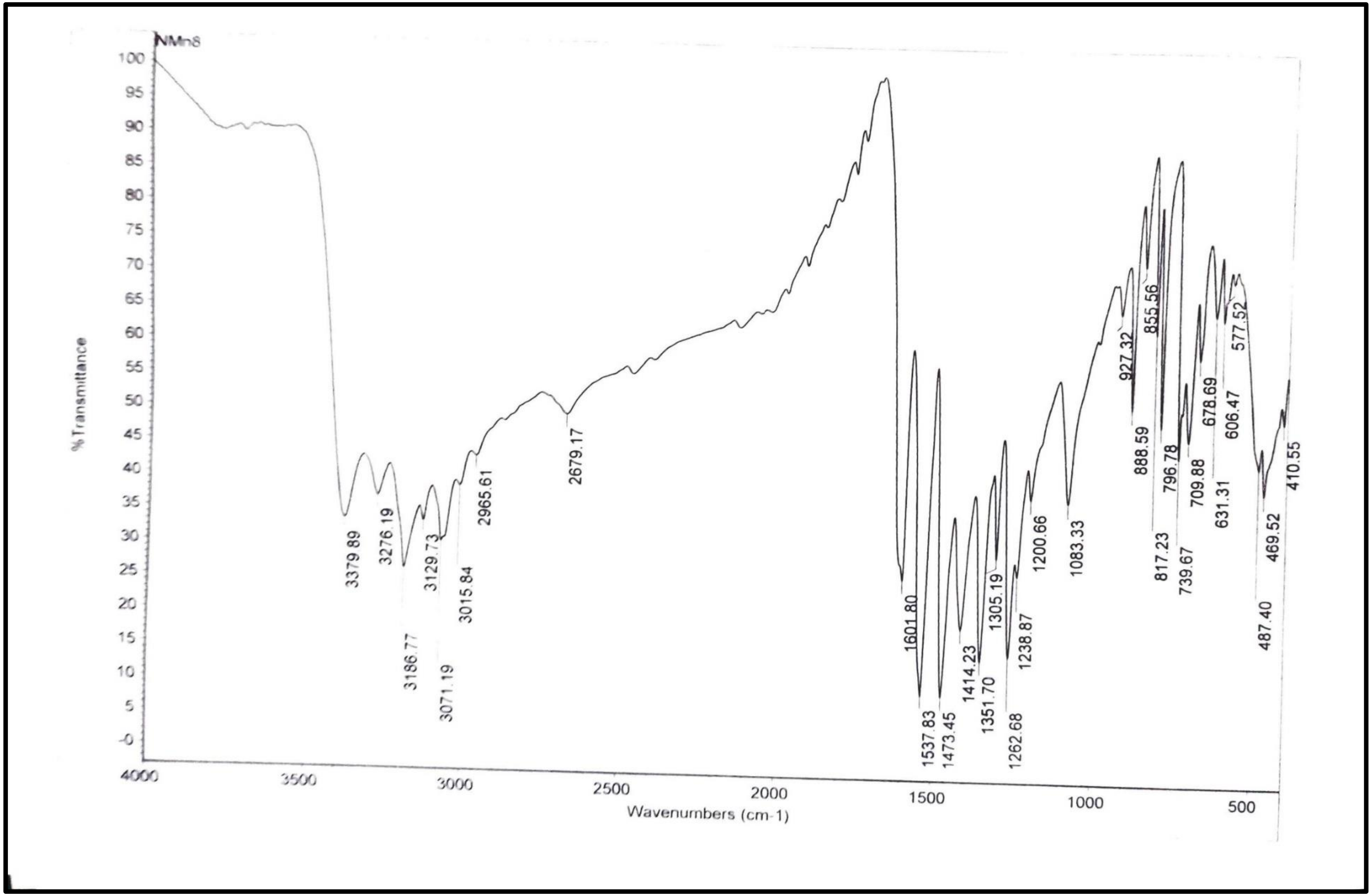

Figure S33: ^{1}H NMR spectrum of compound 9

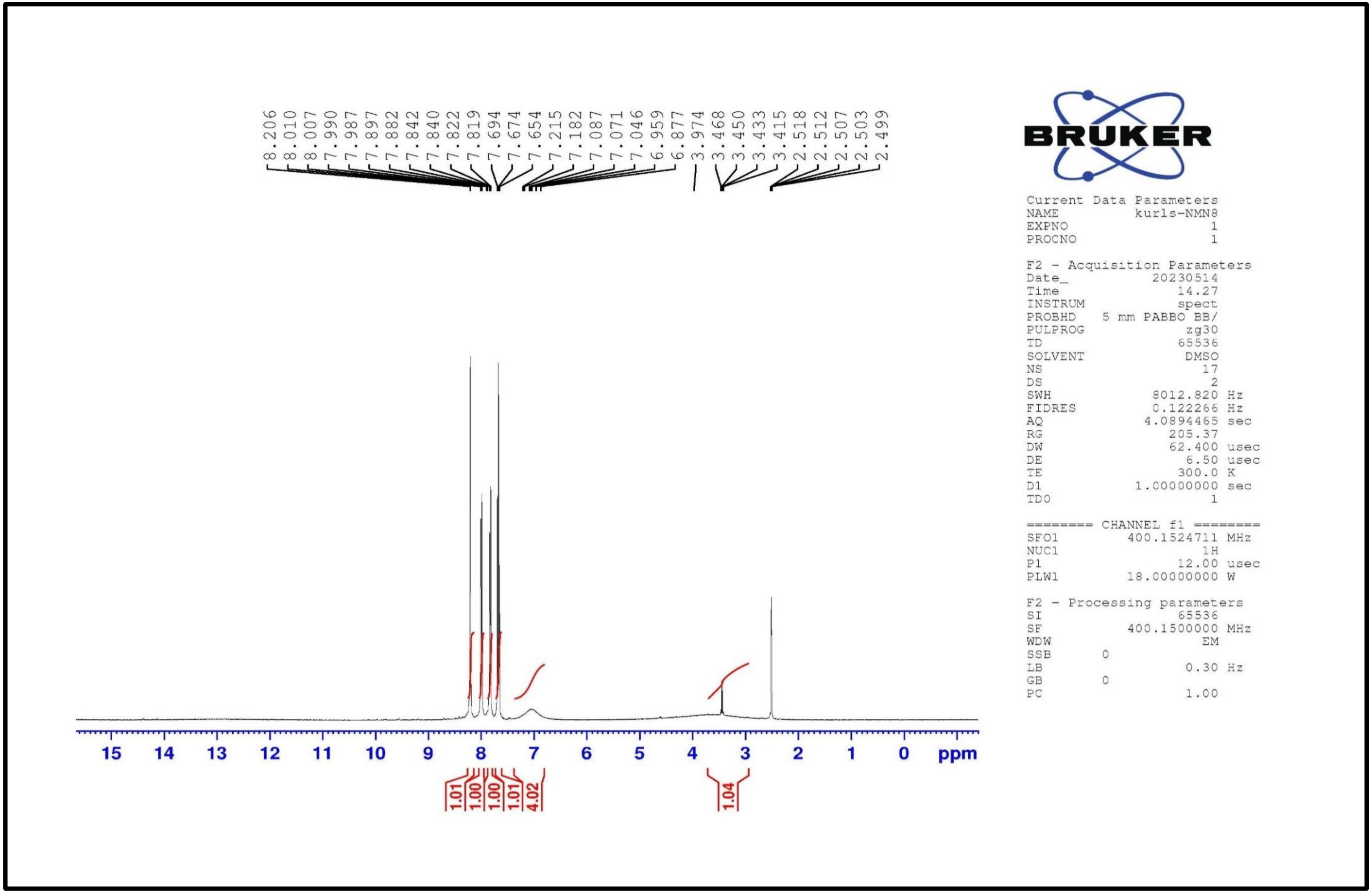

Figure S34: Expanded ^{1}H NMR spectrum of compound 9

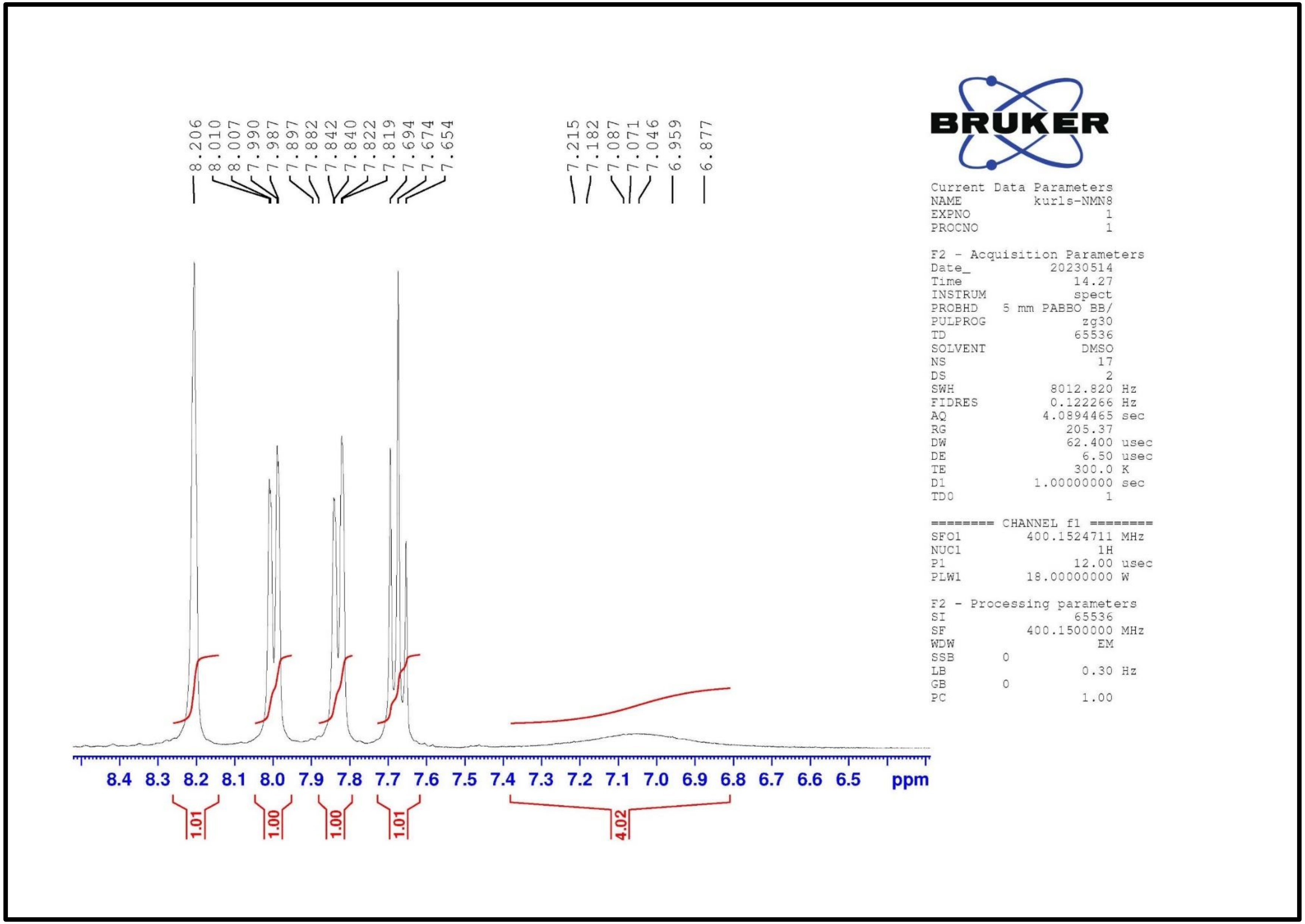

Figure S35: ^{13}C NMR spectrum of compound 9

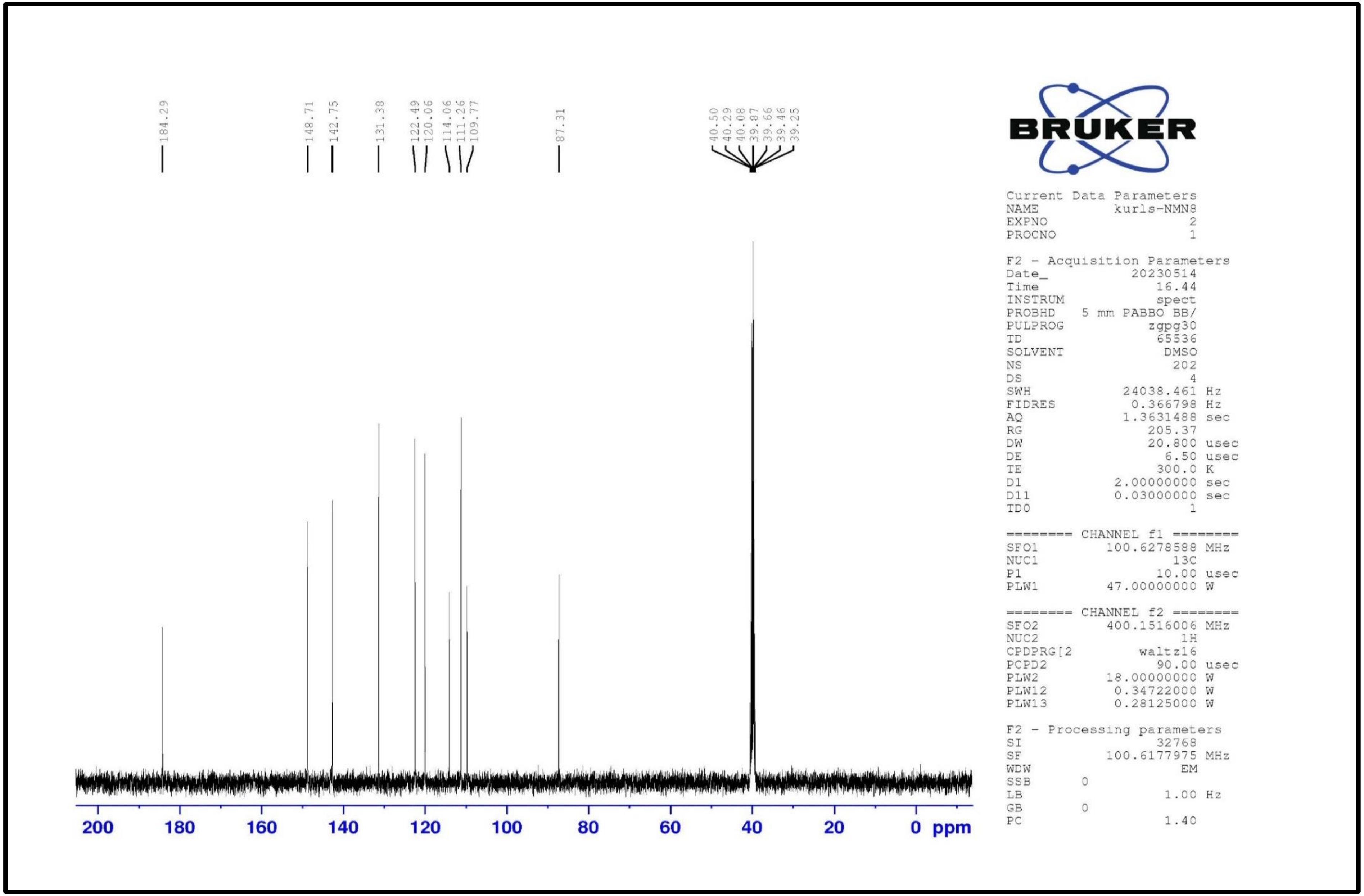

Figure S36: Mass spectrum of compound 9

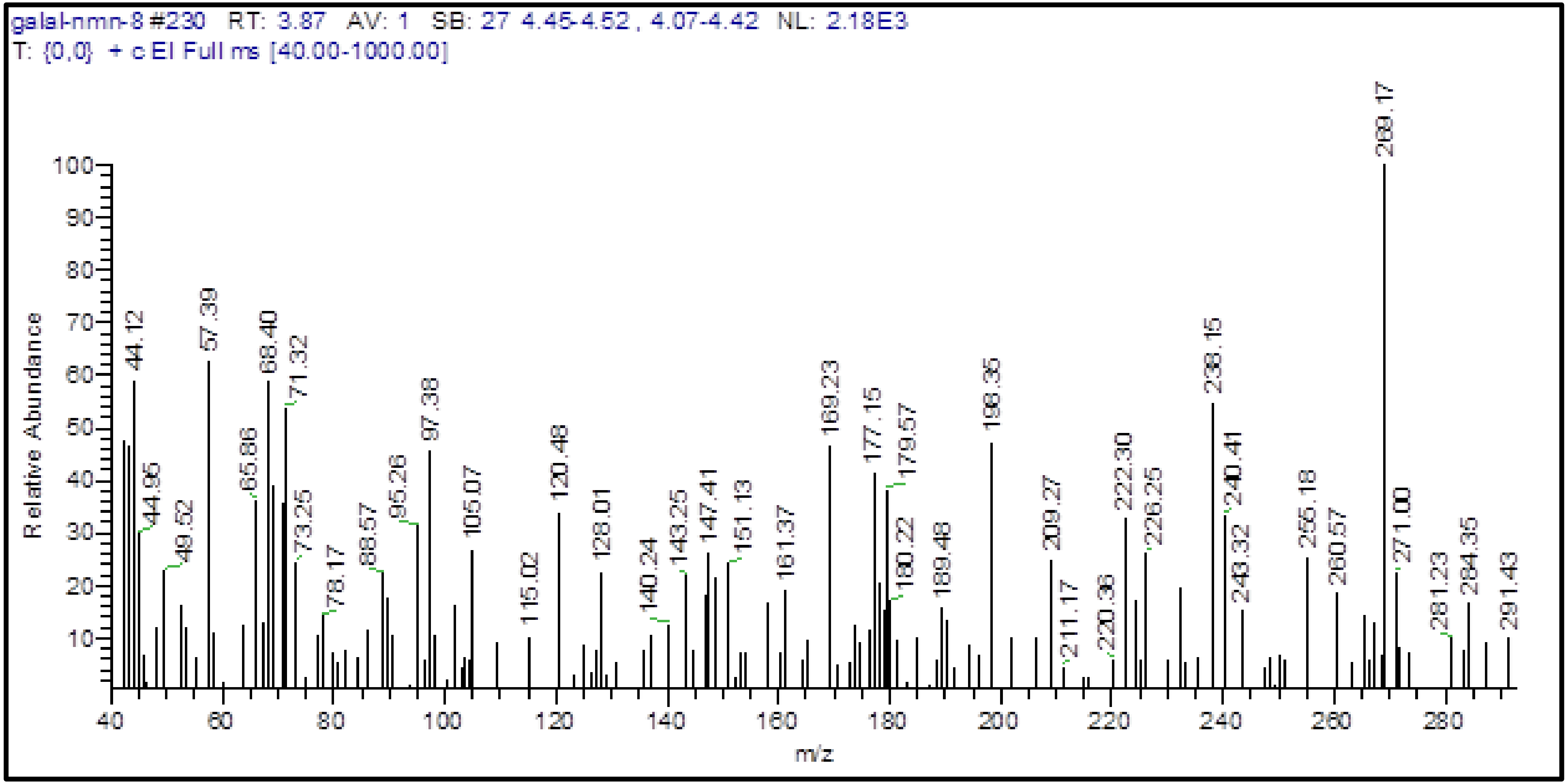

Figure S37: IR spectrum of compound 10

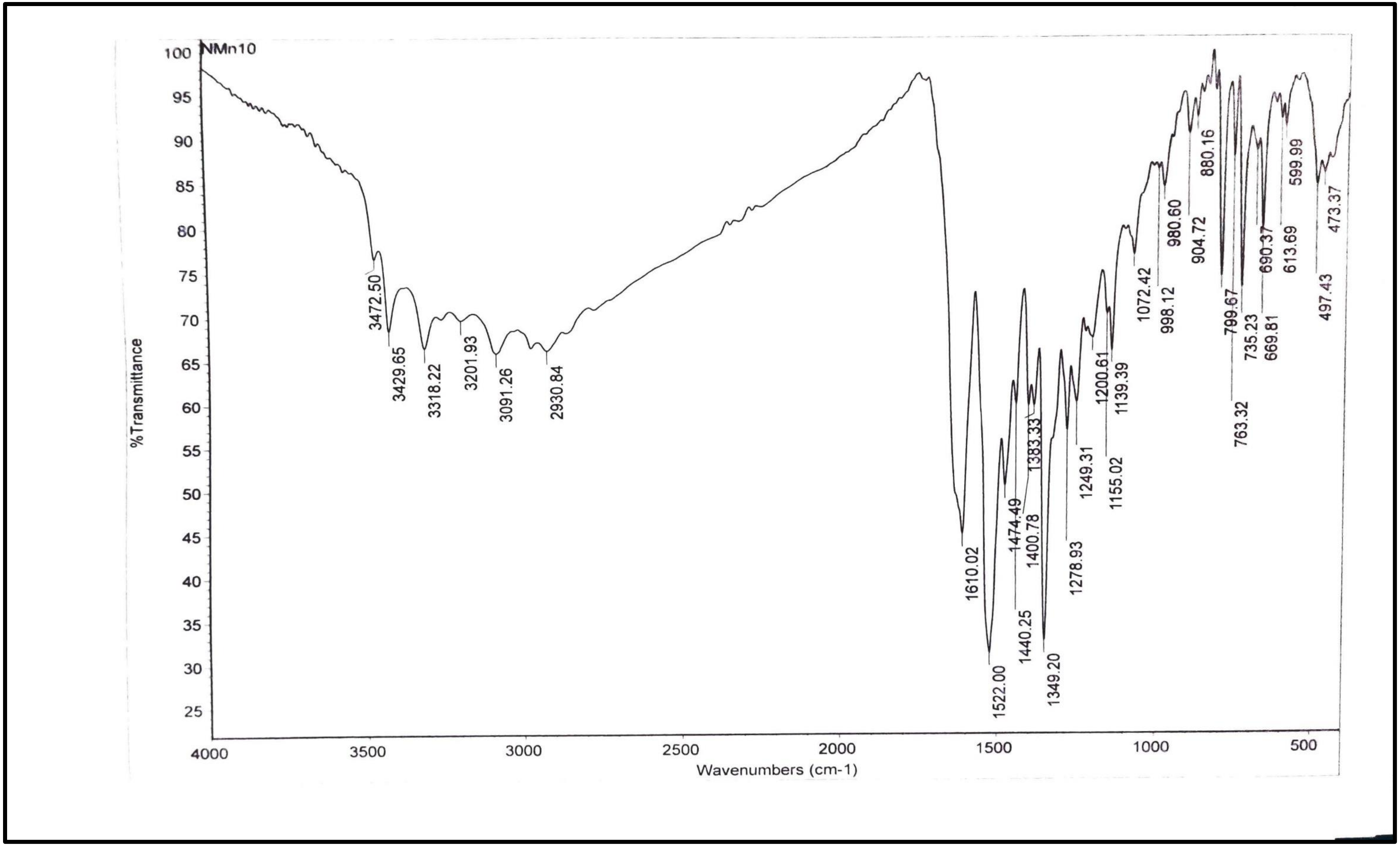

Figure S38: ^{1}H NMR spectrum of compound 10

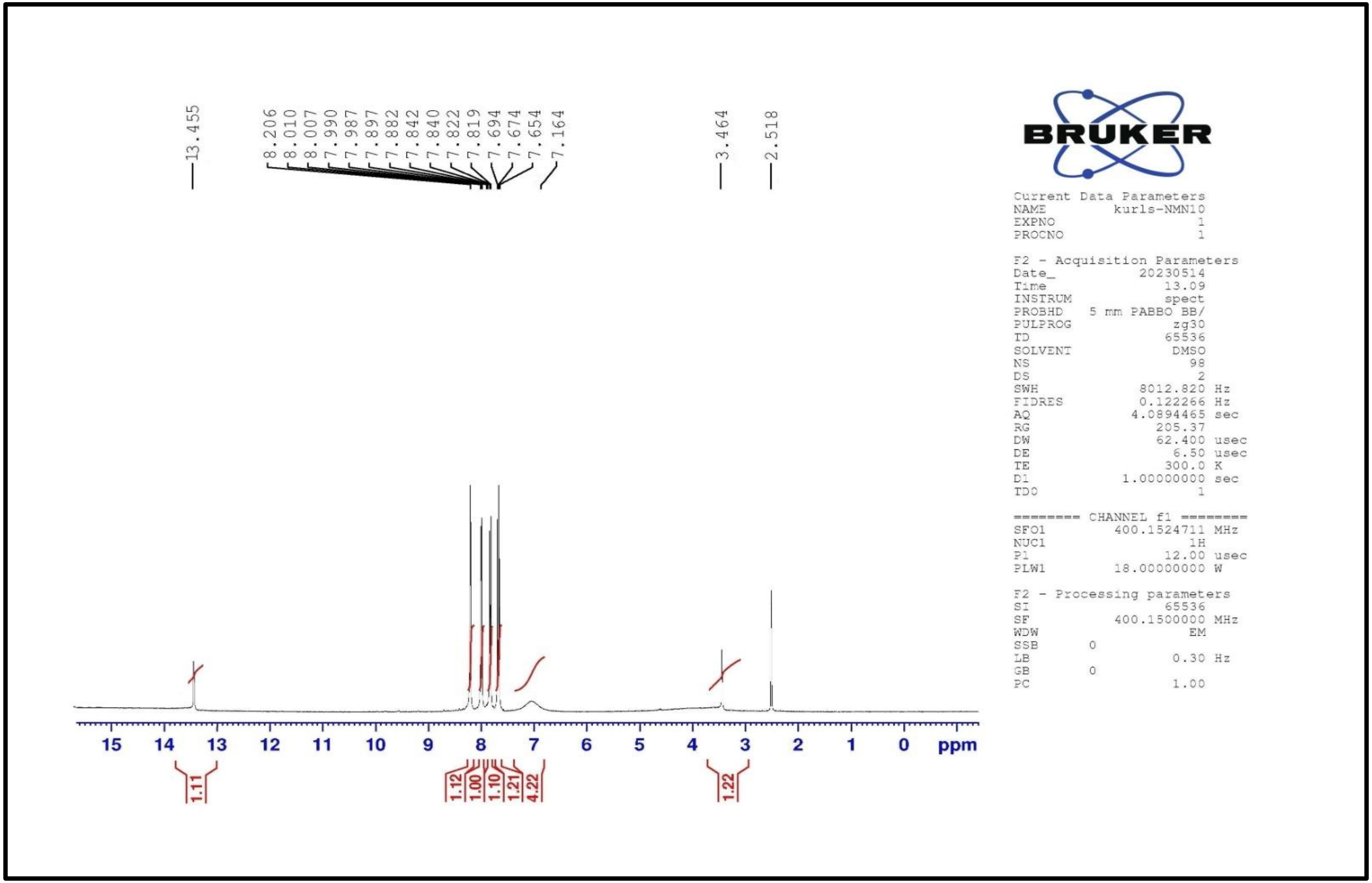

Figure S39: ^{13}C NMR spectrum of compound 10

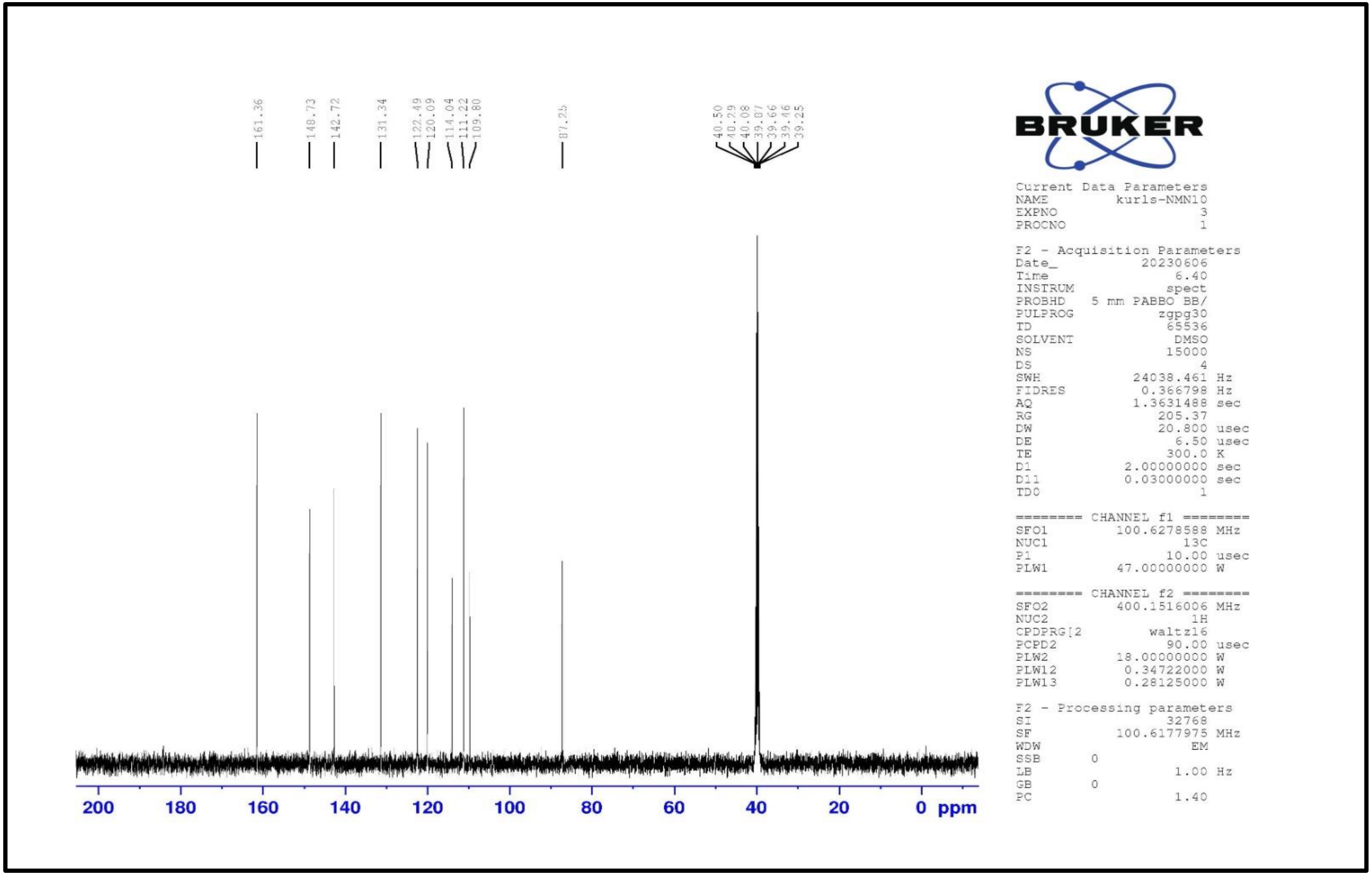

Figure S40: Mass spectrum of compound 10

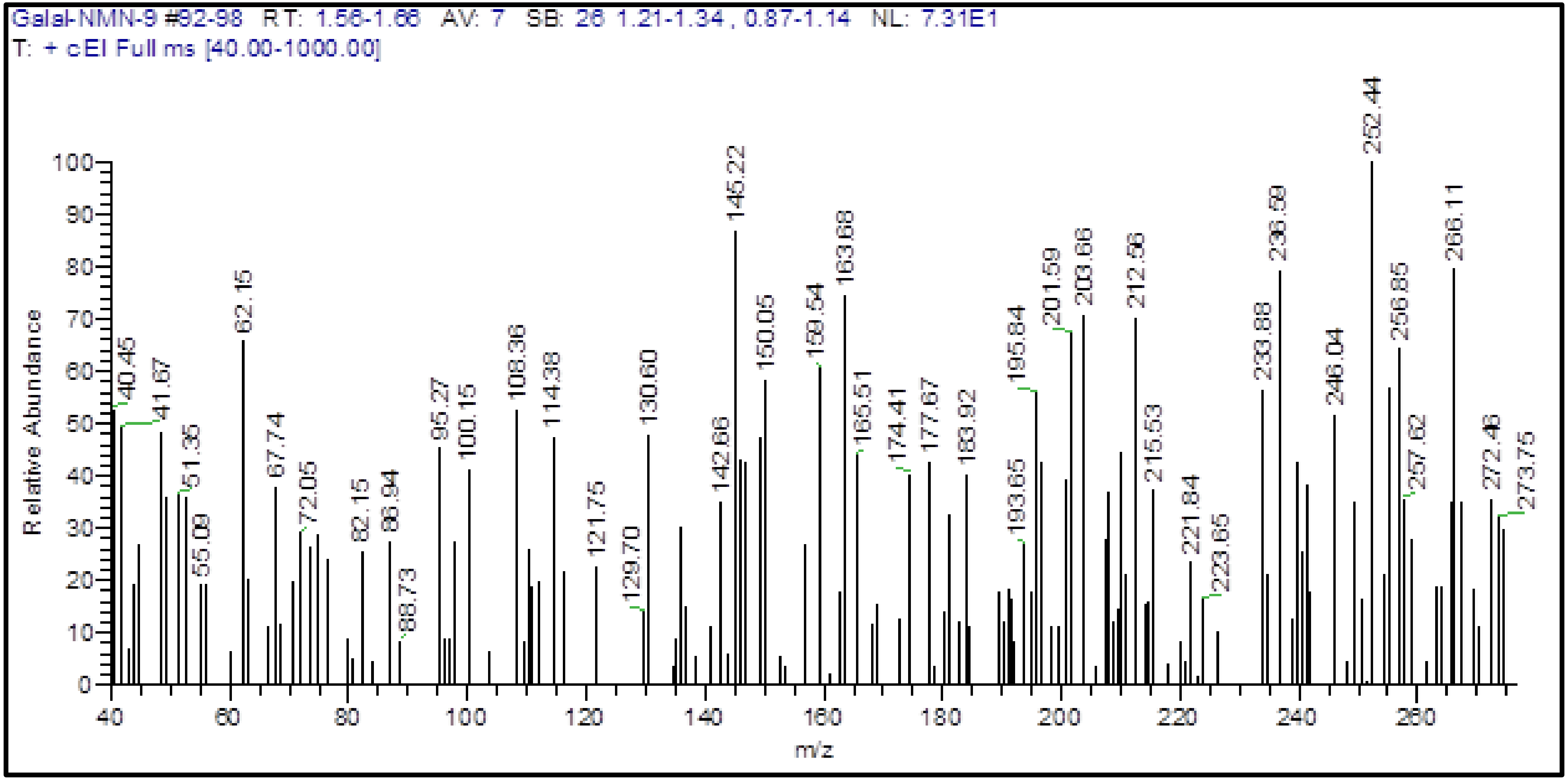

Figure S41: IR spectrum of compound 11

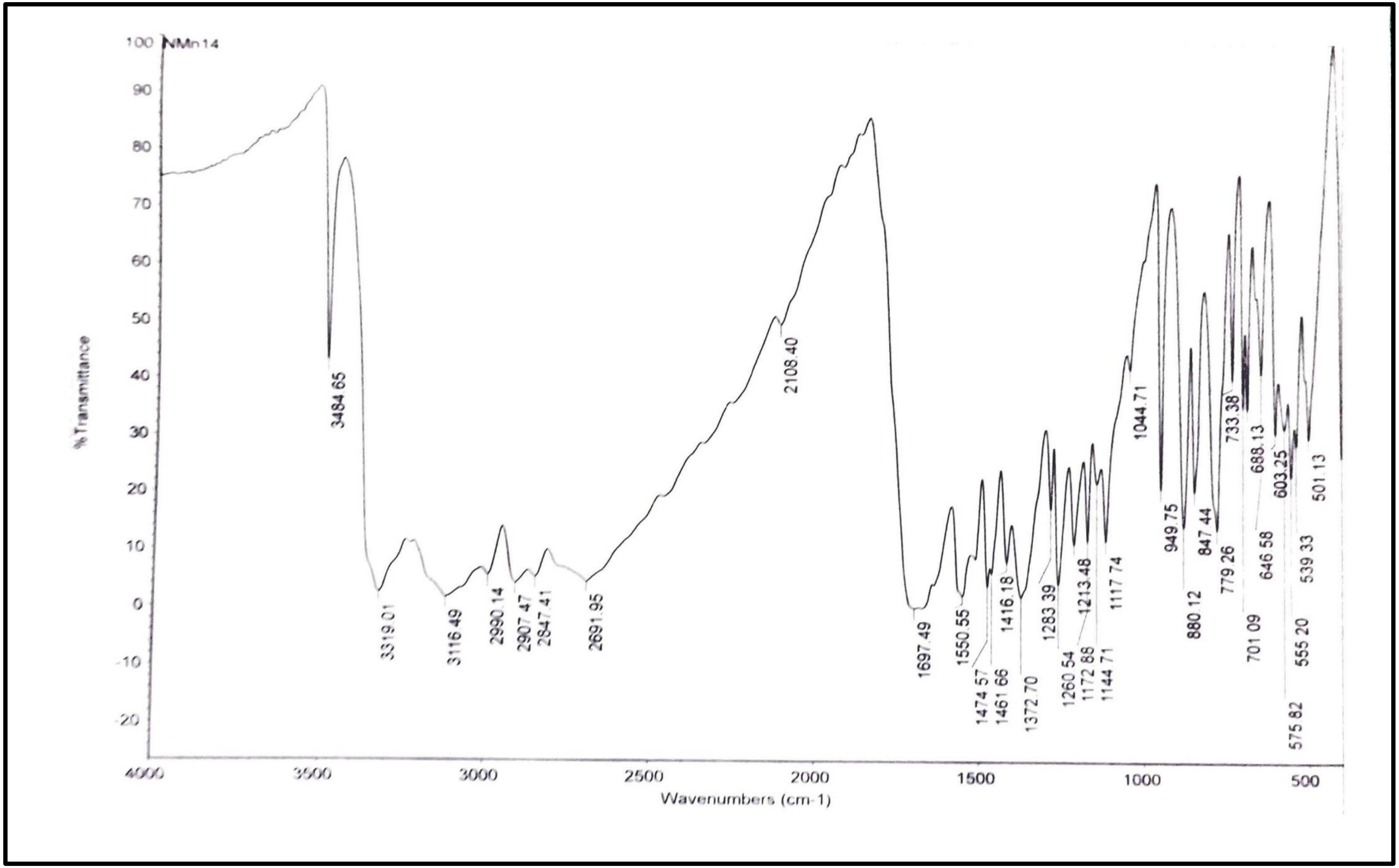

Figure S42: ^{1}H NMR spectrum of compound 11

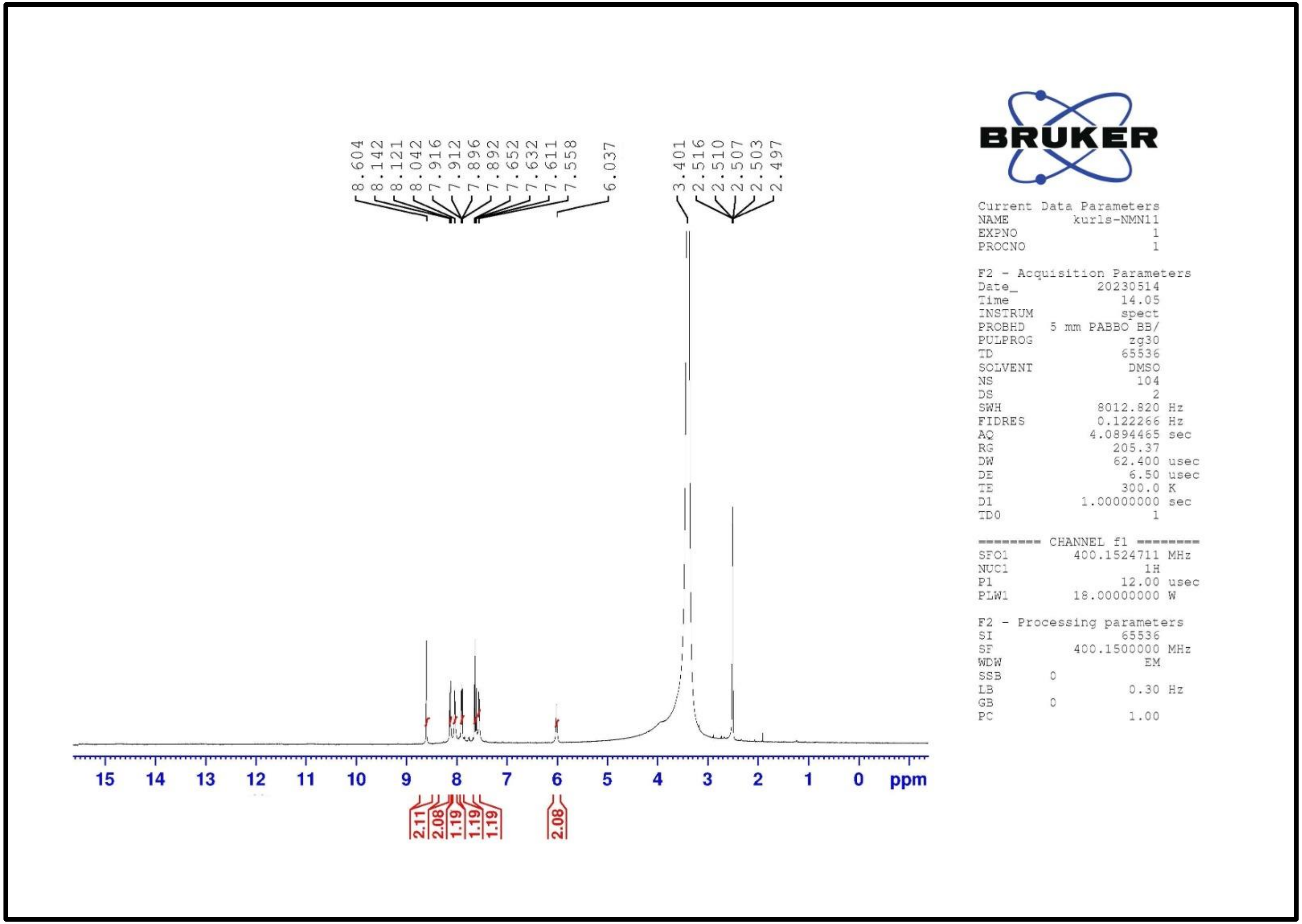

Figure S43: ^{13}C NMR spectrum of compound 11

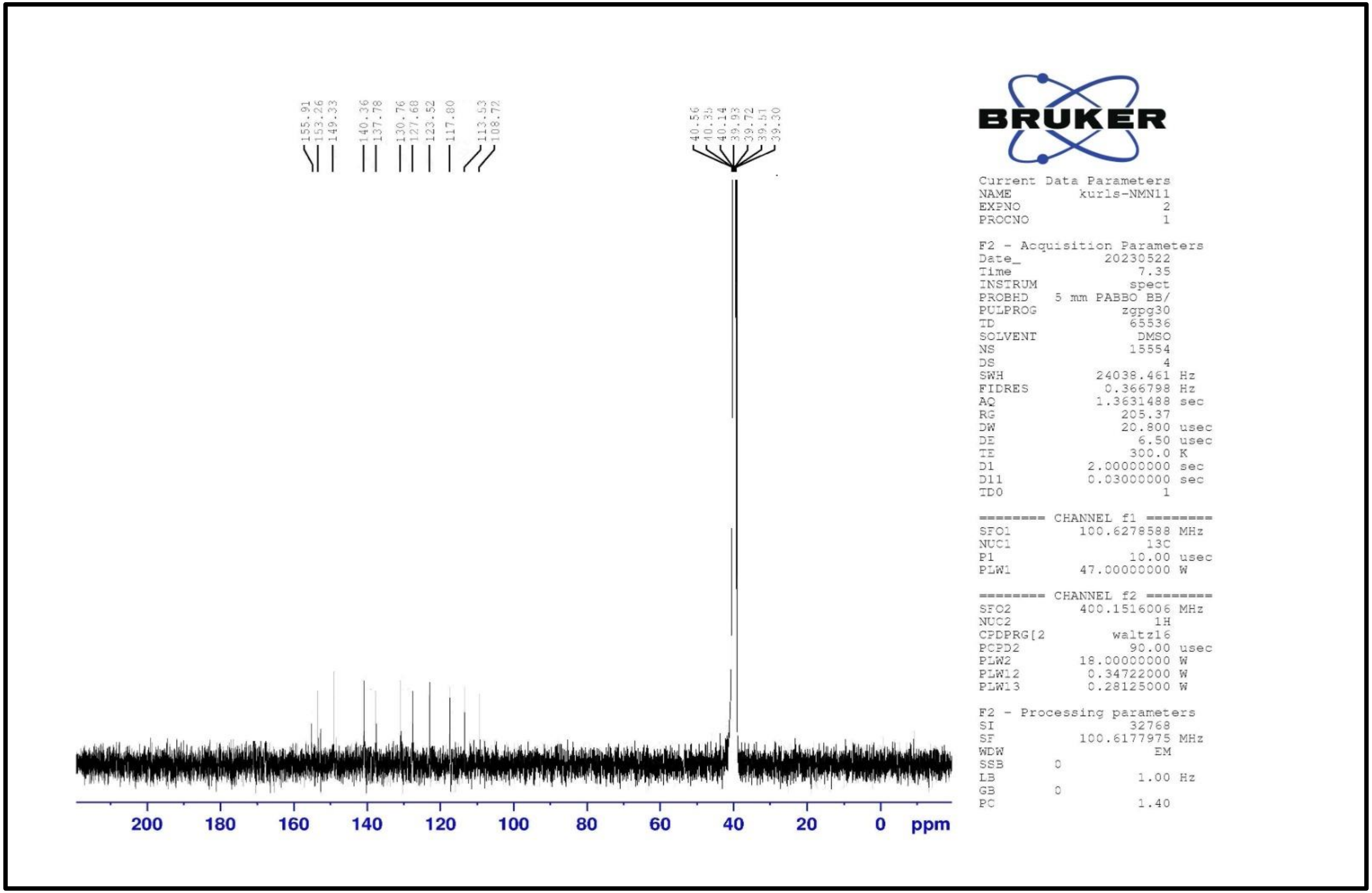

Figure S44: Mass spectrum of compound 11

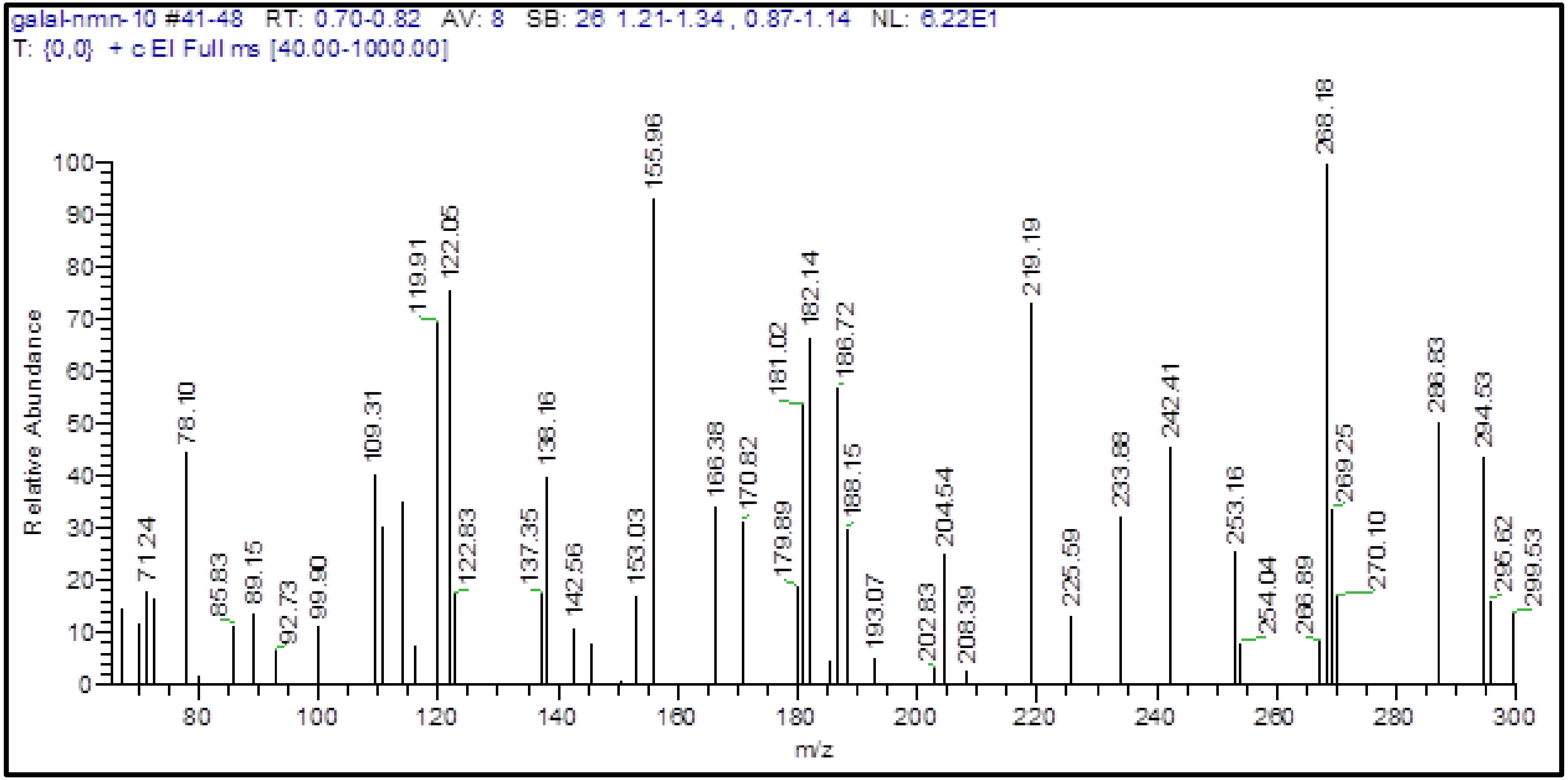

Figure S45: ^{1}H NMR spectrum of compound 12

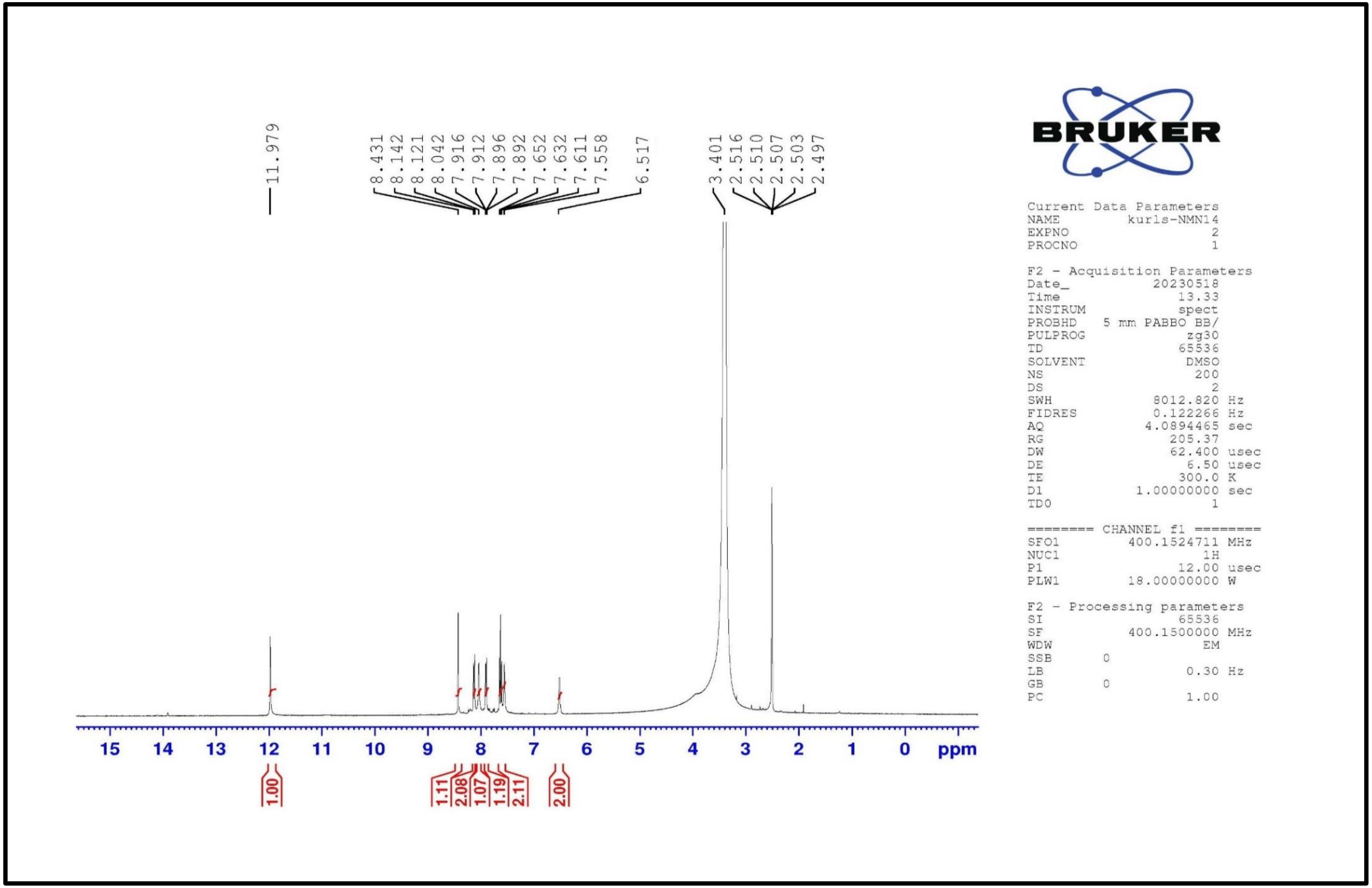

Figure S46: D_2O-^{1}H NMR spectrum of compound 12

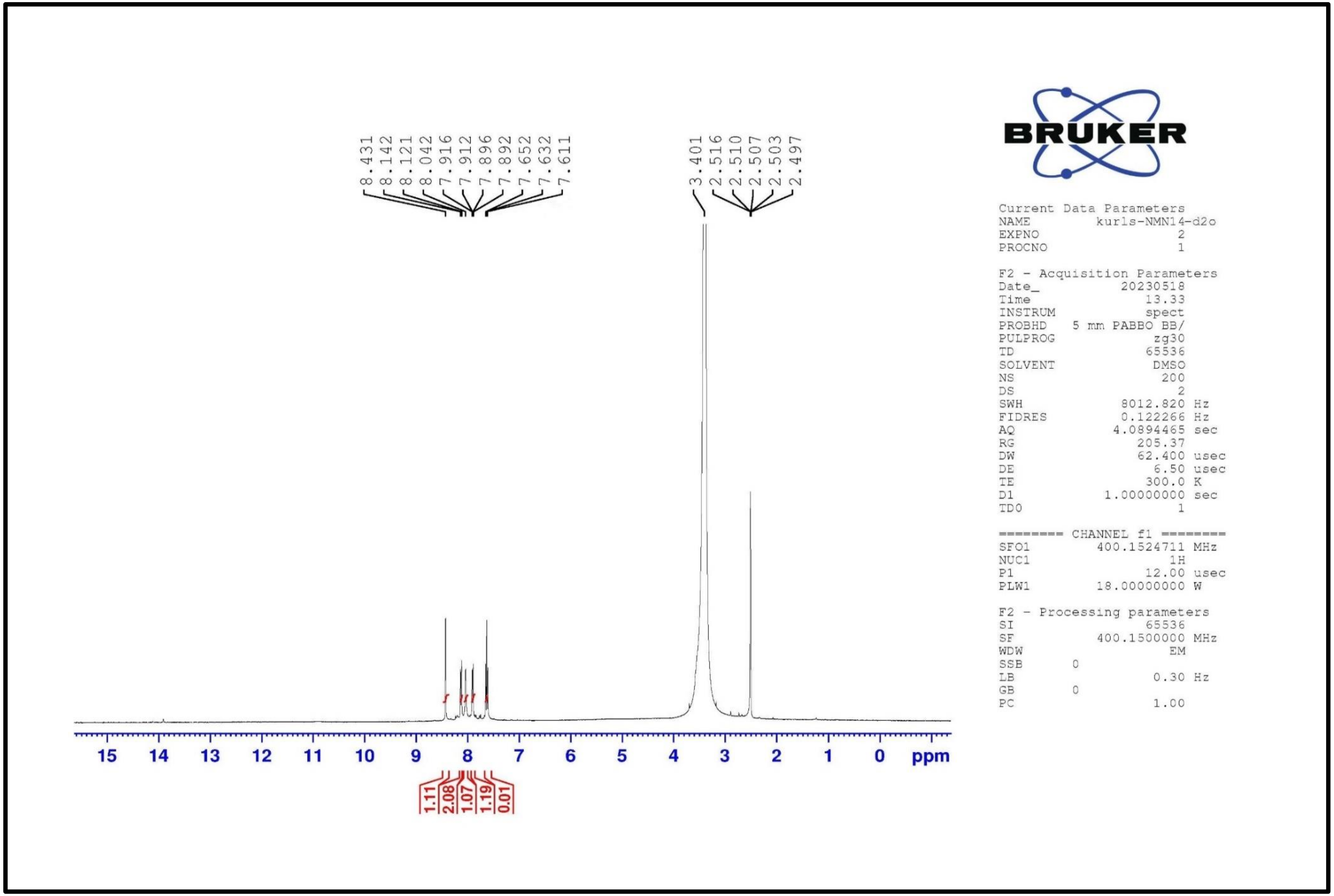

Figure S47: ^{13}C NMR spectrum of compound 12

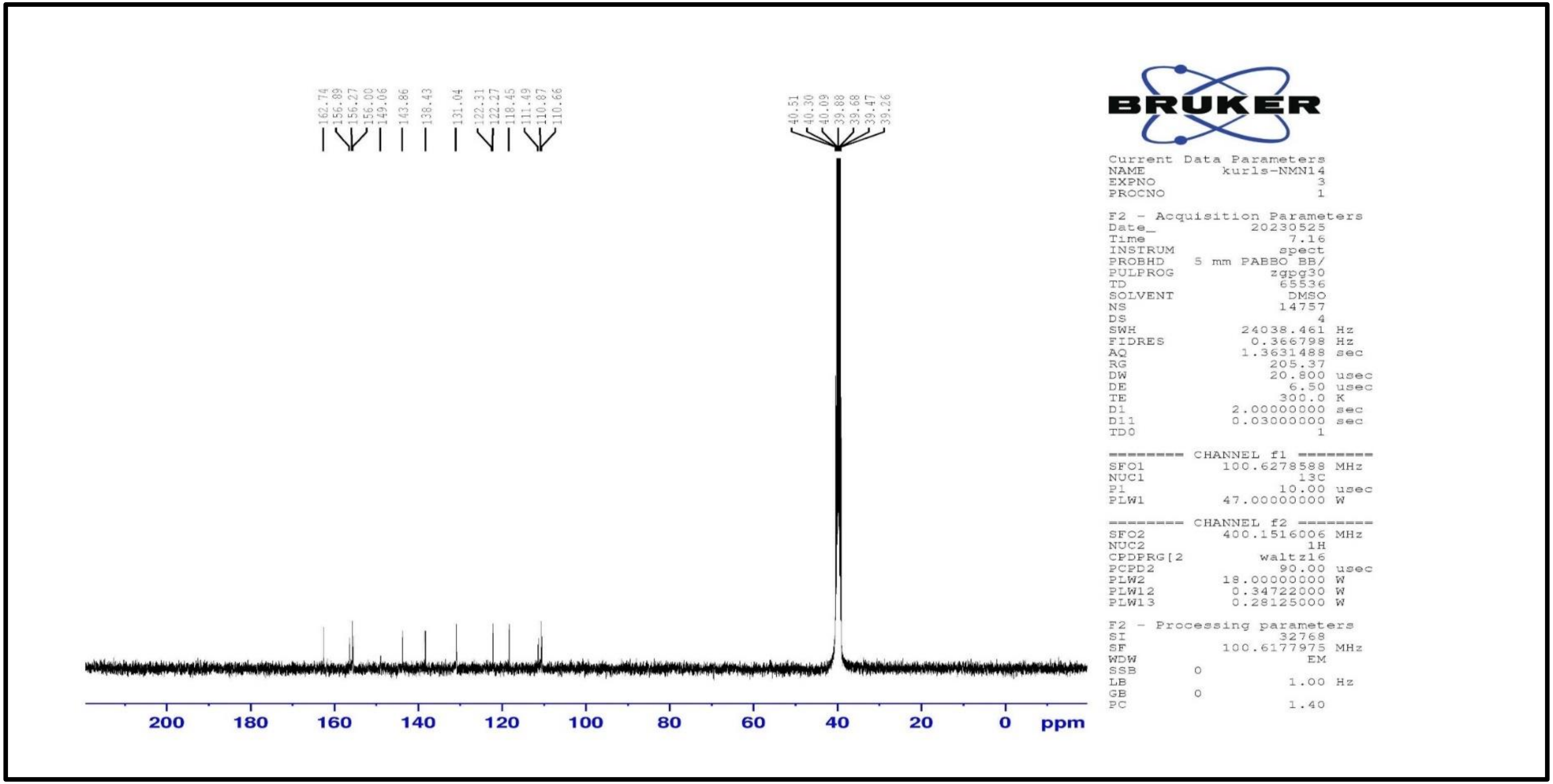

Figure S48: Mass spectrum of compound 12

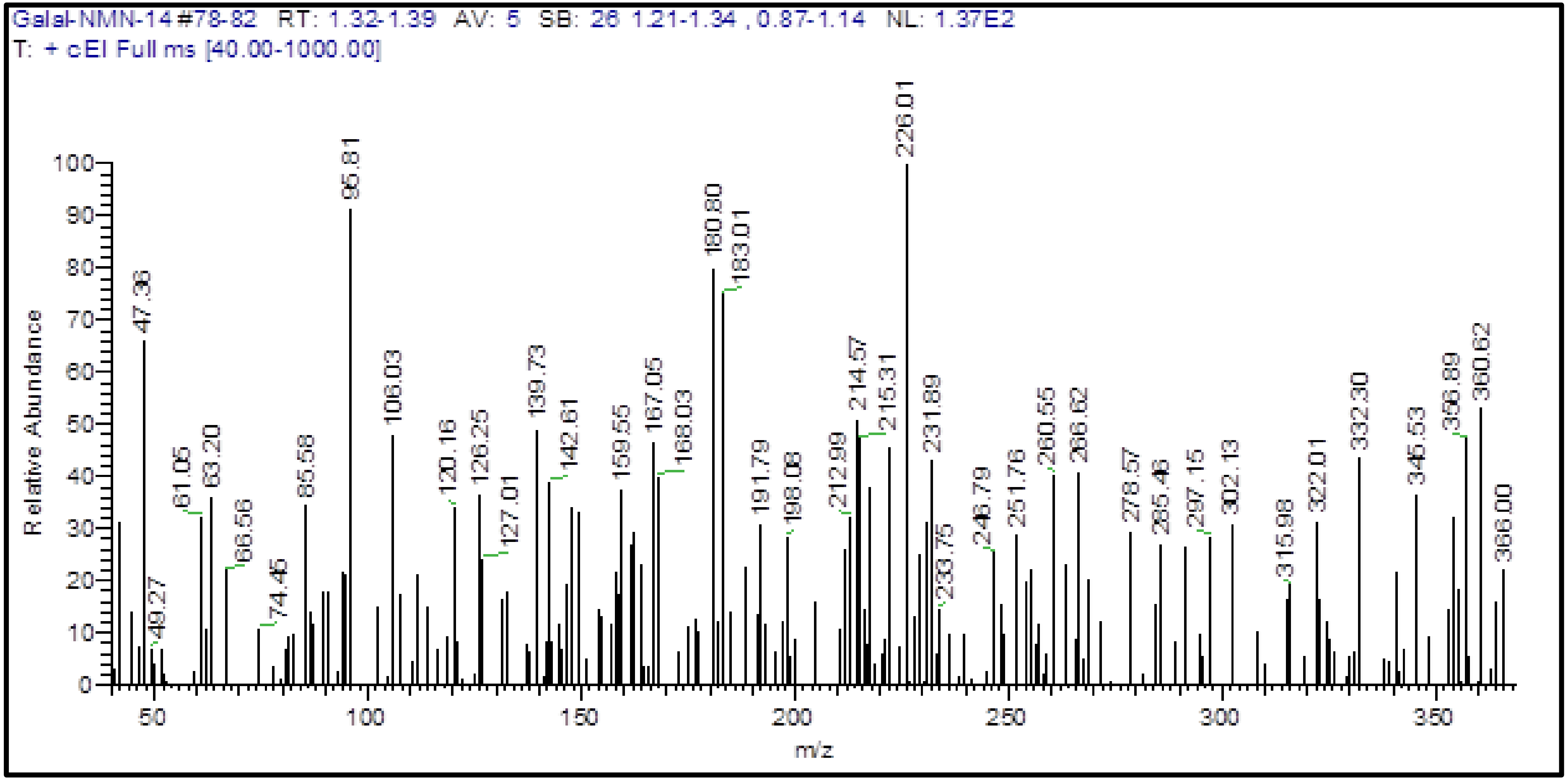

Supplementary S1

4. EXPERIMENTAL

General details:

All starting materials, chemicals, reagents, and solvents were pushed from Sigma Aldrich, all the used solvents were dried via the laboratory chemicals purification handbook. To track the development of the reactions and controlling the homogeneity of the newly prepared compounds, TLC was performed through precoated plates of the silica gel "Merck Kiesel gel 60F254, BDH, Germany". Melting points of all newly prepared compounds were measured on the digital electric Stuart apparatus (SMP3, United Kingdom) for melting point. Microwave irradiation reactions were carried out via Anton Paar microwave reactor (monowave 300, Germany) using 10 mL vials of borosilicate glass. Infrared spectra (IR, cm^{-1}, Shimadzu, Japan) were recorded using KBr disks by PerkinElmer 293 spectrophotometer. ^{1}H and ^{13}C NMR spectrum were determined on Varian Mercury Spectrometer (300 MHz, Shimadzu, Japan) in solvent DMSO-d_6 with an internal standard tetramethyl silane TMS. Multiplicity is symbolized as m "multiple", q "quartet", t "triplet", d "doublet", s “singlet” or combinations therefrom. Coupling constants were measured (J) in Hz and chemical shift (δ) in ppm. Mass spectra were measured using the electrons ionization technique on a Shimadzu Gas chromatography (GC-2010, 70 eV, Japan) instrument mass spectrometer. Elemental microanalyses were recorded on a PerkinElmer analyzer (CHN-2400, United Kingdom) and the good agreement microanalyses within ± 0.4% of the theoretical values. The starting material 1 was prepared by literature known procedure.

Biological evaluation methods

1- Cytotoxicity assay

A- MCF-7 cell line assay

Mammalian cell lines: MCF-7 cells (human breast cancer cell line) were obtained from the American Type Culture Collection (ATCC, Rockville, MD).

Chemicals Used: Dimethyl sulfoxide (DMSO), MTT and trypan blue dye were purchased from Sigma (St. Louis, Mo., USA).

Fetal Bovine serum, RPMI-1640, HEPES buffer solution, L-glutamine, gentamycin and 0.25% Trypsin-EDTA were purchased from Lonza (Belgium).

- Cell line Propagation:

The cells were grown on RPMI-1640 medium supplemented with 10% inactivated fetal calf serum and 50µg/ml gentamycin. The cells were maintained at 37ºC in a humidified atmosphere with 5% CO2 and were sub-cultured two to three times a week.

- Cytotoxicity evaluation using viability assay:

For antitumor assays, the tumor cell lines were suspended in medium at concentration $5x10^4$ cell/well in Corning® 96-well tissue culture plates, then incubated for 24 hr. The tested compounds were then added into 96-well plates (three replicates) to achieve twelve concentrations for each compound. Six vehicle controls with media or 0.5 % DMSO were run for each 96 well plate as a control. After incubating for 24 h, the numbers of viable cells were determined by the MTT test. Briefly, the media was removed from the 96 well plate and replaced with 100 µl of fresh culture RPMI 1640 medium without phenol red then 10 µl of the 12 mM MTT stock solution (5 mg of MTT in 1 mL of PBS) to each well including the untreated controls. The 96 well plates were then incubated at 37°C and 5% CO_2 for 4 hours. An 85 µl aliquot of the media was removed from the wells, and 50 µl of DMSO was added to each well and mixed thoroughly with the pipette and incubated at 37°C for 10 min. Then, the optical density was measured at 590 nm with the microplate reader (SunRise, TECAN, Inc, USA) to determine the number of viable cells and the percentage of viability was calculated as [(ODt/ODc)]x100% where ODt is the mean optical density of wells treated with the tested sample and ODc is the mean optical density of untreated cells. The relation between surviving cells and drug concentration is plotted to get the survival curve of each tumor cell line after treatment with the specified compound. The 50% inhibitory concentration (IC_{50}), the concentration required to cause toxic effects in 50% of intact cells, was estimated from graphic plots of the dose response curve for each conc. using Graphpad Prism software (San Diego, CA. USA) (Mosmann, 1983).

B- MDA-MB-231 cell line assay

Materials and methods

- **Cell line**

Breast cancer (MDA-MB-231). The cell line was obtained from ATCC via Holding company for biological products and vaccines (VACSERA), Cairo, Egypt. Doxorubicin was used as a standard anticancer drug for comparison.

- **Chemical reagents**

The reagents RPMI-1640 medium , MTT and DMSO (sigma co., St. Louis, USA), Fetal Bovine serum (GIBCO, UK) .

- **MTT assay**

The cell lines mentioned above were used to determine the inhibitory effects of compounds on cell growth using the MTT assay. This colorimetric assay is based on the conversion of the yellow tetrazolium bromide (MTT) to a purple formazan derivative by mitochondrial succinate dehydrogenase in viable cells. Cell lines were cultured in RPMI-1640 medium with 10% fetal bovine serum. Antibiotics added were 100 units/ml penicillin and 100µg/ml streptomycin at 37 C in a 5% Co2 incubator. The cell lines were seedes in a 96-well plate at a density of 1.0x104 cells/well. at 37 C for 48 h under 5% Co2. After incubation the cells were treated with different concentration of compounds and incubated for 24 h. After 24 h of drug treatment, 20 µl of MTT solution at 5mg/ml was added and incubated for 4 h. Dimethyl sulfoxide (DMSO) in volume of 100 µl is added into each well to dissolve the purple formazan formed. The colorimetric assay is measured and recorded at absorbance of 570 nm using a plate reader (EXL 800 , USA). The relative cell viability in percentage was calculated as (A570 of treated samples/A570 of untreated sample) X 100.

2- HSP90 inhibitory activity

In vitro antiproliferative activity by the cell viability assay Antiproliferative potency of two compounds (C9 and C10) on breast cancer cell line, MDA-MB-231, was measured by the MTT cell viability test and results were estimated in terms of IC50 values. Moreover, two compounds showed growth inhibition on MDA-MB-231 cell. C9 and C10 manifested cytotoxicity with IC_{50} values of 8.907±0.287 µM and 10.643±0.463 µM on MDA-MB-231 respectively.

- **Cell Line**

The Cell lines were used in the study is TNBC (MDA-MB-231) cell line, they were purchased from the American Type Culture Collection (ATCC, USA) to evaluate the effects of the examined drugs.

- **Drugs**

C9 and C10 on MDA-MB-231: These compounds were dissolved in dimethyl sulphoxide (DMSO) in a concentration of 8.907 µM and 10.643µM.

- **Cell culture material and chemicals**

1. Phenol red-free Dulbecco's modified Eagle's medium (DMEM) was purchased from Lonza® (Basel, Switzerland).
2. Fetal bovine serum (FBS) (Sigma-Aldrich Co., USA), store at −20°C.
3. Trypsin (Sigma-Aldrich Co., USA).
4. DMSO (Sigma-Aldrich Co., USA).
5. Phosphate Buffer Saline (PBS) (Lonza®, Basel, Switzerland).
6. Antibiotics: Penicillin and Streptomycin (Lonza®, Basel, Switzerland).
7. RIPA lysis and extraction buffer, purchased from Thermo Scientific, USA (#89900)
8. 6-well plates (Sigma-Aldrich Co, USA).
9. T25 and T75 tissue culture flasks with filtered cap (Thermo Fisher Scientific Inc., MA, USA).
10. 15 ml and 50 ml falcon centrifuge tubes (Thermo Fisher Scientific Inc., MA, USA).
11. 3-(4, 5- dimethylthiazolyl-2)-2, 5-diphenyltetrazolium bromide (MTT) (Sigma-Aldrich Co., USA).
12. Ethanol was bought from El-Nasr Pharmaceutical Chemicals Co. (Cairo, Egypt).

- **Methods**
- **Cell cultures**

MDA-MB231 cell was maintained as a monolayer culture in T-25 flasks at 37°C. The cell was cultured in DMEM supplemented with 10% FBS, 100U/ml penicillin, and 100µg/ml streptomycin and incubated in a 37° C in humidified air containing 5% carbon dioxide.

- **Cell Storage**

MDA-MB231 cell was aliquoted at 2×10^6 viable cells/ ml in a cryomedium 80% (v/v) DMEM, 10% (v/v) FBS and 10% (v/v) DMSO. The cryovials were stored overnight at -80°C, and then transferred to a liquid nitrogen tank for long term storage.

- **Cell thawing**

A cryovial was quickly thawed by gentle agitation in 37°C water bath and transferred into a laminar flow hood. Thawed cells were transferred to a 50ml sterile falcon tube containing 50 ml of prewarmed 90% (v/v) DMEM supplemented with 10% (v/v) FBS (complete growth medium). Cell suspension was centrifuged at 200 × g for 5 min. Cells were maintained at 37°C in a humidified 5% CO_2 atmosphere. Cells were sub-cultured every 48 h at 70% confluency and visualized using an inverted microscope.

- **Cell subculture**

MDA-MB231 cell was passaged when they were 70% confluent, about every two days. Media were removed by aspiration and 5 ml of PBS pH 7.2 were added to wash the media from adherent cells. To detach the adherent cells, 2 ml of 0.25% (w/v) Trypsin were added to

the T-25 flasks and cells were incubated for 5 min at 37ºC. The T-25 flasks were tapped gently against the palm to detach cells, and cells were observed under the inverted microscope (Micro master inverted digital microscope, Thermo Fisher Scientific Inc., USA) every 2-3 min.

After 5-10 min of incubation, cells were detached and slided down the surface when the flask was tilted. The trypsin cell suspension was neutralized by adding an equal volume of complete growth media to the T-25 flask. Cells were dispersed by pipetting gently over the surface of the monolayer. The cell suspension was pipetted up and down with the tip of the pipette resting on the bottom corner or edge until a single cell suspension was obtained. The cell suspension was then transferred to 15 ml falcon tube to which 5 ml complete growth media were added. Falcon tubes were centrifuged at 12,000 rpm at 4ºC for 5 min. The supernatant was removed by aspiration, and cells were resuspended in 5 ml of complete media. Following resuspension, the cell suspension was transferred into new pre-labeled T-25 flasks at a seeding density 4 x 10^4 viable cells/cm^2. The flasks were then incubated at 37ºC in 5% CO_2 to allow cell attachment.

- **Cell Counting**

To determine an inoculum with the appropriate cell concentration for seeding, cells were counted using the hemocytometer, a graduated counting chamber for determining the concentration of cells in a suspension. Cell suspension (10 µl) was mixed with equal volume of trypan blue and loaded in both chambers. Unstained cells (viable cells) were counted under an inverted microscope at 10x magnification. The total number of cells can be calculated as follows:

Cells/ml = Total Cells counted in the Hemocytometer Set of Square $\times 4 \times 10^4 \times$ Dilution Factor.

- **Assays**

MTT Cell Proliferation Assay (*In-Vitro* Cytotoxicity Assay)

Cytotoxicity was determined by classic Microculture tetrazolium test (MTT). The MTT cell proliferation assay measures the cell proliferation. In principle, the yellow tetrazolium MTT (3-(4, 5-dimethylthiazolyl-2)-2, 5-diphenyltetrazolium bromide) is reduced by metabolically active cells, yielding purple MTT formazan crystals which has a λ max of 540 nm and is considered to be directly proportional to the number of viable cells.

- **Determination of HSP90**

Using Human HSP90 Sandwich ELISA Kit (Catalog No.: KE00054). This ELISA kit applies to the quantitative determination of Human Hsp90 concentrations.

- **Principle of assay:**

HSP90 provides chaperoning activity for client proteins; many of them are members of oncogenic pathways, indicating its implication in tumor malignancy. HSP90 mainly resides

in the cytosol, while it can also be released to the extra-cellular space. Secreted Hsp90 is a C-terminal truncated form. It has been reported that the level of plasma Hsp90 is positively correlated with tumor malignancy in clinical cancer patients, and can be a promising diagnostic marker for tumor malignancy in clinical application.

Sandwich ELISA structure (HRP conjugated secondary antibody) a capture antibody is pre-coated onto the bottom of wells which binds to analyte of interest. A detection antibody also binds to the analyte. Horseradish peroxidase (HRP)-conjugated secondary antibody binds to the detection antibody. TMB acts as the HRP substrate and the solution color will change from colorless to blue. A stop solution containing sulfuric acid turns solution yellow. The color intensity is proportional to the quantity of bound protein which is measurable at 450 nm.

- **Kit components:**

Table : Components of Human Caspase-3 ELISA Kit

**Microplate** - antibody coated 96-well microplate (8 well × 12 strips)	1 plate	**Unopened Kit:** Store at 2-8°C for 6 months or -20°C for 12 months. **Opened Kit:** All reagents stored at 2-8°C for 7 days. **Please use a new standard for each assay.**
**Protein standard** - 16000 pg/bottle; lyophilized	2 bottles	
**Detection antibody (100×)** - 120 µL/vial*	1 vial	
**HRP-conjugated antibody (100×)** - 120 µL/vial*	1 vial	
**Sample Diluent PT 1-ef** - 30 mL/bottle. For Human serum, plasma, cell culture supernatant and urine samples	1 bottle	
**Sample Diluent PT 5-ef** - 30 mL/bottle. For cell lysate samples	1 bottle	
**Detection Diluent** - 30 mL/bottle	1 bottle	
**Wash Buffer Concentrate (20×)** - 30 mL/bottle	1 bottle	
**Extraction Reagent** - 30 mL/bottle	1 bottle	
**Tetramethylbenzidine Substrate (TMB)** - 12 mL/bottle	1 bottle	
**Stop Solution** - 12 mL/bottle	1 bottle	
**Plate Cover Seals**	4 pieces	

* Centrifugation immediately before use

- **Sample preparation**
  1- Collect cells and wash by centrifuging at 500 xg for 5 minutes before resuspension in pre-cooled PBS buffer.
  2- Count cells and then discard the supernatant.
  3- Add protease inhibitor cocktail to the Extraction Reagent to a final concentration immediately prior to performing cell lysis.
  4- Add 1 mL of Extraction reagent Per 1 x 107 cells
  5- Centrifuge cell lysate at 10,000 xg for 5 minutes at 4℃.
  6- Measure the concentration of total protein in cell lysate.
- **Reagent preparation:**
  1- Wash Buffer (1X): If crystals have formed in the concentrate, warm to room temperature and mix gently until the crystals have completely dissolved. Add 30 mL of Wash Buffer Concentrate (20X) to 570 mL deionized or distilled water to prepare 1X Wash Buffer.
  2- Detection Antibody (1X): Dilute 100X Detection Antibody 1:100 using Detection Diluent prior to assay. Suggested 1:100 dilution: 10 µL 100X Detection Antibody +

990 μL Detection Diluent (Centrifuge the 100 X Detection Antibody solution for a few seconds prior to use).

3- HRP-conjugated antibody (1X): Dilute 100X HRP-conjugated antibody 1:100 using Detection Diluent prior to assay. Suggested 1:100 dilution: 10 μL 100X HRP-conjugated antibody + 990 μL Detection Diluent (Centrifuge the 100X HRP conjugated antibody solution for a few seconds prior to use).

4- Sample Dilution: Different samples should be diluted with corresponding Sample Diluent, samples may require further dilution if the readout values are higher than the highest standard OD reading. Variations in sample collection, processing and storage may affect the results of the measurement. Recommended Dilution for different sample types: 1:2 or 1:4 is recommended for serum and plasma; 1:2 or 1:4 is recommended for cell culture supernatant; 1:2 or 1:4 is recommended for cell lysate; 1:2 or 1:4 is recommended for urine.

- **Assay procedure:**

1- Bring all reagents to room temperature before use (Detection antibody and HRP-conjugated antibody can be used immediately). To avoid cross-contamination, change pipette tips between additions of each standard level, between sample additions, and between reagent additions. Also, use separate reservoirs for each reagent.

2- Take out the required number of microplate strips and return excess strips to the foil pouch containing the drying reagent pack and reseal; store at 4°C immediately. Microplate strips should be used in one week.

3- Preset the layout of the microplate, including control group,standard group and sample group, add 100 μL of each standard and sample to the appropriate wells.(Make sure sample addition is un interrupted and completed within 5 to 10 minutes, It is recommended to assay all standards, controls, and samples in duplicate).

4- Seal plate with coverseal, pressing it firmly onto top of microwells. Incubate the plate for 1 hour at 37°C.

5- Gently remove the coverseal. Discard the liquid from wells by aspirating or decanting. Remove any residual solution by tapping the plate a few times on fresh paper towels.

6- Wash 4 times with 1X Wash Buffer, using at least 350-400 μL per well. Following the last wash, firmly tap plates on fresh towels 10 times to remove residual Wash Buffer. Avoid getting any towel fibers in the wells or wells drying out completely.

7- Add 100 μL of 1X Detection Antibody solution (refer to Reagent Preparation7.2) to each well. Seal plate with coverseal and incubate for 1 hour at 37°C.

8- Repeat wash step.

9- Add 100 μL of 1X HRP-conjugated antibody solution to each well. Seal plate with coverseal and incubate the plate for 40 minutes at 37°C .

10- Repeat wash step.

11- Signal development: Add 100 µL of TMB substrate solution to each well, protected from light. Incubate for 20 minutes. Substrate Solution should remain colorless until added to the plate.
12- Add 100 µL of Stop Solution to each well in the same order as addition of the TMB substrate. Mix by tapping the side of the plate gently.
13- Immediately after adding Stop solution read the absorbance on a microplate reader at a wavelength of 450nm.
14- Calculate the average of the duplicate readings (OD value) for each standard and sample and subtract the average of the zero standard absorbance. Construct a standard curve by plotting the mean absorbance for each standard on the y-axis

3- Activation of Caspases

- **Caspase-3 activation assay**

ab285337 – Human Caspase-3 ELISA Kit

Allow all reagents to reach room temperature before use. Gently mix all liquid reagents prior to use. Determine the number of 8-well strips needed for the assay. Insert these in the frame(s) for current use. Add 100 µl of the *Standard Diluent Buffer* to the zero standard wells. Well(s) reserved for chromogen blank should be left empty. Add 100 µl of standards and controls or diluted samples to the appropriate microtiter wells. The sample dilution chosen should be optimized for each experimental system. Tap gently on side of plate to mix. Cover wells with *plate cover* and incubate for 2 hours at room temperature. Thoroughly aspirate or decant solution from wells and discard the liquid, Wash wells 4 times. Pipette 100 µl of *Caspase-3 (Active) Detection Antibod*y solution into each well except the chromogen blank(s). Tap gently on the side of the plate to mix. Cover plate with *plate cover* and incubate for 1 hour at room temperature. Thoroughly aspirate or decant solution from wells and discard the liquid, Wash wells 4 times. Add 100 µl Anti-Rabbit IgG HRP Working Solution to each well except the chromogen blank(s). Prepare the working dilution as described in Preparing IgG HRP. Cover wells with the *plate cover* and incubate for 30 minutes at room temperature. Thoroughly aspirate or decant solution from wells and discard the liquid. Wash wells 4 times. Add 100 µl of *Stabilized Chromogen* to each well. The liquid in the wells will begin to turn blue. Incubate for 30 minutes at room temperature and in the dark. The incubation time for chromogen substrate is often determined by the microtiter plate reader used. Many plate readers have the capacity to record a maximum optical density (O.D.) of 2.0. The O.D. values should be monitored and the substrate reaction stopped before the O.D. of the positive wells exceeds the limits of the instrument. The O.D. values at 450 nm can only be read after the *Stop Solution* has been added to each well. If using a reader that records only to 2.0 O.D., stopping the assay after 20 to 25 minutes is suggested. Add 100 µl of *Stop Solution* to each well. Tap side of plate gently to mix. The solution in the wells should change from blue to yellow. Read the absorbance of each well at 450 nm having blanked the plate reader against a chromogen blank composed of 100 µl each of *Stabilized Chromogen* and *Stop Solution*. Read the plate within 2 hours after adding the *Stop Solution*.

Use a curve fitting software to generate the standard curve. A four-parameter algorithm provides the best standard curve fit. Read the concentrations for unknown samples and controls from the standard curve. Multiply value(s) obtained for sample(s) by the appropriate dilution factor to correct for the dilution in step 3. Samples producing signals greater than that of the highest standard should be diluted in *Standard Diluent Buffer* and reanalyzed.

- **Caspases-8 activation assay**
  Human Caspase 8 (CASP8) ELISA Kit
  Catalog No: MBS452285

Cells were obtained from American Type Culture Collection, cells were grown in RPMI 1640 containing 10% fetal bovine serum at 37°C, stimulated with the compounds to be tested for caspase-8, and lysed with Cell Extraction Buffer. This lysate was diluted in Standard Diluent Buffer over the range of the assay and measured for human active caspase-8 content. (*cells are Plated in a density of 1.2 – 1.8 × 10,000 cells/well in a volume of 100µl complete growth medium + 100 ul of the tested compound per well in a 96-well plate for 24 hours before the enzyme assay*). The absorbance of each microwell was read on a spectro-photometer at 450 nm. A standard curve is prepared from 7 human Caspases standard dilutions and human Caspase-8 concentration determined.

4- Effects on BAX and Bcl-2 proteins

- **Bax activation assay**
  Catalog No : E-EL-H0562
  Product size: 96T/48T/24T/96T*5
  Human BAX(Bcl-2 Associated X Protein) ELISA Kit

Bring all reagents, except the human Bax-α Standard, to room temperature for at least 30 minutes prior to opening. The human Bax-α Standard solution should not be left at room temperature for more than 10 minutes. All standards, controls and samples should be run in duplicate. Refer to the Assay Layout Sheet to determine the number of wells to be used and put any remaining wells with the desiccant back into the pouch and seal the ziploc. Store unused wells at 4 °C. Pipet 100 µL of Assay Buffer into the S0 (0 pg/mL standard) wells. Pipet 100 µL of Standards #1 through #6 into the appropriate wells. Pipet 100 µL of the Samples into the appropriate wells. Tap the plate gently to mix the contents. Seal the plate and incubate at room temperature on a plate shaker for 1 hour at ~500 rpm. Empty the contents of the wells and wash by adding 400 µL of wash solution to every well. Repeat the wash 4 more times for a total of **5 washes**. After the final wash, empty or aspirate the wells and firmly tap the plate on a lint free

paper towel to remove any remaining wash buffer. Pipet 100 µL of yellow Antibody into each well, except the Blank. Seal the plate and incubate at room temperature on a plate shaker for 1 hour at ~500 rpm. Empty the contents of the wells and wash by adding 400 µL of wash solution to every well. Repeat the wash 4 more times for a total of **5 washes**. After the final wash, empty or aspirate the wells and firmly tap the plate on a lint free paper towel to remove any remaining wash buffer. Add 100 µL of blue Conjugate to each well, except the

Blank. Seal the plate and incubate at room temperature on a plate shaker for 30 minutes at ~500 rpm. Empty the contents of the wells and wash by adding 400 µL of wash solution to every well. Repeat the wash 4 more times for a total of **5 washes**. After the final wash, empty or aspirate the wells and firmly tap the plate on a lint free paper towel to remove any remaining wash buffer. Pipet 100 µL of Substrate Solution into each well. Incubate for 30 minutes at room temperature on a plate shaker at ~500 rpm. Pipet 100 µL Stop Solution to each well. Blank the plate reader against the Blank wells, read the optical density at 450 nm. Calculate the average net Optical Density (OD) bound for each standard and sample by subtracting the average Blank OD from the average OD for each standard and sample. Using linear graph paper, plot the Average Net OD for each standard versus Bax concentration in each standard. Approximate a straight line through the points. The concentration of Bax in the unknowns can be determined by interpolation.

- **Bcl-2 inhibition assay**
  Human Bcl-2 ELISA Kit
  Enzyme-linked Immunosorbent Assay for quantitative detection of human Bcl-2
  Catalog Numbers BMS244-3 and BMS244-3TEN

Mix all reagents thoroughly without foaming before use. Wash the microwells twice with approximately 300 µL Wash Buffer per well with thorough aspiration of microwell contents between washes. Take caution not to scratch the surface of the microwells. After the last wash, empty the wells and tap microwell strips on absorbent pad or paper towel to remove excess Wash Buffer. Use the microwell strips immediately after washing or place upside down on a wet absorbent paper for not longer than 15 minutes. Do not allow wells to dry. Add 100 µL of Sample Diluent in duplicate to all standard wells and to the blank wells. Prepare standard (1:2 dilution) in duplicate ranging from 32 ng/mL to 0.5 ng/mL. Add 100 µL of Sample Diluent, in duplicate, to the blank wells. Add 80 µL of Sample Diluent, in duplicate, to the sample wells. Add 20 µL of each Sample, in duplicate, to the designated wells. Add 50 µL of diluted biotin-conjugate to all wells, including the blank wells. Cover with a plate cover and incubate at room temperature, on a microplate shaker at 100 rpm if available, for 2 hours. Remove plate cover and empty the wells. Wash microwell strips 3 times as described in step 2. Add 100 µL of diluted Streptavidin-HRP to all wells, including the blank wells. Cover with a plate cover and incubate at room temperature, on a microplate shaker at 100 rpm if available, for 1 hour. Remove plate cover and empty the wells. Wash microwell strips 3 times as described in step 2. Proceed to the next step. Pipette 100 µl of mixed TMB Substrate Solution to all wells, including the blanks. Incubate the microwell strips at room temperature (18° to 25°C) for about 15 minutes, if available on a rotator set at 100 rpm. Avoid direct exposure to intense light. The point, at which the substrate reaction is stopped, is often determined by the ELISA reader. Many ELISA readers record absorbance only up to 2.0 O.D. Therefore, the color development within individual microwells must be watched by the person running the assay and the substrate reaction stopped before positive wells are no longer properly detectable. Stop the enzyme reaction by quickly pipetting 100 µL of Stop Solution into each well, including the blank wells. It is important that the Stop

Solution is spread quickly and uniformly throughout the microwells to completely inactivate the enzyme. Results must be read immediately after the Stop Solution is added or within one hour if the microwell strips are stored at 2 - 8°C in the dark. Read absorbance of each microwell on a spectrophotometer using 450 nm as the primary wave length.

5- CD1 inhibitory assay

G1/S-specific cyclin-D1 (CCND1) (Human) ELISA Kit 01/17 (Catalog # E4287-100, 100 assays, Store at 4°C)

- **Application**:
  This ELISA kit is used for in vitro quantitative determination of Human CCND1.

  Detection Range: 0.313 - 20 ng/ml

  Sensitivity: < 0.188 ng/ml

  Assay Precision: Intra-Assay: CV < 8%; Inter-Assay: CV < 10% (CV (%) = SD/mean X 100)

- **Sample Type:** Human serum, plasma, tissue homogenates and other biological fluids.
- **Assay Protocol:** Note: Bring all reagents and samples to room temperature 30 minutes prior to the assay. It is recommended that all standards and samples be run at least in duplicate. A standard curve must be run with each assay.

  1. Prepare all reagents, samples and standards as instructed in section VII.

  2. Wash plate 2 times with **1X Wash Solution** before adding standard, sample and control wells.

  3. Add 100 µl of each **standards** or **samples** into appropriate wells. Cover well and incubate for 1.5 hours at 37ºC.

  4. Remove the cover and discard the plate content without washing or letting the wells completely dry.

  5. Add 0.1 ml of **Biotin-detection antibody** work solution into the above wells. Seal the plate and incubate at 37ºC for 60 min.

  6. Discard the solution and wash 3 times with **1X Wash Solution**. Wash by filling each well with Wash Buffer (350 µl) using a multi-channel pipette or autowasher. Let it soak for 1-2 minutes, and then remove all residual wash-liquid from the wells by aspiration. After the last wash, remove any remaining Wash Buffer by aspirating or decanting. Clap the plate on absorbent filter papers or other absorbent materials.

  7. Add 0.1 ml of **SABC working solution** into each well, cover the plate and incubate at 37ºC for 30 min.

  8. Discard the solution and wash 5 times with **1X Wash Solution** as step 6.

9. Add 90 µl of **TMB substrate** into each well, cover the plate and incubate at 37 ºC in dark within 15-30 min. The shades of blue should be seen in the first 3-4 wells by the end of incubation.

10. Add 50 µl of **Stop Solution** to each well. Read result at 450 nm within 20 minutes.

6- Molecular Docking

- **Method of Molecular Docking.**

In the recent study, the co-crystallized ligand of crystal protein (PDB code: 2XDX) obtained from the RCSB was used to generate the binding sites. The targeted proteins were prepared by removing water molecules, adding missing amino acids, correcting unfilled valence atoms, and minimizing the protein peptide energy by applying CHARMM force fields. The essential amino acids of the protein were selected and prepared for screening. The tested compounds were prepared by drawing their 2D structures using Chem-Bio Draw Ultra17.0 and saving them in SDF file format. The tested ligands were protonated, and energy was minimized using MMFF94 force field with 0.1 RMSD kcal/mol. The minimized structures were stored for molecular docking. Molecular docking was performed using docking algorithms of Autodock Vina 4.0, where the target pocket was held rigid, and the ligands were allowed to be flexible. Each molecule was allowed to produce twenty different interaction poses with the protein during the refinement. The docking scores (affinity interaction energy) of the best-fitted poses with the active site of the HSP90 were recorded and the 3D orientation was generated using Discovery Studio 2016 visualizer software.

- **Method of molecular dynamic simulation study**

The Desmond simulation package from Schrödinger LLC was utilized for conducting molecular dynamics (MD) simulations [1]. The NPT ensemble, with a temperature set at 300 K and a pressure of 1 bar, was consistently employed across all runs. The simulations ran for 100 ns, with a relaxation time of 1 ps for the tested ligands. OPLS_2005 force field parameters were employed for all simulations. Long-range electrostatic interactions were computed using the particle mesh Ewald method, with a cutoff radius of 9.0 Å for Coulomb interactions [2].

- Water molecules were explicitly represented using the simple point charge model. Pressure control utilized the Martyna–Tuckerman–Klein chain coupling scheme with a coupling constant of 2.0 ps, while temperature control employed the Nosé–Hoover chain coupling scheme. Nonbonded forces were calculated using an r-RESPA integrator, with short-range forces updated every step and long-range forces updated every three steps. Trajectories were saved at 4.8 ps intervals for subsequent analysis.
- The behavior and interactions between ligands and proteins were examined using the Simulation Interaction Diagram tool within the Desmond MD package. The stability of

MD simulations was assessed by monitoring the root mean square deviation (rmsd) of ligand and protein atom positions over time.

- Additionally, the AMBER 14 package[3], with the AMBER force field ff99, was employed for various tasks, including minimization, addition of counterions, solvation, equilibration, and running periodic box, explicit water (TIP4P) MD simulations for the tested ligands. The structures of the tested ligands were optimized using the density functional theory B3LYP method with a 6-31G basis set, and parameters were set to the GAFF force field. The protein–ligand–water system was allowed to move freely during simulations, which comprised 10 independent runs with different random initial velocities. Each run spanned 10 ns, utilizing a timestep of 0.001 ps (1 fs). These multiple MD simulations, recognized for their wide acceptance, could adequately sample conformational space compared to longer, single-trajectory simulations. Data analysis was conducted using the cpptraj program from the AMBER Tools distribution

Raw material of biological assays

1- Average of Relative viability of cells (%)

A- MCF-7 cell line assay

Sample Code: (2)

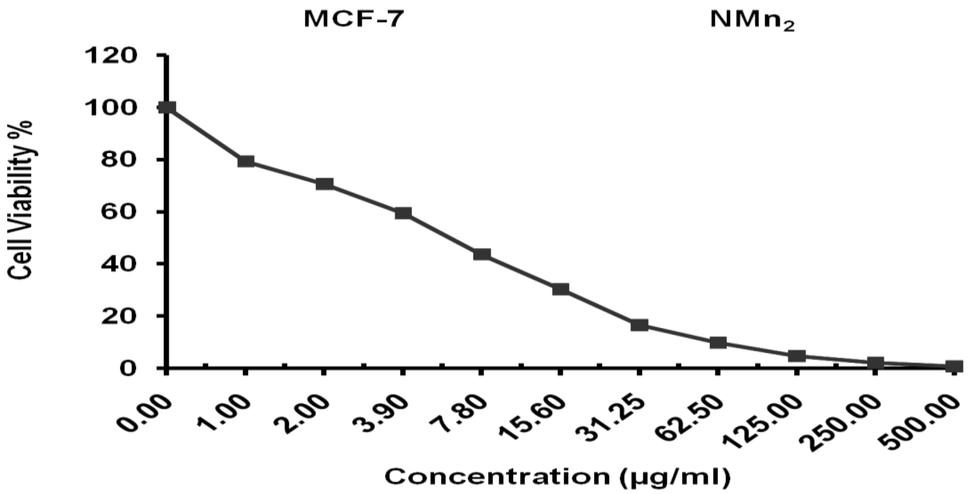

Sample conc. (μg/ml)	Viability %	Inhibitory %	S.D. (±)

500	0.74	99.26	0.12
250	2.09	97.91	0.07
125	4.72	95.28	0.14
62.5	9.85	90.15	0.31
31.25	16.54	83.46	0.28
15.6	30.28	69.72	0.74
7.8	43.71	56.29	1.95
3.9	59.43	40.57	0.62
2	70.69	29.31	0.89
1	79.36	20.64	0.42
0	100	0	

***Comment*:**

Inhibitory activity against Breast carcinoma cells was detected using MTT assay under these experimental conditions with IC_{50} = 6.24 ± 0.39 µg/ml.

Sample Code: (3)

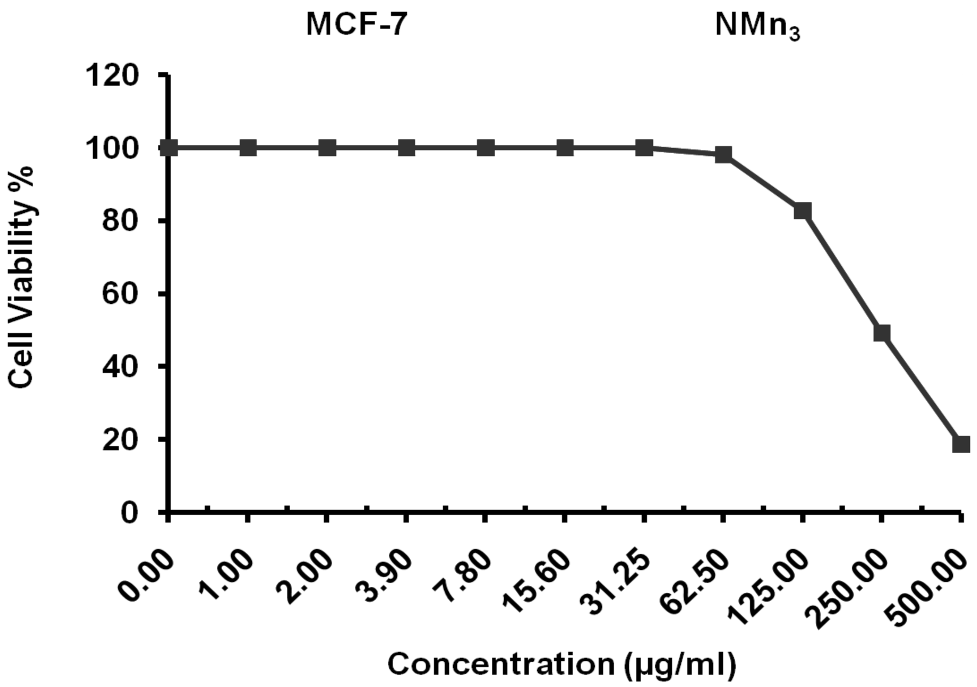

Sample conc. (µg/ml)	Viability %	Inhibitory %	S.D. (±)
500	18.79	81.21	1.85
250	49.27	50.73	2.36
125	82.76	17.24	1.82
62.5	98.05	1.95	0.63
31.25	100	0	
15.6	100	0	
7.8	100	0	
3.9	100	0	
2	100	0	
1	100	0	
0	100	0	

***<u>Comment</u>*:**

Inhibitory activity against Breast carcinoma cells was detected using MTT assay under these experimental conditions with IC_{50} = 247.28 ± 8.14 µg/ml.

Sample Code: (4)

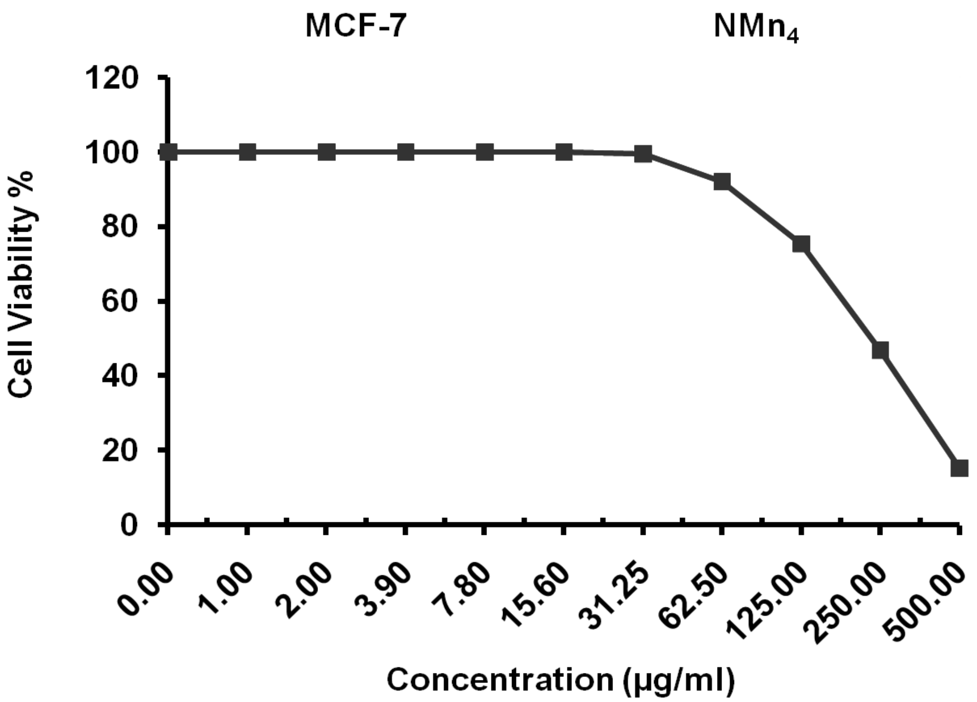

Sample conc. (µg/ml)	Viability %	Inhibitory %	S.D. (±)
500	15.28	84.72	1.06
250	46.89	53.11	2.37
125	75.43	24.57	1.91
62.5	92.12	7.88	0.46
31.25	99.47	0.53	0.19
15.6	100	0	
7.8	100	0	
3.9	100	0	
2	100	0	
1	100	0	
0	100	0	

***Comment*:**

Inhibitory activity against Breast carcinoma cells was detected using MTT assay under these experimental conditions with IC_{50} = 236.38 ± 6.93 µg/ml.

Sample Code: (5)

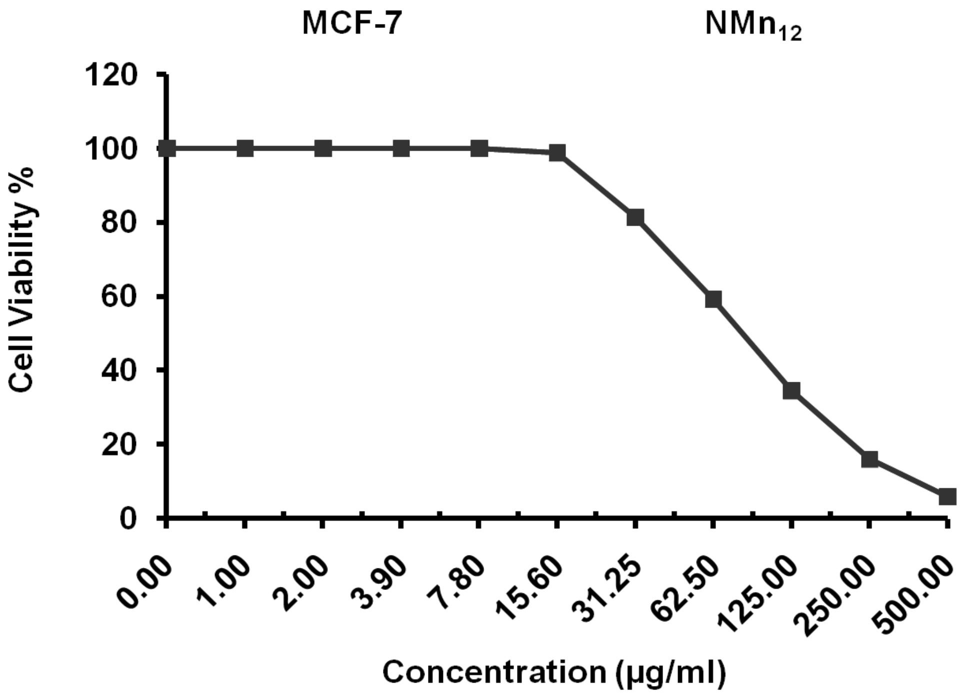

Sample conc. (µg/ml)	Viability %	Inhibitory %	S.D. (±)
500	5.94	94.06	0.32
250	16.08	83.92	0.46
125	34.51	65.49	1.43
62.5	59.27	40.73	2.31
31.25	81.34	18.66	1.72
15.6	98.76	1.24	0.18
7.8	100	0	
3.9	100	0	
2	100	0	

1	100	0	
0	100	0	

Comment:

Inhibitory activity against Breast carcinoma cells was detected using MTT assay under these experimental conditions with IC_{50} = 85.90 ± 3.82 µg/ml.

Sample Code: (6)

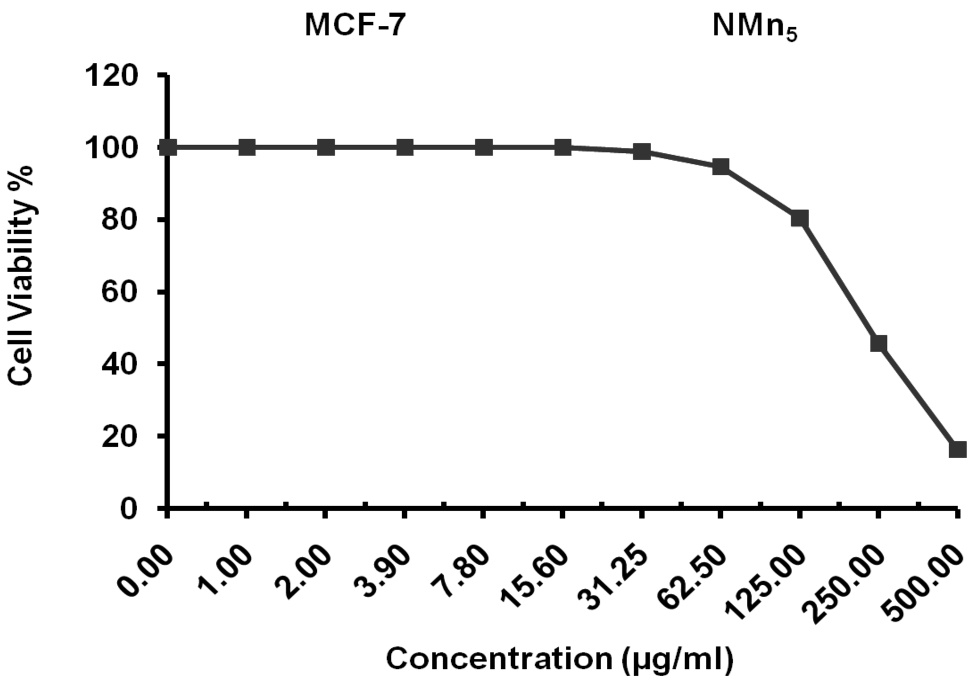

Sample conc. (µg/ml)	Viability %	Inhibitory %	S.D. (±)
500	16.29	83.71	1.35
250	45.71	54.29	1.83
125	80.36	19.64	1.22
62.5	94.63	5.37	0.59
31.25	98.80	1.2	0.24
15.6	100	0	
7.8	100	0	

3.9	100	0	
2	100	0	
1	100	0	
0	100	0	

***Comment*:**

Inhibitory activity against Breast carcinoma cells was detected using MTT assay under these experimental conditions with IC_{50} = 234.52±6.48 µg/ml.

Sample Code: (7)

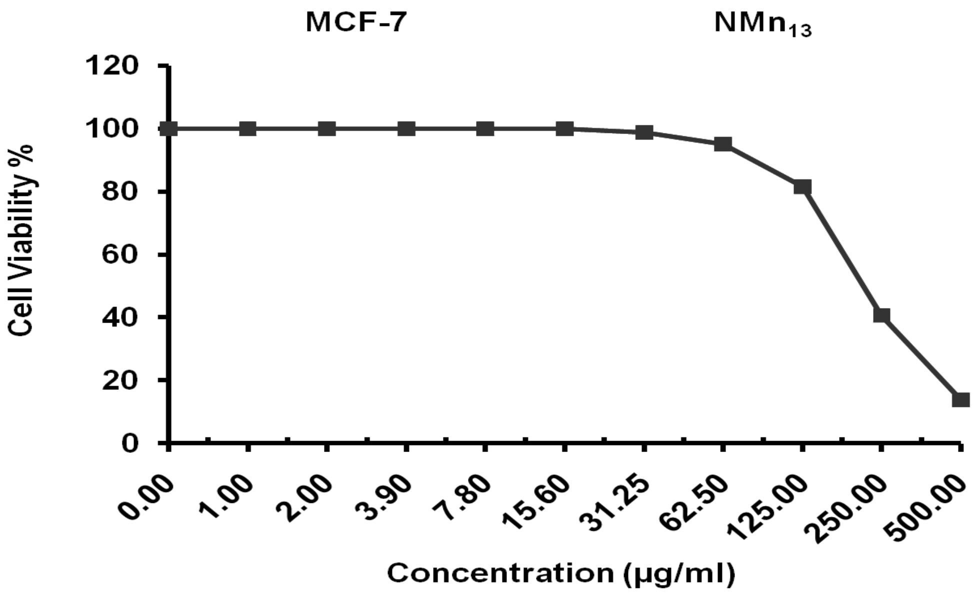

Sample conc. (µg/ml)	Viability %	Inhibitory %	S.D. (±)
500	13.72	86.28	1.86
250	40.65	59.35	2.31
125	81.49	18.51	2.73
62.5	95.06	4.94	0.82
31.25	98.79	1.21	0.45
15.6	100	0	

7.8	100	0	
3.9	100	0	
2	100	0	
1	100	0	
0	100	0	

***Comment*:**

Inhibitory activity against Breast carcinoma cells was detected using MTT assay under these experimental conditions with IC_{50} = 221.38 ± 6.35 µg/ml.

Sample Code: (8)

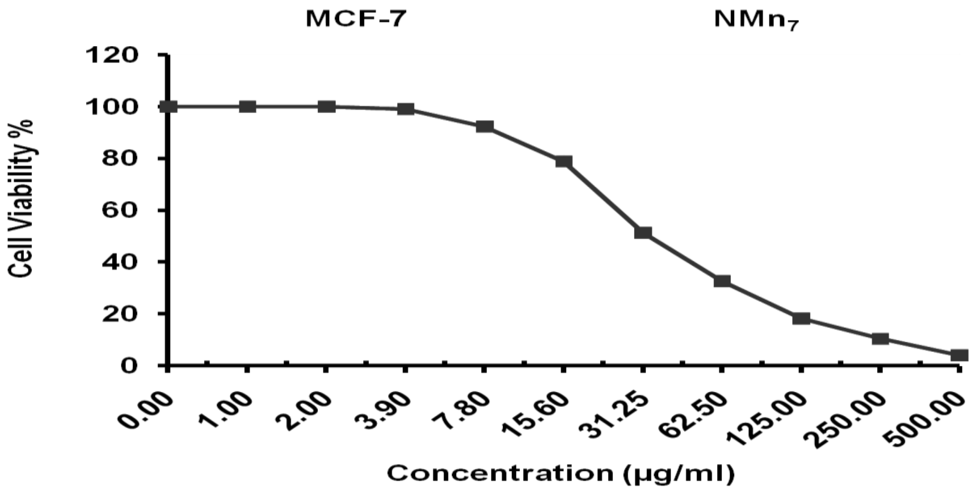

Sample conc. (µg/ml)	Viability %	Inhibitory %	S.D. (±)
500	4.06	95.94	0.22
250	10.47	89.53	0.34
125	18.23	81.77	0.85
62.5	32.65	67.35	1.91
31.25	51.40	48.6	1.32
15.6	78.91	21.09	1.75

7.8	92.36	7.64	0.28
3.9	99.02	0.98	0.34
2	100	0	
1	100	0	
0	100	0	

***Comment*:**

Inhibitory activity against Breast carcinoma cells was detected using MTT assay under these experimental conditions with IC_{50} = 33.58 ± 1.98 µg/ml.

Sample Code: (9)

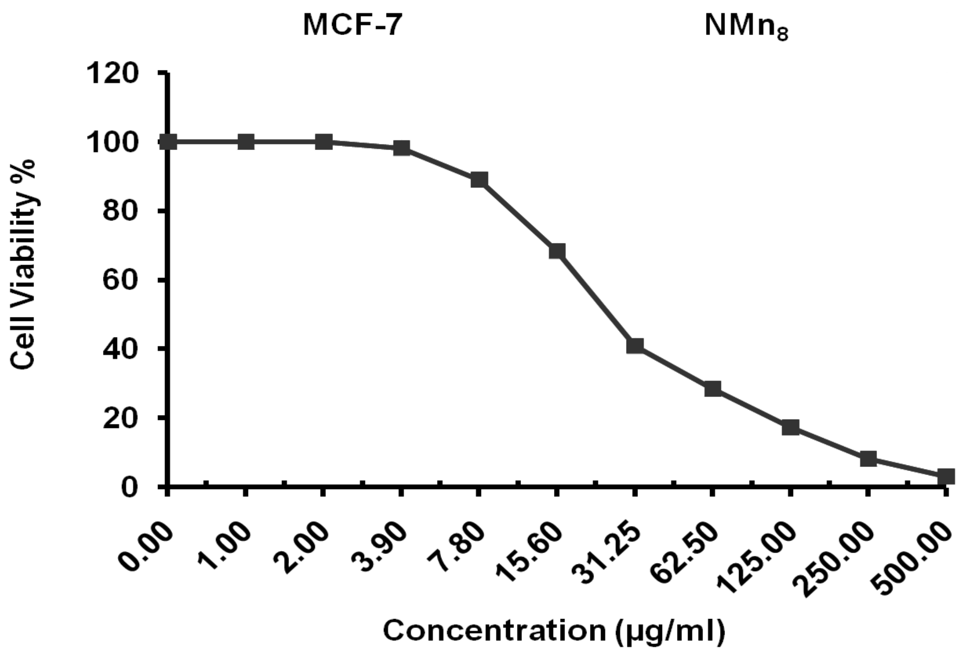

Sample conc. (µg/ml)	Viability %	Inhibitory %	S.D. (±)
500	3.14	96.86	0.12
250	8.29	91.71	0.37
125	17.45	82.55	1.09
62.5	28.62	71.38	2.46

31.25	40.97	59.03	1.85
15.6	68.43	31.57	2.11
7.8	89.06	10.94	1.42
3.9	98.23	1.77	0.61
2	100	0	
1	100	0	
0	100	0	

***Comment*:**

Inhibitory activity against Breast carcinoma cells was detected using MTT assay under these experimental conditions with IC_{50} = 26.07± 1.23 µg/ml.

Sample Code: (10)

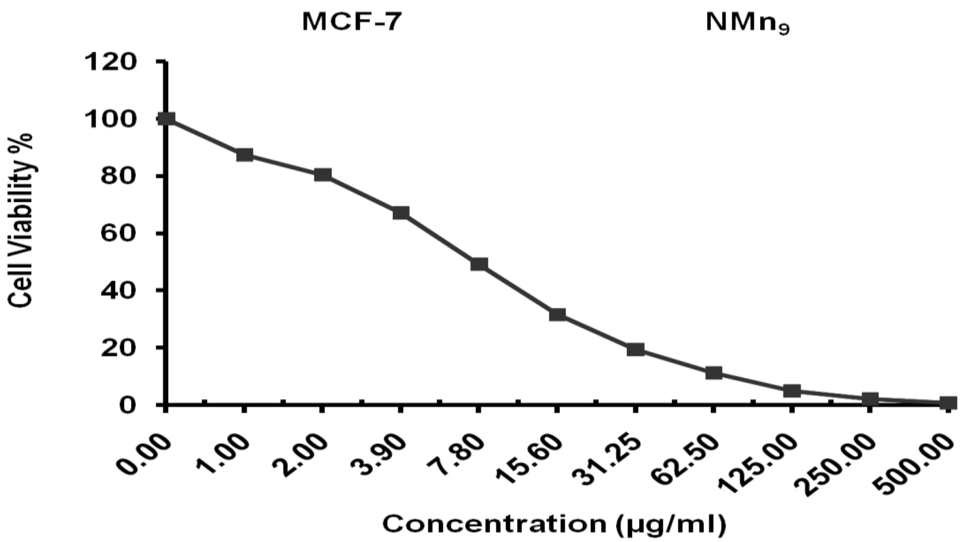

Sample conc. (µg/ml)	Viability %	Inhibitory %	S.D. (±)
500	0.89	99.11	0.07
250	2.31	97.69	0.13
125	5.08	94.92	0.16
62.5	11.47	88.53	0.31

31.25	19.56	80.44	0.42
15.6	31.74	68.26	1.28
7.8	49.28	50.72	1.79
3.9	67.15	32.85	1.37
2	80.42	19.58	0.64
1	87.39	12.61	0.23
0	100	0	

Comment:

Inhibitory activity against Breast carcinoma cells was detected using MTT assay under these experimental conditions with IC_{50} = 7.64 ± 0.53 μg/ml.

Sample Code: (11)

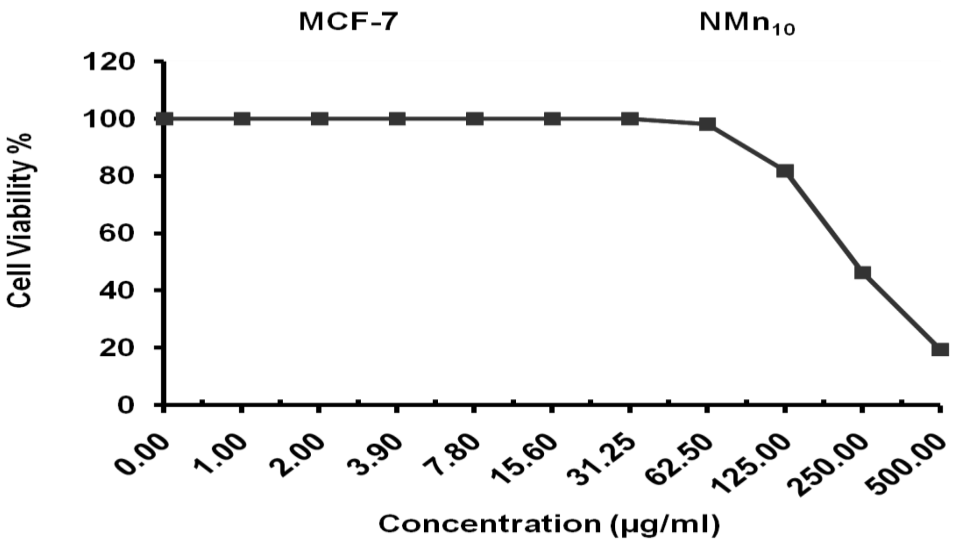

Sample conc. (μg/ml)	Viability %	Inhibitory %	S.D. (±)
500	19.48	80.52	2.06
250	46.37	53.63	1.95
125	81.76	18.24	1.32
62.5	98.04	1.96	0.68

31.25	100	0	
15.6	100	0	
7.8	100	0	
3.9	100	0	
2	100	0	
1	100	0	
0	100	0	

***Comment*:**

Inhibitory activity against Breast carcinoma cells was detected using MTT assay under these experimental conditions with IC_{50} = 237.18±7.04 µg/ml.

Sample Code: (12)

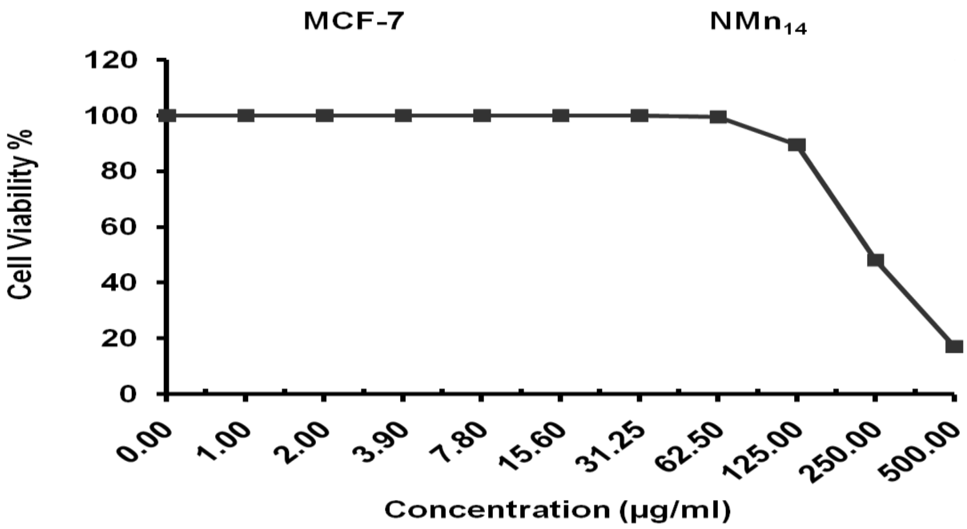

Sample conc. (µg/ml)	Viability %	Inhibitory %	S.D. (±)
500	17.02	82.98	1.44
250	48.15	51.85	1.95
125	89.53	10.47	2.01
62.5	99.46	0.54	0.29
31.25	100	0	

15.6	100	0	
7.8	100	0	
3.9	100	0	
2	100	0	
1	100	0	
0	100	0	

***Comment*:**

Inhibitory activity against Breast carcinoma cells was detected using MTT assay under these experimental conditions with IC_{50} = 244.41 ± 7.95 µg/ml.

B- MDA-231 cell line assay

Conc.(µM)	MDA-231
DOX	
100	4.1
50	11.8
25	16.2
12.5	27.3
6.25	36.1
3.125	52.6
1.56	61.7
A4	
100	17.7
50	25.8
25	34.4
12.5	46.2
6.25	69.6
3.125	86.5
1.56	100
A6	
100	26.9
50	42.8
25	57.2
12.5	72.6
6.25	84.3
3.125	100

1.56	100
A7	
100	45.0
50	57.1
25	72.3
12.5	84.4
6.25	97.5
3.125	100
1.56	100
A12	
100	37.5
50	53.1
25	69.1
12.5	80.3
6.25	94.2
3.125	100
1.56	100
A8	
100	30.8
50	46.2
25	57.0
12.5	70.8
6.25	92.3
3.125	100
1.56	100
A10	
100	8.7
50	17.3
25	26.5
12.5	32.6
6.25	61.4
3.125	70.2
1.56	91.1
A5	
100	18.2
50	32.8
25	43.6
12.5	55.1
6.25	72.4
3.125	93.3
1.56	100
A11	
100	22.6
50	36.4
25	51.2
12.5	64.9
6.25	72.5

3.125	91.7
1.56	100
A3	
100	40.7
50	59.8
25	71.1
12.5	85.2
6.25	100
3.125	100
1.56	100
A9	
100	7.3
50	12.1
25	16.8
12.5	28.7
6.25	46.0
3.125	58.5
1.56	72.4
A13	
100	46.6
50	61.8
25	73.5
12.5	88.7
6.25	97.9
3.125	100
1.56	100
A2	
100	33.6
50	48.7
25	60.8
12.5	76.2
6.25	97.9
3.125	100
1.56	100

2- HSP90 assay

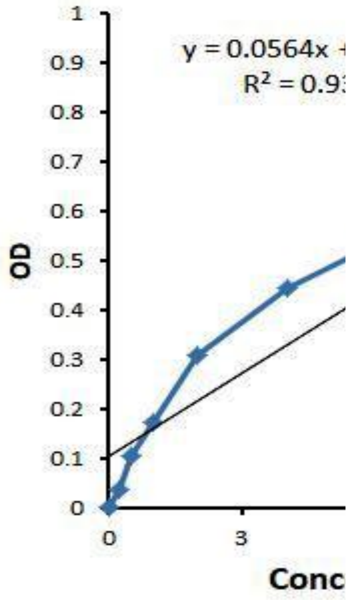

HPS90 Standard curve

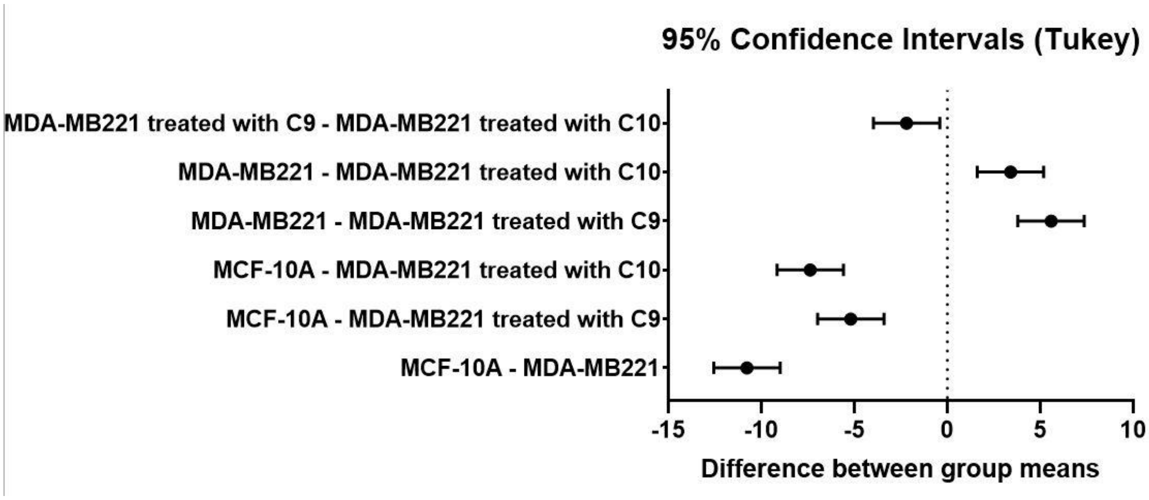

HSP90 confidence

P value	<0.0001				
P value summary	****				
Significant diff. among means (P	Yes				
R square	0.9801				
**Brown-Forsythe test**					
F (DFn, DFd)	0.1630 (3, 8)				
P value	0.9183				
P value summary	ns				
Are SDs significantly different (P	No				
**Bartlett's test**					
Bartlett's statistic (corrected)					
P value					
P value summary					
Are SDs significantly different (P					
**ANOVA table**	**SS**	**DF**	**MS**	**F (DFn, DFd)**	**P value**
Treatment (between columns)	183.5	3	61.16	F (3, 8) = 131	P<0.0001
Residual (within columns)	3.725	8	0.4656		
Total	187.2	11			

Number of families							
Number of comparisons per family							
Alpha							
**Tukey's multiple comparisons test**	**95.00% CI of diff.**	**Significant?**	**Summary**	**Adjusted P Value**			
**MCF-10A** vs. **MDA-MB221**	-12.55 to -8.985	Yes	****	<0.0001	B-C		
**MCF-10A** vs. **MDA-MB221 treated with C9**	-6.968 to -3.400	Yes	****	<0.0001	B-D		
**MCF-10A** vs. **MDA-MB221 treated with C10**	-9.149 to -5.581	Yes	****	<0.0001	B-E		
**MDA-MB221** vs. **MDA-MB221 treated with C9**	3.801 to 7.369	Yes	****	<0.0001	C-D		
**MDA-MB221** vs. **MDA-MB221 treated with C10**	1.620 to 5.188	Yes	**	0.0013	C-E		
**MDA-MB221 treated with C9** vs. **MDA-MB221 treated**	-3.965 to -0.3966	Yes	*	0.0187	D-E		
**Test details**	**Mean 2**	**Mean Diff.**	**SE of diff.**	**n1**	**n2**	**q**	**DF**
**MCF-10A** vs. **MDA-MB221**	15.15	-10.77	0.5572	3	3	27.33	8
**MCF-10A** vs. **MDA-MB221 treated with C9**	9.567	-5.184	0.5572	3	3	13.16	8
**MCF-10A** vs. **MDA-MB221 treated with C10**	11.75	-7.365	0.5572	3	3	18.69	8
**MDA-MB221** vs. **MDA-MB221 treated with C9**	9.567	5.585	0.5572	3	3	14.18	8
**MDA-MB221** vs. **MDA-MB221 treated with C10**	11.75	3.404	0.5572	3	3	8.641	8
**MDA-MB221 treated with C9** vs. **MDA-MB221 treated**	11.75	-2.181	0.5572	3	3	5.536	8

	MCF-10A	MDA-MB221	MDA-MB221 treated with C9	MDA-MB221 treated with C10
Number of values	3	3	3	3
Minimum	3.560	14.80	8.863	10.96
25% Percentile	3.560	14.80	8.863	10.96
Median	4.395	14.98	9.555	12.04
75% Percentile	5.193	15.67	10.28	12.25
Maximum	5.193	15.67	10.28	12.25
Mean	4.383	15.15	9.567	11.75
Std. Deviation	0.8167	0.4591	0.7093	0.6941
Std. Error of Mean	0.4715	0.2650	0.4095	0.4007
Lower 95% CI	2.354	14.01	7.805	10.02
Upper 95% CI	6.412	16.29	11.33	13.47

HSP90 results

3- Caspase-3 assay

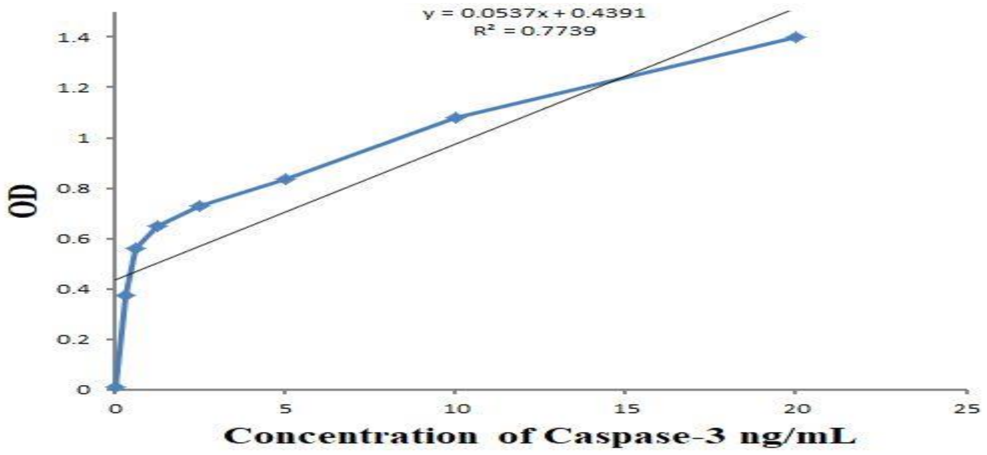

Caspase-3 stander curve

P value	<0.0001				
P value summary	****				
Significant diff. among means (P	Yes				
R square	0.9651				
**Brown-Forsythe test**					
F (DFn, DFd)	0.6704 (3, 8)				
P value	0.5937				
P value summary	ns				
Are SDs significantly different (P	No				
**Bartlett's test**					
Bartlett's statistic (corrected)					
P value					
P value summary					
Are SDs significantly different (P					
**ANOVA table**	**SS**	**DF**	**MS**	**F (DFn, DFd)**	**P value**
Treatment (between columns)	84.08	3	28.03	F (3, 8) = 73.	P<0.0001
Residual (within columns)	3.045	8	0.3806		
Total	87.13	11			

P value	<0.0001				
P value summary	****				
Significant diff. among means (P	Yes				
R square	0.9651				
**Brown-Forsythe test**					
F (DFn, DFd)	0.6704 (3, 8)				
P value	0.5937				
P value summary	ns				
Are SDs significantly different (P	No				
**Bartlett's test**					
Bartlett's statistic (corrected)					
P value					
P value summary					
Are SDs significantly different (P					
**ANOVA table**	**SS**	**DF**	**MS**	**F (DFn, DFd)**	**P value**
Treatment (between columns)	84.08	3	28.03	F (3, 8) = 73.	P<0.0001
Residual (within columns)	3.045	8	0.3806		
Total	87.13	11			

	MCF-10A	MDA-MB221	MDA-MB221 treated with C9	MDA-MB221 treated with C10
Number of values	3	3	3	3
Minimum	4.709	1.730	8.825	7.894
25% Percentile	4.709	1.730	8.825	7.894
Median	5.641	2.642	9.402	8.155
75% Percentile	6.609	2.847	9.644	8.490
Maximum	6.609	2.847	9.644	8.490
Mean	5.653	2.406	9.290	8.180
Std. Deviation	0.9501	0.5946	0.4208	0.2988
Std. Error of Mean	0.5485	0.3433	0.2429	0.1725
Lower 95% CI	3.293	0.9292	8.245	7.437
Upper 95% CI	8.013	3.883	10.34	8.922

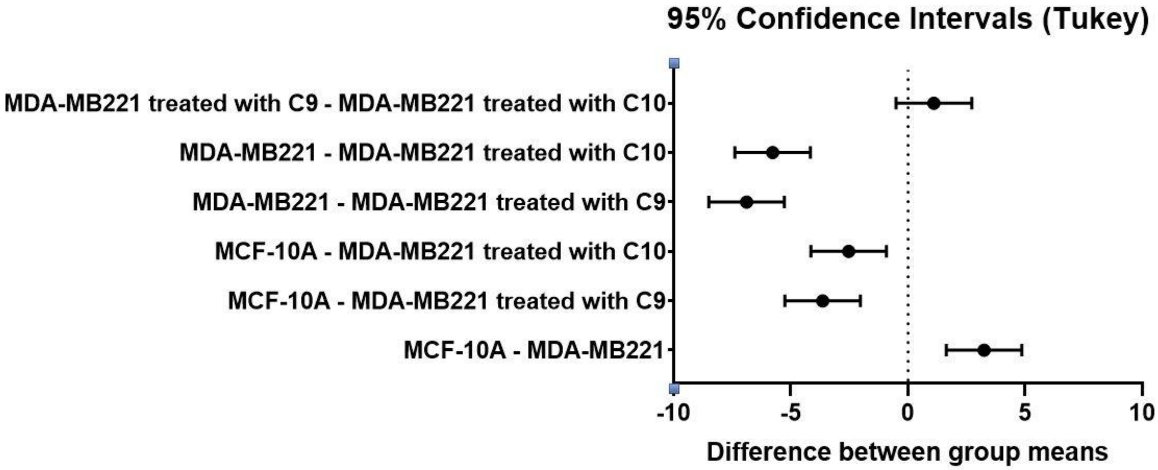

4- Caspase 8 assay

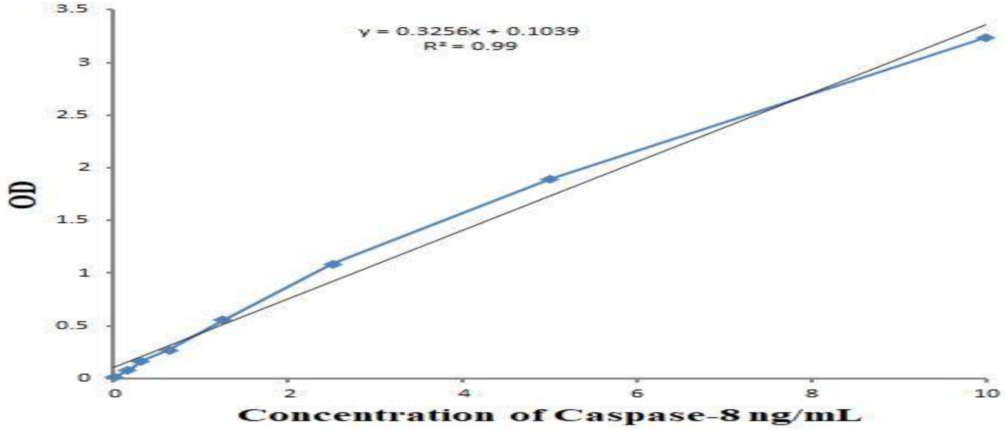

Caspase-8 stander curve

Table Analyzed	Data 1
Data sets analyzed	B-E
**ANOVA summary**	
F	236.8
P value	<0.0001
P value summary	****
Significant diff. among means (P < 0.05)?	Yes
R square	0.9889
**Brown-Forsythe test**	
F (DFn, DFd)	0.4714 (3, 8)
P value	0.7106
P value summary	ns
Are SDs significantly different (P < 0.05)?	No
**Bartlett's test**	
Bartlett's statistic (corrected)	
P value	
P value summary	
Are SDs significantly different (P < 0.05)?	

Number of families						
Number of comparisons per family						
Alpha						
**Tukey's multiple comparisons test**	**Significant?**	**Summary**	**Adjusted P Value**			
**MCF-10A** vs. **MDA-MB221**	Yes	****	<0.0001	B-C		
**MCF-10A** vs. **MDA-MB221 treated with C9**	Yes	***	0.0002	B-D		
**MCF-10A** vs. **MDA-MB221 treated with C10**	Yes	****	<0.0001	B-E		
**MDA-MB221** vs. **MDA-MB221 treated with C9**	Yes	****	<0.0001	C-D		
**MDA-MB221** vs. **MDA-MB221 treated with C10**	Yes	****	<0.0001	C-E		
**MDA-MB221 treated with C9** vs. **MDA-MB221 treated**	Yes	*	0.0296	D-E		
**Test details**	**Mean Diff.**	**SE of diff.**	**n1**	**n2**	**q**	**DF**
**MCF-10A** vs. **MDA-MB221**	3.358	0.2658	3	3	17.87	8
**MCF-10A** vs. **MDA-MB221 treated with C9**	-2.204	0.2658	3	3	11.73	8
**MCF-10A** vs. **MDA-MB221 treated with C10**	-3.155	0.2658	3	3	16.79	8
**MDA-MB221** vs. **MDA-MB221 treated with C9**	-5.562	0.2658	3	3	29.60	8
**MDA-MB221** vs. **MDA-MB221 treated with C10**	-6.513	0.2658	3	3	34.66	8
**MDA-MB221 treated with C9** vs. **MDA-MB221 treated**	-0.9510	0.2658	3	3	5.060	8

		MCF-10A	MDA-MB221	MDA-MB221 treated with C9	MDA-MB221 treated with C10
Number of values		3	3	3	3
Minimum		4.822	1.736	7.338	8.314
25% Percentile		4.822	1.736	7.338	8.314
Median		5.446	1.865	7.546	8.505
75% Percentile		5.771	2.365	7.768	8.686
Maximum		5.771	2.365	7.768	8.686
Mean		5.346	1.989	7.551	8.502
Std. Deviation		0.4823	0.3322	0.2150	0.1860
Std. Error of Mean		0.2784	0.1918	0.1242	0.1074
Lower 95% CI		4.148	1.163	7.016	8.040
Upper 95% CI		6.544	2.814	8.085	8.964

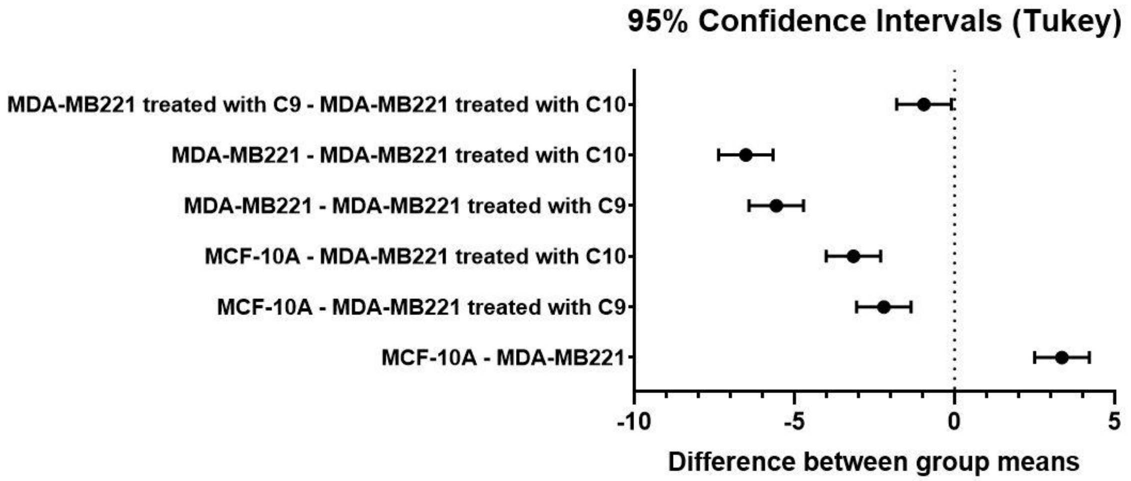

5- BAX assay

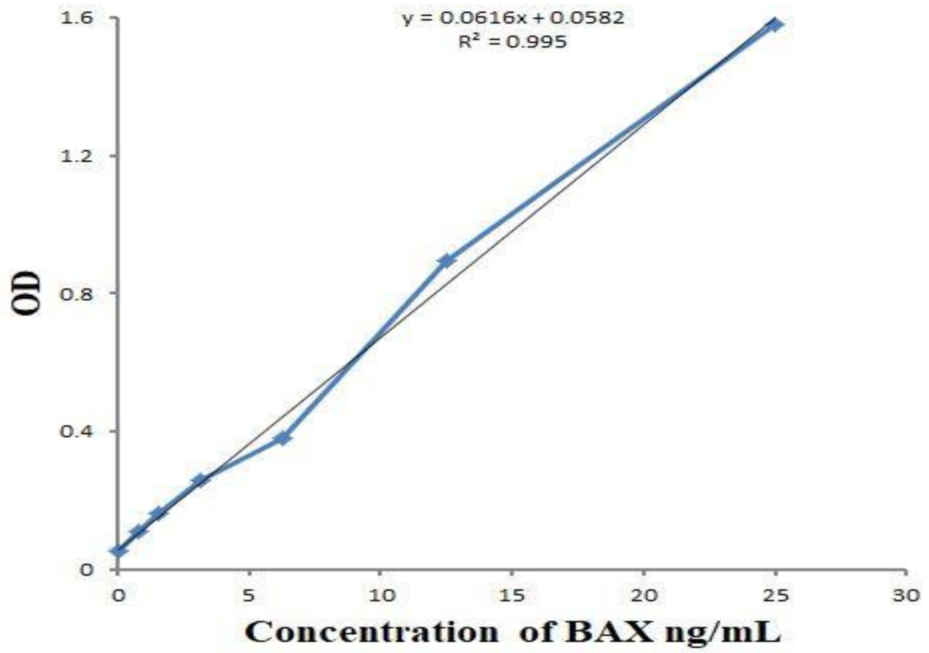

SC of BAX

ANOVA summary					
F	92.30				
P value	<0.0001				
P value summary	****				
Significant diff. among means (P < 0.05)?	Yes				
R square	0.9719				
**Brown-Forsythe test**					
F (DFn, DFd)	0.8497 (3, 8)				
P value	0.5047				
P value summary	ns				
Are SDs significantly different (P < 0.05)?	No				
**Bartlett's test**					
Bartlett's statistic (corrected)					
P value					
P value summary					
Are SDs significantly different (P < 0.05)?					
**ANOVA table**	**SS**	**DF**	**MS**	**F (DFn, DFd)**	**P value**
Treatment (between columns)	79.36	3	26.45	F (3, 8) = 92.30	P<0.0001
Residual (within columns)	2.293	8	0.2866		
Total	81.65	11			

Number of comparisons per family						
Alpha						
**Tukey's multiple comparisons test**	**Significant?**	**Summary**	**Adjusted P Value**			
**MCF-10A** vs. **MDA-MB221**	Yes	****	<0.0001	B-C		
**MCF-10A** vs. **MDA-MB221 treated with C9**	Yes	*	0.0441	B-D		
**MCF-10A** vs. **MDA-MB221 treated with C10**	Yes	**	0.0012	B-E		
**MDA-MB221** vs. **MDA-MB221 treated with C9**	Yes	****	<0.0001	C-D		
**MDA-MB221** vs. **MDA-MB221 treated with C10**	Yes	****	<0.0001	C-E		
**MDA-MB221 treated with C9** vs. **MDA-MB221 treated**	No	ns	0.0742	D-E		
**Test details**	**Mean Diff.**	**SE of diff.**	**n1**	**n2**	**q**	**DF**
**MCF-10A** vs. **MDA-MB221**	6.894	0.4371	3	3	22.30	8
**MCF-10A** vs. **MDA-MB221 treated with C9**	1.439	0.4371	3	3	4.656	8
**MCF-10A** vs. **MDA-MB221 treated with C10**	2.716	0.4371	3	3	8.789	8
**MDA-MB221** vs. **MDA-MB221 treated with C9**	-5.455	0.4371	3	3	17.65	8
**MDA-MB221** vs. **MDA-MB221 treated with C10**	-4.177	0.4371	3	3	13.52	8
**MDA-MB221 treated with C9** vs. **MDA-MB221 treated**	1.277	0.4371	3	3	4.133	8

Number of comparisons per family						
Alpha						
**Tukey's multiple comparisons test**	**Significant?**	**Summary**	**Adjusted P Value**			
**MCF-10A** vs. **MDA-MB221**	Yes	****	<0.0001	B-C		
**MCF-10A** vs. **MDA-MB221 treated with C9**	Yes	*	0.0441	B-D		
**MCF-10A** vs. **MDA-MB221 treated with C10**	Yes	**	0.0012	B-E		
**MDA-MB221** vs. **MDA-MB221 treated with C9**	Yes	****	<0.0001	C-D		
**MDA-MB221** vs. **MDA-MB221 treated with C10**	Yes	****	<0.0001	C-E		
**MDA-MB221 treated with C9** vs. **MDA-MB221 treated**	No	ns	0.0742	D-E		
**Test details**	**Mean Diff.**	**SE of diff.**	**n1**	**n2**	**q**	**DF**
**MCF-10A** vs. **MDA-MB221**	6.894	0.4371	3	3	22.30	8
**MCF-10A** vs. **MDA-MB221 treated with C9**	1.439	0.4371	3	3	4.656	8
**MCF-10A** vs. **MDA-MB221 treated with C10**	2.716	0.4371	3	3	8.789	8
**MDA-MB221** vs. **MDA-MB221 treated with C9**	-5.455	0.4371	3	3	17.65	8
**MDA-MB221** vs. **MDA-MB221 treated with C10**	-4.177	0.4371	3	3	13.52	8
**MDA-MB221 treated with C9** vs. **MDA-MB221 treated**	1.277	0.4371	3	3	4.133	8

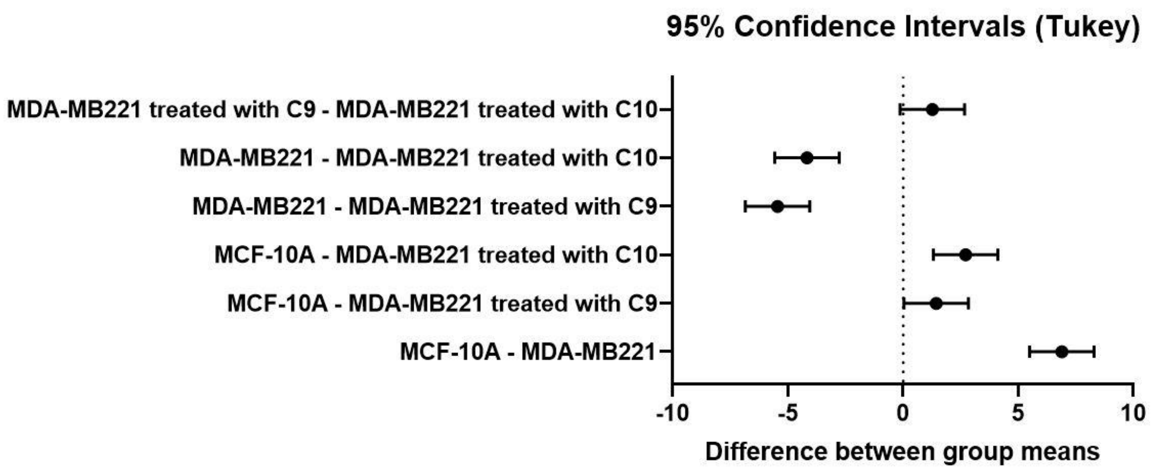

6- BCl2 Assay

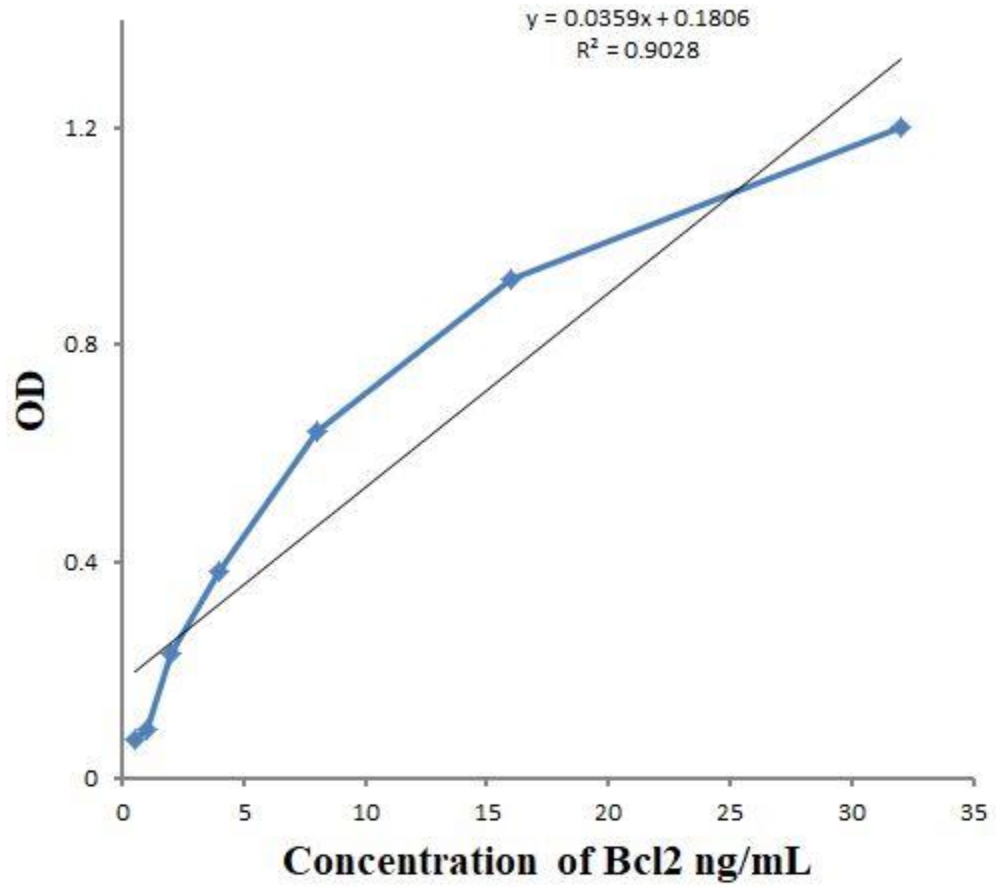

BCl2 SC

Bartlett's test					
Bartlett's statistic (corrected)					
P value					
P value summary					
Are SDs significantly different (P < 0.05)?					
**ANOVA table**	**SS**	**DF**	**MS**	**F (DFn, DFd)**	**P value**
Treatment (between columns)	861.7	3	287.2	F (3, 8) = 782	P<0.0001
Residual (within columns)	2.938	8	0.3673		
Total	864.6	11			
**Data summary**					
Number of treatments (columns)	4				
Number of values (total)	12				

Number of families	1							
Number of comparisons per family	6							
Alpha	0.05							
**Tukey's multiple comparisons test**	**Mean Diff.**	**95.00% CI of diff.**	**Significant?**	**Summary**	**Adjusted P Value**			
**MCF-10A** vs. **MDA-MB221**	-23.05	-24.63 to -21.46	Yes	****	<0.0001	B-C		
**MCF-10A** vs. **MDA-MB221 treated with C9**	-15.92	-17.51 to -14.34	Yes	****	<0.0001	B-D		
**MCF-10A** vs. **MDA-MB221 treated with C10**	-9.573	-11.16 to -7.988	Yes	****	<0.0001	B-E		
**MDA-MB221** vs. **MDA-MB221 treated with C9**	7.122	5.537 to 8.707	Yes	****	<0.0001	C-D		
**MDA-MB221** vs. **MDA-MB221 treated with C10**	13.47	11.89 to 15.06	Yes	****	<0.0001	C-E		
**MDA-MB221 treated with C9** vs. **MDA-MB221 treated**	6.351	4.766 to 7.935	Yes	****	<0.0001	D-E		
**Test details**	**Mean 1**	**Mean 2**	**Mean Diff.**	**SE of diff.**	**n1**	**n2**	**q**	**DF**
**MCF-10A** vs. **MDA-MB221**	5.536	28.58	-23.05	0.4948	3	3	65.87	8
**MCF-10A** vs. **MDA-MB221 treated with C9**	5.536	21.46	-15.92	0.4948	3	3	45.51	8
**MCF-10A** vs. **MDA-MB221 treated with C10**	5.536	15.11	-9.573	0.4948	3	3	27.36	8
**MDA-MB221** vs. **MDA-MB221 treated with C9**	28.58	21.46	7.122	0.4948	3	3	20.35	8
**MDA-MB221** vs. **MDA-MB221 treated with C10**	28.58	15.11	13.47	0.4948	3	3	38.51	8
**MDA-MB221 treated with C9** vs. **MDA-MB221 treated**	21.46	15.11	6.351	0.4948	3	3	18.15	8

	MCF-10A	MDA-MB221	MDA-MB221 treated with C9	MDA-MB221 treated with C10
Number of values	3	3	3	3
Minimum	4.969	28.26	20.93	14.33
25% Percentile	4.969	28.26	20.93	14.33
Median	5.749	28.51	21.35	14.97
75% Percentile	5.889	28.98	22.10	16.03
Maximum	5.889	28.98	22.10	16.03
Mean	5.536	28.58	21.46	15.11
Std. Deviation	0.4957	0.3682	0.5929	0.8581
Std. Error of Mean	0.2862	0.2126	0.3423	0.4954
Lower 95% CI	4.304	27.67	19.99	12.98
Upper 95% CI	6.767	29.50	22.93	17.24

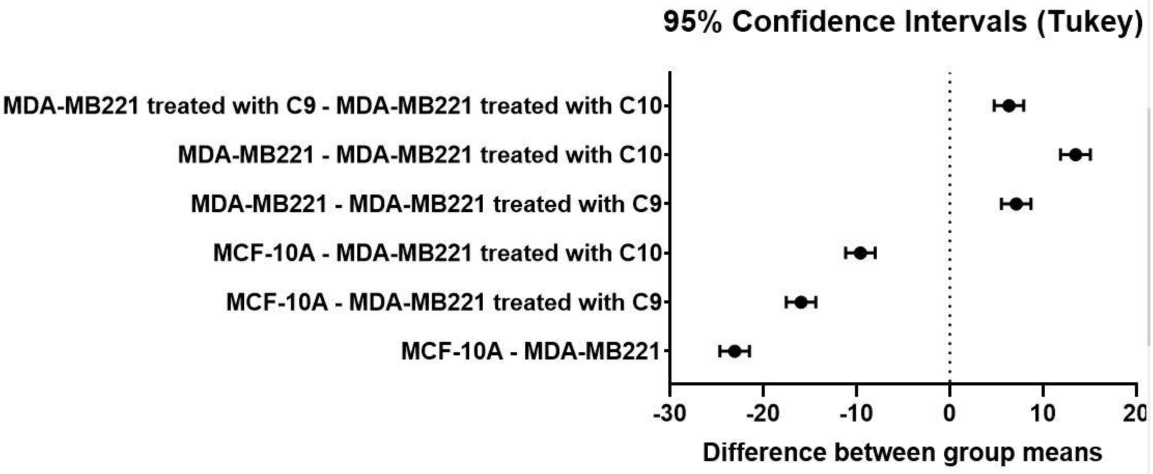

7- CD1 assay

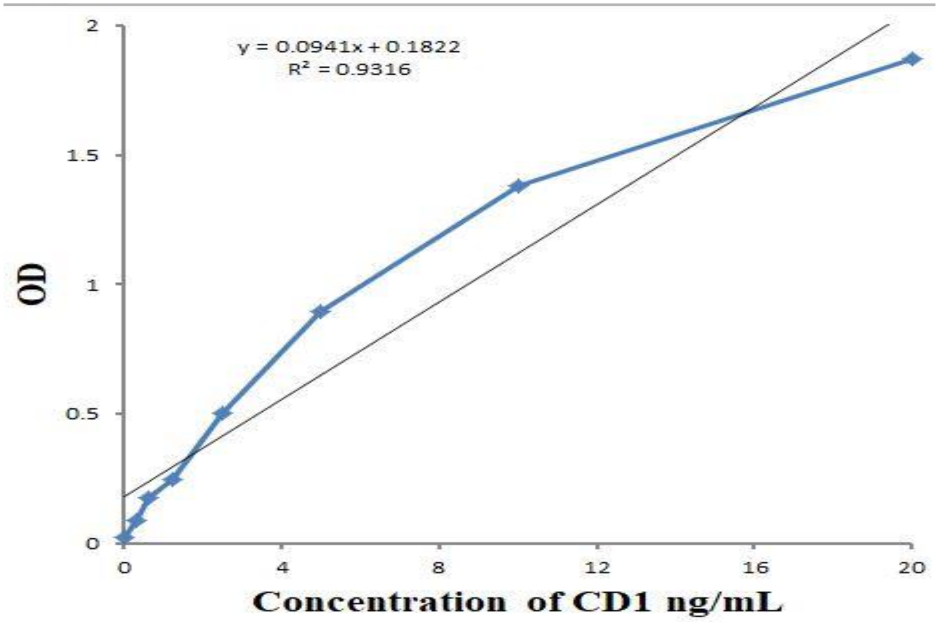

CD-1 SC

**ANOVA summary**					
F	69.80				
P value	<0.0001				
P value summary	****				
Significant diff. among means (P < 0.05)?	Yes				
R square	0.9632				
**Brown-Forsythe test**					
F (DFn, DFd)	0.3117 (3, 8)				
P value	0.8167				
P value summary	ns				
Are SDs significantly different (P < 0.05)?	No				
**Bartlett's test**					
Bartlett's statistic (corrected)					
P value					
P value summary					
Are SDs significantly different (P < 0.05)?					
**ANOVA table**	**SS**	**DF**	**MS**	**F (DFn, DFd)**	**P value**
Treatment (between columns)	571.8	3	190.6	F (3, 8) = 69.80	P<0.0001

Number of comparisons per family						
Alpha						
**Tukey's multiple comparisons test**	**Significant?**	**Summary**	**Adjusted P Value**			
**MCF-10A** vs. **MDA-MB221**	Yes	****	<0.0001	B-C		
**MCF-10A** vs. **MDA-MB221 treated with C9**	Yes	*	0.0462	B-D		
**MCF-10A** vs. **MDA-MB221 treated with C10**	Yes	**	0.0023	B-E		
**MDA-MB221** vs. **MDA-MB221 treated with C9**	Yes	****	<0.0001	C-D		
**MDA-MB221** vs. **MDA-MB221 treated with C10**	Yes	***	0.0002	C-E		
**MDA-MB221 treated with C9** vs. **MDA-MB221 treated**	No	ns	0.1673	D-E		
**Test details**	**Mean Diff.**	**SE of diff.**	**n1**	**n2**	**q**	**DF**
**MCF-10A** vs. **MDA-MB221**	-18.67	1.349	3	3	19.57	8
**MCF-10A** vs. **MDA-MB221 treated with C9**	-4.396	1.349	3	3	4.608	8
**MCF-10A** vs. **MDA-MB221 treated with C10**	-7.556	1.349	3	3	7.919	8
**MDA-MB221** vs. **MDA-MB221 treated with C9**	14.28	1.349	3	3	14.96	8
**MDA-MB221** vs. **MDA-MB221 treated with C10**	11.12	1.349	3	3	11.65	8
**MDA-MB221 treated with C9** vs. **MDA-MB221 treated**	-3.160	1.349	3	3	3.312	8

		**MCF-10A**	**MDA-MB221**	**MDA-MB221 treated with C9**	**MDA-MB221 treated with C10**
Number of values		3	3	3	3
Minimum		6.448	24.61	10.58	12.46
25% Percentile		6.448	24.61	10.58	12.46
Median		7.639	26.48	12.16	15.84
75% Percentile		8.627	27.64	13.17	17.08
Maximum		8.627	27.64	13.17	17.08
Mean		7.571	26.24	11.97	15.13
Std. Deviation		1.091	1.528	1.302	2.388
Std. Error of Mean		0.6299	0.8824	0.7515	1.379
Lower 95% CI		4.861	22.45	8.734	9.195
Upper 95% CI		10.28	30.04	15.20	21.06

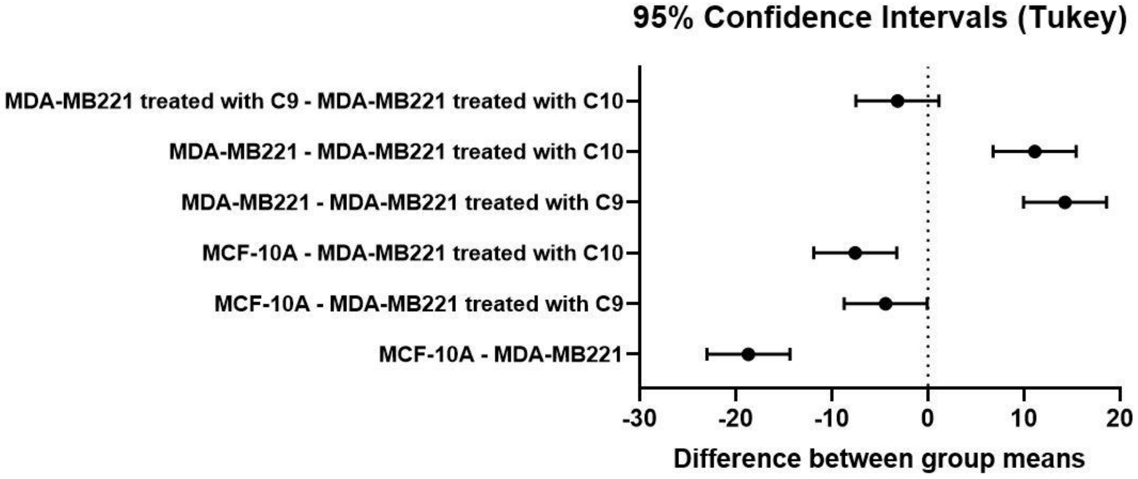

HSp90 inhibitory results

Researcher	:	Dr. Mohammed Elbastawesy email: Moh.organic@yahoo.com **mob.** 01004292549
Date	:	28/08/2024
Assay	:	HSP90 activity assay
Samples	:	03 samples
Reference	:	---
Cell line	:	---
[illegible]	[illegible]	[illegible]

Lab Report

ser	compound		HSP90	SD
	code	M.W g/mol	IC50 uM	
**1**	**s9**	**291**	**2.432**±0.078	
**2**	**s10**	**274**	**7.305**±0.235	
***	Novobiocin	**612.62**	**1.135**±0.036	

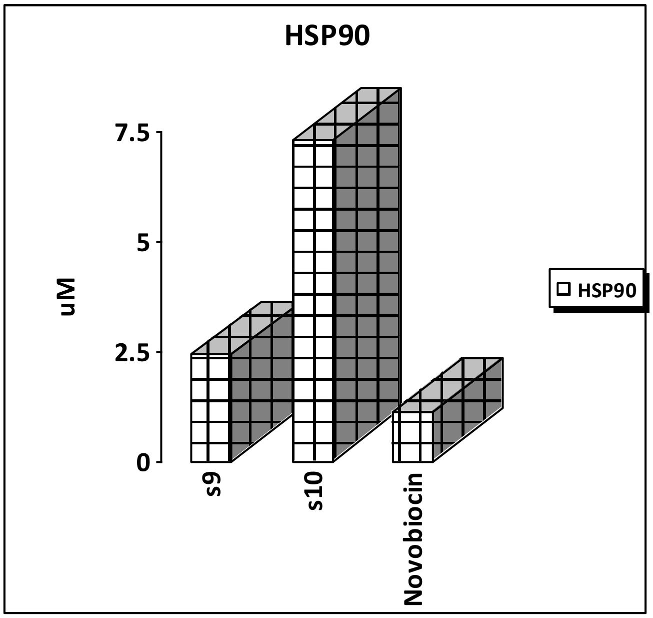

Detailed Results:

HSP90		[redacted]								[redacted]		
code	IC50	conc	log	%inh	T2	T1	ΔT	RFU2	RFU1	ΔRFU	slope	K.Activity
s9		100	2	83	15	0	15	62976	0	62976	25217.7	9.989174
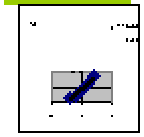		10	1	62	15	0	15	143859	0	143859	25217.7	22.81873
		1	0	37	15	0	15	237902	0	237902	25217.7	37.73572
		0.1	-1	21	15	0	15	298543	0	298543	25217.7	47.35452
		0.01	-2	10	15	0	15	338614	0	338614	25217.7	53.71053
EC				0	15	0	15	378266	0	378266	25217.7	60
		[redacted]		[redacted]			[redacted]		[redacted]		[redacted]	

code	IC50	conc	log	%inh	T2	T1	ΔT	RFU2	RFU1	ΔRFU	slope	K.Activity
s10		100	2	76	15	0	15	89494	0	89494	25217.7	14.19543
		10	1	55	15	0	15	171532	0	171532	25217.7	27.20819
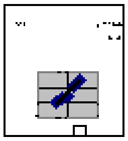		1	0	22	15	0	15	295722	0	295722	25217.7	46.90705
		0.1	-1	12	15	0	15	331806	0	331806	25217.7	52.63065
		0.01	-2	5	15	0	15	359471	0	359471	25217.7	57.01884
EC				0	15	0	15	378266	0	378266	25217.7	60
		[redacted]		[redacted]			[redacted]		[redacted]		[redacted]	

code	IC50	conc	log	%inh	T2	T1	ΔT	RFU2	RFU1	ΔRFU	slope	K.Activity
Novobiocin		100	2	84	15	0	15	61384	0	61384	25217.7	9.736653
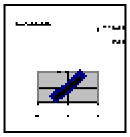		10	1	68	15	0	15	121933	0	121933	25217.7	19.34086
		1	0	45	15	0	15	208752	0	208752	25217.7	33.11198
		0.1	-1	31	15	0	15	262543	0	262543	25217.7	41.64424
		0.01	-2	18	15	0	15	308494	0	308494	25217.7	48.93293
EC				0	15	0	15	378266	0	378266	25217.7	60
ET790M		[redacted]		[redacted]			[redacted]		[redacted]		[redacted]	

**s9**	y = 18.663x + 42.797
**s10**	y = 18.512x + 34.013
**Novobiocin**	y = 16.783x + 49.078

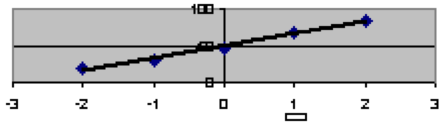

Supplement: Supplementary file 1 [file pharmaceuticals-17-01284-s001.zip › pharmaceuticals-3113208-supplementary.pdf]
